# Modern history of hypoxia in Narragansett Bay: The geochemical record

**DOI:** 10.1016/j.scitotenv.2024.176007

**Published:** 2024-09-02

**Authors:** Warren S. Boothman, Laura Coiro

**Affiliations:** U.S. Environmental Protection Agency, Office of Research and Development, Center for Environmental Measurement and Modeling, Atlantic Coastal Environmental Sciences Division, 27 Tarzwell Drive, Narragansett, RI 02882, USA

**Keywords:** Molybdenum, Carbon, Nitrogen, Stable isotopes, Sediment cores, Hypoxia, Wastewater treatment

## Abstract

Increased inputs of nitrogen from agricultural runoff, urbanization and suburbanization have resulted in degradation of water quality, including increased frequency and severity of hypoxia, in estuarine ecosystems. Much work has been conducted in recent years to characterize the spatial and temporal extent of hypoxia in coastal systems, but the historical record of hypoxia in such systems is much less well known. The current work examines the history of hypoxia in upper Narragansett Bay, an urbanized estuary in the northeastern U.S., through vertical profiles of geochemical markers in sediment cores. Concentrations of authigenic molybdenum indicate more frequent/ longer periods of hypoxia that are related to changes in population and anthropogenic inputs to the Bay from the surrounding watersheds. Cores from the urbanized upper bay, greatly affected by wastewater treatment facilities (WWTFs), indicate greater duration of hypoxia in the 20th century, with periods of hypoxia decreasing through mid-century and recurring thereafter. Trends of hypoxia are closely related to improvements and failures of WWTFs in surrounding communities. In Greenwich Bay, with a suburban watershed and only one WWTF, hypoxia increased substantially in parallel with growth of population in the surrounding watershed. Carbon and nitrogen concentrations and isotope values reflect increased nitrogen enrichment and productivity in the Bay in the 2nd half of the 20th century. These results can help inform study of the environmental responses to societal activities that may affect water quality.

## Graphical Abstract

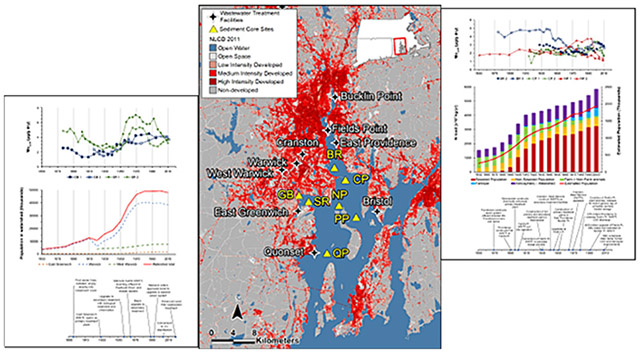

## 1. Introduction

Increased inputs of nitrogen and other nutrients to estuarine and marine ecosystems as a result of agricultural practices, urbanization and suburbanization have resulted in degradation of coastal and estuarine water quality, including increased frequency and severity of hypoxia and concomitant ecological effects such as reductions in ecological diversity (Breitburg et al., 2018; Bricker et al., 2007; Brush, 2009; Diaz, 2001; Diaz and Rosenberg, 1995; Hagy et al., 2004; Hale et al., 2016; Kemp et al., 2005; Latimer and Rego, 2010; Rabalais et al., 2001a). In urbanized areas such as the northeastern United States, much of the nitrogen load to estuaries comes from human sewage sources, both directly from wastewater treatment facilities or from septic systems through groundwater transport (CENR, 2003; Latimer and Charpentier, 2010; Whitall et al., 2007). As a result, efforts to reduce the progress and effects of eutrophication have often focused on reducing the nitrogen loads in wastewater treatment facilities effluents. In Narragansett Bay (RI, USA), legislative response to a massive fish kill in 2003 resulted in significant (> 50 %) reductions in the nitrogen loads from wastewater treatment plants that discharge into the Providence River at the head of Narragansett Bay (Deacutis et al., 2006; Narragansett Bay Estuary Program, 2017; RI Department of Environmental Management, 2005), and there has been ongoing research studying the ecological effects resulting from these reductions (Codiga et al., 2022; Oczkowski et al., 2018; Oviatt et al., 2017). These ongoing studies can illustrate the rate and extent of ecological recovery under reduced N loading for a recent, relatively short time period, as the recovery proceeds. At the same time, examination of historical markers of the effects of nitrogen loading, such as hypoxia, in sediment cores can provide insight into the ecological trajectory leading to current conditions and possibly the prospects for recovery.

Much work has been conducted in recent years to characterize the spatial and temporal extent of hypoxia in coastal systems (Breitburg et al., 2018; Diaz, 2001; Diaz and Rosenberg, 2008; Hagy et al., 2004; Jarvis et al., 2021; Le et al., 2016; Milbrandt et al., 2021; Rabalais et al., 2001b; Scavia et al., 2006; Suzuki, 2001; Verity et al., 2006). The historical record of hypoxia in these systems, including Narragansett Bay, is not as well known. The work presented here examines the historical prevalence of hypoxia in Narragansett Bay, an urbanized estuary in the northeastern U.S., by determining the vertical profiles of molybdenum (Mo), a geochemical marker of hypoxia, in sediments in the Bay. Prior study has shown Mo in marine surface sediments may be used as a qualitative / quantitative surrogate for direct measurement of hypoxic conditions in overlying waters: Mo accumulates in surface sediments when dissolved oxygen (DO) concentrations decrease below 2.8 mg/L, with the concentration of accumulated Mo related to length of time the hypoxic conditions persist (Adelson et al., 2001; Boothman et al., 2022; Boothman and Coiro, 2009). By examining profiles of Mo in sediment cores, we determined the temporal variation and trends of hypoxia (defined in this work as [DO] < 2.8 mg/L) in locations in upper parts of Narragansett Bay most prone to development of hypoxia. The current work represents the first study of Mo sediment profiles, and by extension, the historical prevalence of hypoxia in Narragansett Bay.

## 2. Methods

### 2.1. Study area

Narragansett Bay is a relatively shallow (mean depth of 8.3 m) estuary in Rhode Island (USA), partially divided by several islands into two branches referred to as the East and West Passages. The East Passage is deeper (16–48 m) and somewhat narrower whereas the West Passage is generally flat and shallow (6–16 m). Estuarine circulation results in oceanic waters generally flowing northward through the East Passage into the upper Bay, where they mix with waters from the Providence River and flow westward and southward through the West Passage (Kincaid et al., 2008). A shallow (3–5 m) sill separates the Providence River, which receives the large majority of the freshwater inflow into Narragansett Bay from several tributaries (the Seekonk, Woonasquatucket, Moshassuck and Pawtuxet Rivers), from the upper Bay. The Bay receives a low freshwater input relative to its volume, resulting in a narrow range of salinity (22–32 ppt). Average residence time of water in the Bay is about 40 days (Pilson, 1985). The Bay is partially to well-mixed, but some areas are subject to periodic, episodic stratification (Bergondo et al., 2005; Codiga et al., 2009). The northernmost extent of the Narragansett Bay watershed is more heavily urbanized.

Eight of the nine largest WWTFs in RI, representing >85 % of all WWTF effluent entering Narragansett Bay, empty either directly or via tributaries into the Providence River. (Narragansett Bay Estuary Program, 2017). Consequently, treated sewage comprises the largest single source of nutrients to Narragansett Bay, and nutrient concentrations exhibit a gradient from the upper Bay to Rhode Island Sound (Oviatt, 2008).

Greenwich Bay is a small embayment on the western edge of Narragansett Bay near the northern end of the West Passage. Freshwater inflow is small, limited to a few streams feeding into coves on the western and northern shores of the Bay and a WWTF that serves the town of East Greenwich and discharges into Greenwich Cove. Wastewater is the largest source of nutrients to Greenwich Bay, coming from the East Greenwich WWTF and groundwater carrying residential septic effluent (Latimer and Charpentier, 2010), but also from nutrient rich water from upper Narragansett Bay entrained into Greenwich Bay by tidal circulation. Circulation within Greenwich Bay is strongly wind-driven and at times develops a gyre in the inner portion of the Bay that can retain water within the Bay for up to a month (Rogers, 2008).

### 2.2. Sediment core collection

**Fig. 1.**
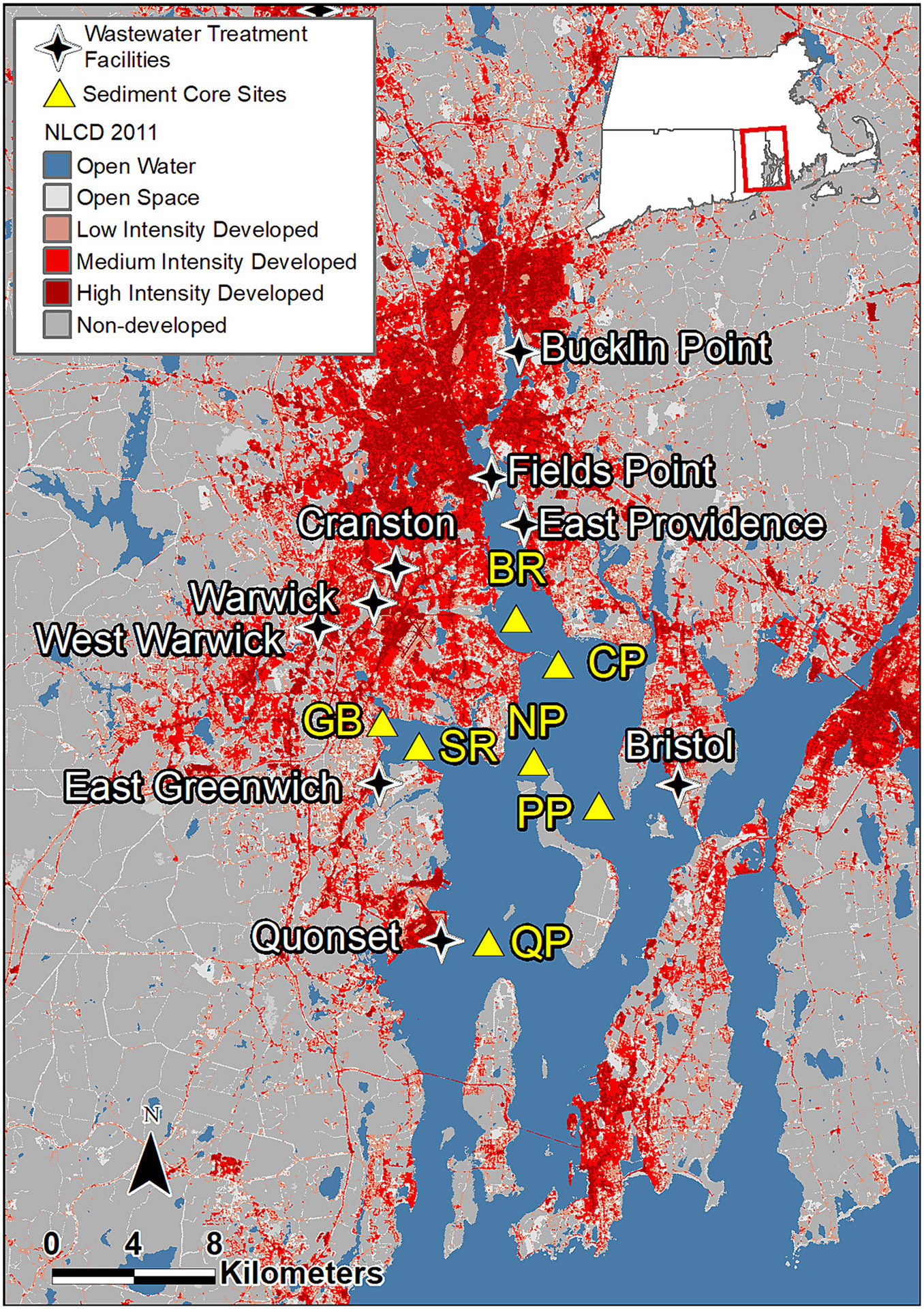
Locations of sediment core sampling sites (triangles), major wastewater treatment facilities (stars), and surrounding land use in Narragansett Bay, RI. Site codes: BR - Bullock’s Reach; CP - Conimicut Point; NP - North Prudence; PP - Poppasquash Point; GB - Greenwich Bay; SR - Sally Rock; QP – Quonset Point. See Table 1 for sampling site characteristics.

Sediment cores were collected in December 2011 and January 2012 from 7 sites in Narragansett Bay (RI, USA) encompassing a range of anthropogenic influences, with particular emphasis on the Upper Bay (Fig. 1). The sites were grouped geographically to represent different regions of the Bay: Bullock Reach (BR), Conimicut Point (CP), and North Prudence (NP) in the Upper Bay; Greenwich Bay (GB) and Sally Rock (SR) in Greenwich Bay, and Popasquash Point (PP) and Quonset Point (QP) as tidally upstream and downstream reference sites, respectively. The Bullock’s Reach site is located within the Providence River basin, downstream from 3 large WWTFs that release effluent directly into the River and the mouths of 3 rivers that receive effluent from 4 other large WWTFs; the Conimicut Point and North Prudence sites are located in open water outside the Providence River in the northernmost portion of Narragansett Bay.

Replicate cores were collected by gravity corer from each site, capped and stored vertically in ice onboard the ship, then frozen at −20 °C with overlying water intact upon return to the laboratory. Cores were subsequently sectioned into 1-cm horizons through the length of each core as in previous work (Boothman et al., 2022), placed into tracemetal free polypropylene jars and stored frozen until analysis.

### 2.3. Sediment accumulation rates

Sediment accumulation rates at the core sites were estimated from analysis of ^210^Pb concentrations in subsamples from selected core sections. The subsamples were freeze-dried and sieved through 400-μm nylon screen to remove shells and debris, packed and sealed in polycarbonate test tubes as in previous work, then delivered to University of Rhode Island Graduate School of Oceanography for gamma-counting (Boothman et al., 2022). Unsupported, or excess, ^210^Pb activity in each sample was calculated as:

(1) [Pb210]xs=[Pb210]total−[Ra226]

Excess ^210^Pb activity was present in the top 10 to 25 cm of each core, with a mixed layer of 2 to 4 cm at the Bullocks Reach (BR), Conimicut Point (CP) and Sally Rock (SR) sites.

Sediment accumulation rates for each site were calculated using 2 models. The Constant Input Concentration (CIC) model assumes a constant flux of unsupported ^210^Pb (the amount of ^210^Pb in excess of that produced within sediments by the decay of its parent isotope, ^226^Ra) to a sediment surface and relatively little post-depositional processes, physical or biological, that would redistribute excess ^210^Pb present in the sediment. Under such conditions, radionuclide activities decline monotonically with depth, with excess ^210^Pb activity declining exponentially (Swarzenski, 2015). For all samples with [^210^Pb]_xs_ > 0, the slope of the linear regression of ln[^210^*Pb*]_xs_ on depth is used to calculate the sedimentation rate:

(2) Sed rate(cm yr−1)=−activity ofPb210(0.0311yr−1)∕(slope of the regression)(cm−1)

Excess ^210^Pb profiles for the cores in the present study resulted in very good exponential fits, with correlation coefficients (r^2^) ranged from 0.79 to 0.99, and rates obtained by the CIC model were very similar to those obtained in separate cores collected in 2008 and cores collected and analyzed by Brown University researchers (Boothman et al., 2022; Salacup et al., 2019).

The Constant Rate of Supply (CRS) model (Appleby and Oldfield, 1978) assumes constant flux and no significant migration of ^210^Pb, as with the CIC model, but accommodates varying dilution of ^210^Pb by accelerated sedimentation. For this model, the total inventory of ^210^Pb in the core is calculated:

(3) $$Q^{0}=\Sigma_{i}Q_{i}\;\text{over all}\;i\;\text{intervals},$$

where Q_i_ = ρ_i_ [^210^Pb]_i_ D_i_ for all *i* depth horizons, ρ_i_ = estimated density of sediment layer *i* based on water content of the sediment and an assumed solid phase density of 2.25 g cm^−3^, and D_i_ = thickness of layer *i.* The age of each horizon is then calculated:

(4) Agei(y)=ln(Q0∕ΣiQi)∕Activity(Pb210).

Sediment ages calculated using the CRS model were consistent with those calculated using the CIC model (Table 2), suggesting that there were few perturbations or variations in sediment accumulation at the monitoring sites. In cores where surface sediments appeared to be mixed (BR, CP and SR), higher water content gave rise to lesser inventories of ^210^Pb in the surface horizons, which resulted in slightly younger ages than calculated for the bulk sediment for those horizons. Because of the variation in water content in the near surface sediments, the CRS model was used to calculate ages / dates of deposition for this work. The data and calculations used to date the sediments are included in Table S1 of the supplementary material accompanying this manuscript.

### 2.4. Metals analyses

Concentrations of Mo and Al, along with 7 other metals, were determined in the sediment samples by total digestion and ICP-MS (Mo) or ICP-AES (Al and other metals) analysis. Aliquots of each sample were microwave-digested with a mixture of nitric, hydrofluoric and hydrochloric acids as previously described (Boothman and Coiro, 2009), followed by instrumental analysis of Mo (ICP-MS) and Al and other metals (ICP-AES). Each batch of 24 samples included a procedural blank, one sample analyzed in triplicate, and a certified reference sediment (Marine Sediment MESS-3, National Research Council of Canada). Analytical runs included duplicate analysis of one sample, a spiked blank and a spiked sample for every 20 samples. Relative percent differences of duplicate measurements ranged from 2 to 34 % for Mo and 6–14 % for Al analyses, with spike recoveries from both blanks and samples of 95 to 105 % and 96 to 110 % for Mo and Al, respectively. Overall analytical precision and accuracy were confirmed by the relative standard deviations of triplicate subsample analyses (range 0.3–7.3 %, median 3.1 % for Mo; 0.6–13.1 %, median 4.8 % for Al) and analyte recoveries from the reference sediment MESS-3 (Mo: 84–113 %, median 95 %; Al: 70–121 %, median 86 %). Quality control figures of merit for all analytes are given in Table S2 of the supplementary data accompanying this article.

### 2.5. Carbon and nitrogen analyses

Concentrations and isotopic ratios of carbon and nitrogen in the sediments were determined with a MicroVario Elemental Analyzer (Elementar Americas, Mt. Laurel, NJ) interfaced with an Isoprime 100 Isotope Ratio Mass Spectrometer, following the methods of Oczkowski et al. (2016). The isotope compositions of the samples (δ^13^C and δ^15^N) were reported in the delta notation as the per mil (i.e., part per thousand, ‰) difference between the isotopic ratios in the sample and those in internationally recognized standards - PeeDee belemnite (PDB) for carbon analyses (Paul et al., 2007) and atmospheric N_2_ for nitrogen analyses:

(5) $$\delta X=[(R_{\text{sample}}-R_{\text{standard}}∕R_{\text{standard}}]\times 10^{3},$$

where X is ^13^C or ^15^N, and R is the ratio of the heavy to light isotope (^15^N:^14^N, ^13^C:^12^C). Precision of the measurements, as given by the relative standard deviations of replicate samples, averaged 1.4–2.9 % for both the concentration and isotopic abundance measurements, ranging from 0.1 to 9.7 %. The isotopic composition of the Standard Reference Material BCSS-1 (marine sediment, δ^15^N = 4.66 ± 0.41, δ^13^C = −23.25 ± 0.31) was routinely measured to assure consistency with the accepted international scale. The accuracy of the elemental concentrations in the standard reference material gave a median recovery of 104 %, ranging from 86 to 125 %; the isotopic abundance measurements had a median error of 0.07 ‰, ranging from 0.01 to 0.19 ‰, for δ^13^C and 0.10 ‰, range 0.00–0.65 ‰, for δ^15^N. A complete table of the precision and accuracy for these analyses is included in Table S3 of the supplementary data accompanying this article.

## 3. Results and discussion

### 3.1. Chemical measurements in sediment cores

Sediment cores have been used to document the historical inputs of a multitude of substances, including metals, variety of organic contaminants including petroleum hydrocarbons, aliphatic and polycyclic aromatic hydrocarbons (PAHs), polychlorinated biphenyls (PCBs), chlordanes, linear alkyl benzenes (LABs), benzotriazoles (BZTs) and dichlorodiphenyltrichloroethanes (DDTs), substituted benzotriazoles and phthalic acid esters into Narragansett Bay (Bricker, 1993; Cantwell et al., 2015; Corbin, 1989; Goldberg et al., 1977; Hartmann et al., 2005; Hurtt and Quinn, 1979; King et al., 2008; Pruell and Quinn, 1985; Santschi et al., 1984).

#### 3.1.1. Anthropogenic metals

The profiles of total concentrations of major/minor and trace elements (Al, Fe, Mn, Cu, Zn, Cr, Ni, Mo) are provided in the supplementary material. With the exception of Al, Fe, and Mn, there are very low or non-detectable concentrations in deeper / older sediments (generally prior to the 20th century), increasing to one or more maxima in the late 20th century, and, for Cu, Zn, Cr, Ni, decreasing in more recent sediments near the surface.

- Concentrations of elements related to crustal weathering (Al, Fe, Mn) are fairly constant throughout the sediment column, with slight decreases of Al and Fe in post-1940 sediments at the Greenwich Bay and Quonset Point sites and Mn essentially constant at all sites, suggesting little variation in sedimentation regimes from the late 19th through the 20th centuries.
- Contaminant element concentrations in the oldest samples of the upper Bay cores do not reach background concentrations. Human activities have polluted the Narragansett Bay environment with metals since at least the early 1800s (Nixon, 1995), while the upper Bay cores extend back only to the late 19th century, so failure to reach background in these cores is not surprising. Profiles of most contaminants in the upper Bay display decreasing concentrations in the latter part of the 20th century, beginning around 1940 to 1975.
- Unlike the upper Bay sites, concentrations of contaminant elements in the oldest sediments of cores from Greenwich Bay and the reference sites reach levels similar to those estimated for background sediments in New Bedford Harbor and surroundings (Latimer et al., 2003). Concentrations of most metals increase in more recent sediments of the Greenwich Bay cores up to ~1980, with slight decreases thereafter.
- Contaminant metal concentrations also increase in the Popasquash Point cores from ~1910–1920 to roughly the middle of the 20th century and subsequently decrease. At the same time, concentrations in the Quonset Point cores are almost constant, varying by 10 % or less and reflecting background concentrations, with slight increases of Zn and Cu in post-1980 sediments.
- While a slight anthropogenic influence is evident in the deepest / oldest sections of most of the cores, the profiles of pollutant metals in the sediment cores reflect the historical increase and subsequent tapering of industrialization during the 20th century in the upper Narragansett Bay and Greenwich Bay systems.

#### 3.1.2. Normalizing molybdenum (Mo) and copper (Cu) concentrations

Authigenic Mo is not delivered directly to sediments as are contaminant metals, but rather as a result of redox conditions in the overlying water column that vary spatially as well as temporally. Accordingly, Mo concentrations can vary spatially and temporally in the sediments of the estuary (Adelson et al., 2001; Gooday et al., 2009; Morford et al., 2009; Zheng et al., 2000). Rather than the total concentration of Mo, duration of hypoxia is related to the authigenic fraction, that is, the metal precipitated or scavenged from interstitial waters or the water column and deposited in sediments (Adelson et al., 2001; Crusius et al., 1996; Crusius and Thomson, 2000; McManus et al., 2006a; Morford and Emerson, 1999; Scholz et al., 2011; Zheng et al., 2000). Authigenic Mo concentrations were calculated by subtracting the lithogenic fraction of Mo - that formed by weathering of crustal material and estimated by multiplying the aluminum (Al) concentration measured in each sample by the mean Mo:Al ratio in continental crust - from the total Mo concentration:

(6) $${\left[\text{Mo}\right]}_{\text{auth}}={\left[\text{Mo}\right]}_{\text{total}}-{\left[\text{Mo}\right]}_{\text{lith}}={\left[\text{Mo}\right]}_{\text{total}}-{\left[\text{Al}\right]}_{\text{total}}\times {\left[\text{Mo}\;:\text{Al}\right]}_{\text{crust}}$$

where [Mo:Al]_crust_ = 1.8226 × 10^−5^ (Taylor, 1964).

There is some uncertainty surrounding the Mo:Al crustal ratio used to calculate authigenic Mo concentrations; the calculations deriving the ratio assume granitic rocks comprise about half the input to the crust (Taylor, 1964), while some evidence indicates that granitic rocks are depleted in Mo due to MoS_2_ precipitation or loss of Mo during geologic processes (Greaney et al., 2018). However, we had no data for the local geology that would cause us to use a ratio different from that of Taylor. Adjusting the Mo:Al crustal ratio by ±10 % changed authigenic Mo concentrations by <10 % for 90 % of the samples and < 15 % for 95 % of the samples, so we used Taylor’s ratio to estimate the lithogenic fraction of Mo. Some Mo may also be deposited in sediments in association with organic matter but, with organic carbon contents of 1.3–4.9 % in the sediments involved with this work, that fraction is likely very small.

Similarly, concentrations of non-lithogenic copper can be used as a marker of anthropogenic influences (Adelson et al., 2001). The non-lithogenic fraction of Cu in sediments was determined in the same manner as authigenic Mo, that is, by calculating the lithogenic fraction as the product of the measured aluminum (Al) concentration by the Cu: Al ratio in continental crust and subtracting that from the total Cu concentration:

(7) $${\left[\text{Cu}\right]}_{\text{ex}}={\left[\text{Cu}\right]}_{\text{total}}-{\left[\text{Al}\right]}_{\text{total}}\times {\left[\text{Cu}\;:\text{Al}\right]}_{\text{crust}}$$

where [Cu:Al]_crust_ = 6.683 × 10^−4^ (Taylor, 1964).

#### 3.1.3. Carbon and nitrogen analyses

Concentrations of carbon and nitrogen and their isotope ratios in sediment have often been used to make inferences about eutrophication within the Narragansett Bay ecosystem (King et al., 2008; Oczkowski et al., 2016; Oczkowski et al., 2010). Increasing concentrations of C and N can indicate increased delivery of organic matter in sediments and/or post-depositional diagenesis. Stable isotope measurements of carbon (δ^13^C) are often used as indicators of sources of organic matter in coastal sediments, as values in marine phytoplankton (typically–21 to −23 ‰) differ from those in salt marsh grasses (−12 to −14 ‰) and in upland plants (~ −28 ‰). Stable isotope measurements of nitrogen (δ^15^N) may also be used to assess sources, although interpretation is complex due to multiple sources and overlapping ranges of δ^15^N values. While δ^15^N values in coastal marine plankton are usually ~8.5 ‰, nitrogen in groundwater derived from septic systems, secondary sewage treatment plants (those using biological processes to purify wastewater), or untreated human sewage is typically more enriched (δ^15^N = +10 to +22 ‰) (Schmidt et al., 2016), such that increasing δ^15^N values indicate anthropogenic eutrophication within the study areas (Zimmerman and Canuel, 2002). Similarly, the ratio of carbon to nitrogen (C/N) has been used to infer the sources of organic matter, with lower values (5–13) indicative of marine and higher values (15–45) terrestrial sources (Bordovskiy, 1965; Perdue and Koprivnjak, 2007). Lower C/N ratios may also reflect areas subjected to intensive microbial decomposition of organic matter, as particulate N is added to sediments as detrital biomass (Thornton and McManus, 1994).

### 3.2. Geochemical records of hypoxia in Narragansett Bay

#### 3.2.1. Upper Bay (BR, CP, NP)

**Fig. 2.**
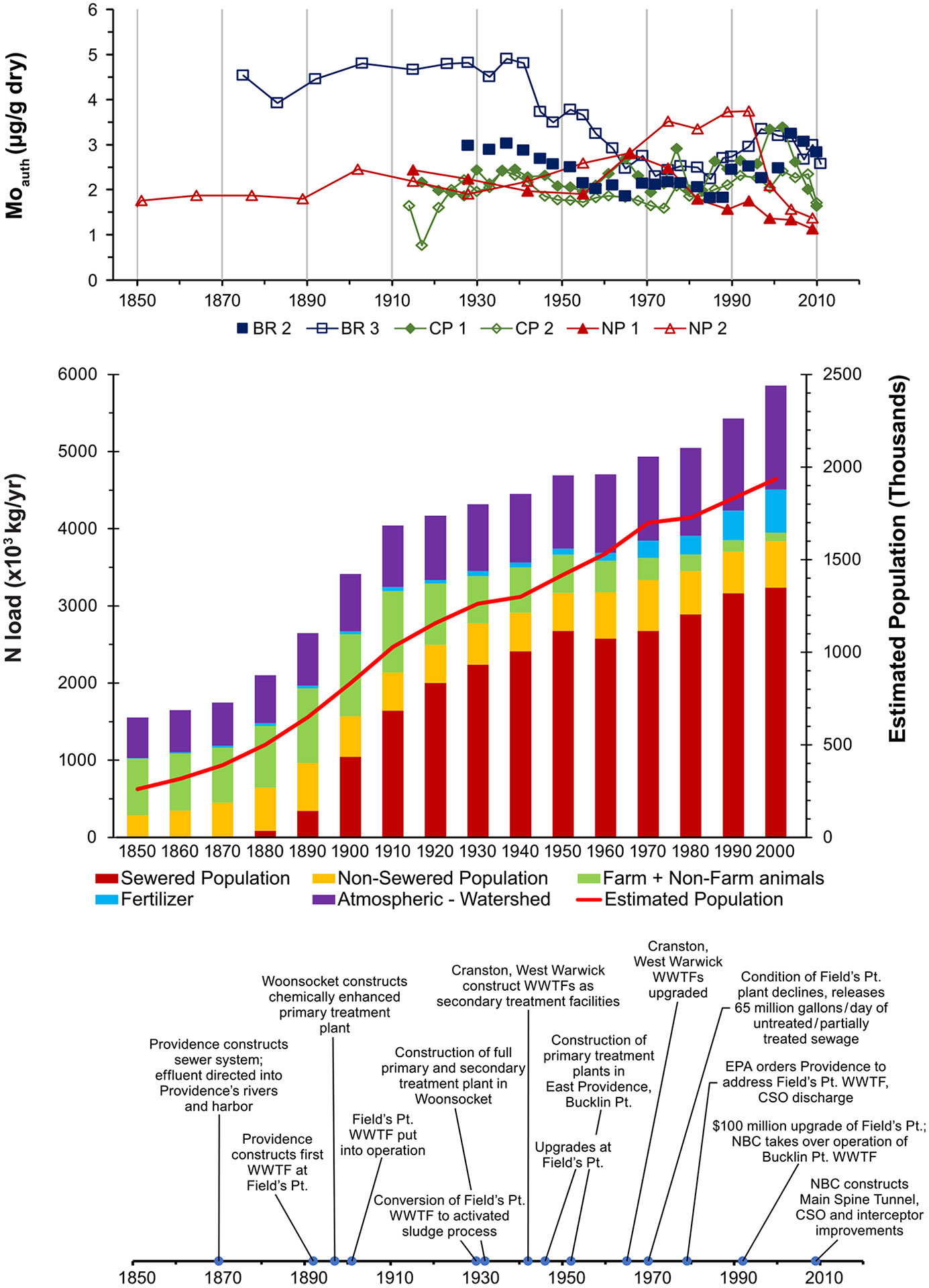
Timelines of a) authigenic Mo concentrations, b) estimated population and nitrogen loading, and c) development of WWTFs in Upper Narragansett Bay sediments. Nitrogen load and population estimates from Vadeboncoeur et al. 2010.

Mo concentrations in Narragansett Bay sediments reflect differing patterns of hypoxia, likely resulting from differing historical development and population in the watersheds surrounding the waterbodies. The frequency/duration of hypoxia in upper Narragansett Bay (Fig. 2), as indicated by concentrations of authigenic Mo in sediments, reflects improvements and degradations of WWTFs and combined sewer overflows (CSOs), the dominant sources of nitrogen there. In contrast to profiles of anthropogenic contaminants, Mo concentrations in sediments from the late 19th and early 20th centuries at the Bullocks Reach (BR) site within the Providence River were approximately double those of more recent times, indicating more frequent hypoxia through most of the first half of the 20th century. Most domestic and industrial wastewater went directly into rivers in the late 19th century; even as the City of Providence first implemented sewers in 1870, the collected effluent still was directed into the Providence River and tributaries. In 1897, the city of Woonsocket constructed a chemically enhanced primary treatment plant where solids were removed from wastewater emptying into a tributary of the Providence River. Providence followed suit with its first facility at Field’s Point in 1892, with chemical precipitation implemented in 1901 (Narragansett Bay Commision, 2018; RI Department of Environmental Management, 2016). These developments do not seem to have reduced the frequency and duration of hypoxia in the Providence River, as Mo concentrations in sediments from this time period remained elevated. It is likely that effluent initially discharged at Fields Point was essentially untreated sewage (Nixon et al., 2008) and the “treatment” did little to reduce the loading of nutrients and organic matter to the river waters.

Mo concentrations in Providence River sediments declined beginning around 1940, indicating reduced duration of hypoxia following conversion of the Field’s Point plant to an activated sludge process and construction of a full primary and secondary treatment plant in Woonsocket in the early 1930s. Installation of new equipment and upgrades at the Field’s Point plant between 1946 and 1950 and construction of WWTFs in East Providence in 1952 and 1954 (RI Department of Environmental Management, 2016)reducing the nitrogen load to the Providence River. WWTFs constructed in Cranston and West Warwick in 1942 and upgraded during the late 1960s further reduced loading to the Pawtuxet River that empties into the Providence River. Reflecting these improvements, Mo concentrations in sediments indicate steadily decreasing hypoxia in the Providence River into the mid-1960s to 1970. The city of Warwick also constructed a WWTF in 1965, but its effect may have been to divert wastewater nitrogen as effluent into the Pawtuxet River and thereby to the Providence River / upper Bay that would have been delivered via groundwater to Greenwich Bay.

In contrast to the improvements of the 1940s through the 1960s, Mo concentrations increased in sediments from the 1970s, reflecting increased hypoxia, in response to millions of gallons of untreated or partially treated sewage flowing into the Providence River daily due to the declined condition of the Field’s Point plant(Narragansett Bay Commision, 2018), although the increase may have been moderated by construction of new secondary treatment facilities in West Warwick (1973), Woonsocket (1975), and Smithfield (1978) and upgrades to secondary treatment at Bucklin Point (1972) and East Providence (1976) (RI Department of Environmental Management, 2016), compensating for the increased nitrogen load from Fields Point. In 1980, the Narragansett Bay Water Quality District Commission formed and an $87.7 million bond was passed to fund improvements at Field’s Point (Narragansett Bay Commision, 2018), while Cranston constructed a new secondary treatment plant in 1982 and major upgrades were performed at Bucklin Point in 1985 and 1989 (RI Department of Environmental Management, 2016), and again, Mo concentrations in Providence River sediments decreased from ~1975 to 1990, reflecting decreasing periods of hypoxia. Despite the improvements of the 1980s, Mo concentrations in the Providence River sediments increased through the 1990s, indicating increasing durations of hypoxia. However, following efforts to close combined sewer overflows (CSOs), convert the largest WWTFs from secondary to tertiary treatment, and construct the Main Spine Tunnel, Mo concentrations in BR sediments steadily decreased from ~2000 through 2010.

This same trend was not seen for the other upper Bay sites - Conimicut Point (CP) and North Prudence (NP) - located outside the Providence River, where Mo concentrations were lower and relatively constant from the early 20th century up to ~1970 before increasing through 1980 to 1990 and decreasing again after 2000. Despite the geographical proximity, hypoxia appears to have been less frequent in the early 20th century at the CP and NP sites, with Mo concentrations similar to the lowest found in the BR cores, possibly due to retention of hypoxic bottom water within the Providence River because of the narrowing and shallow depths at its mouth. A similar disparity was observed in the late 1980s, with comparable dissolved metals concentrations on either side of the sill, but much higher concentrations of particulate metals in the Providence River (Bender et al., 1989). On the other hand, more recent historical trends in cores from both sites are similar to those in the Providence River, with increasing Mo concentrations from ~1980 to ~2000, and a subsequent decrease through 2011.

**Fig. 3.**
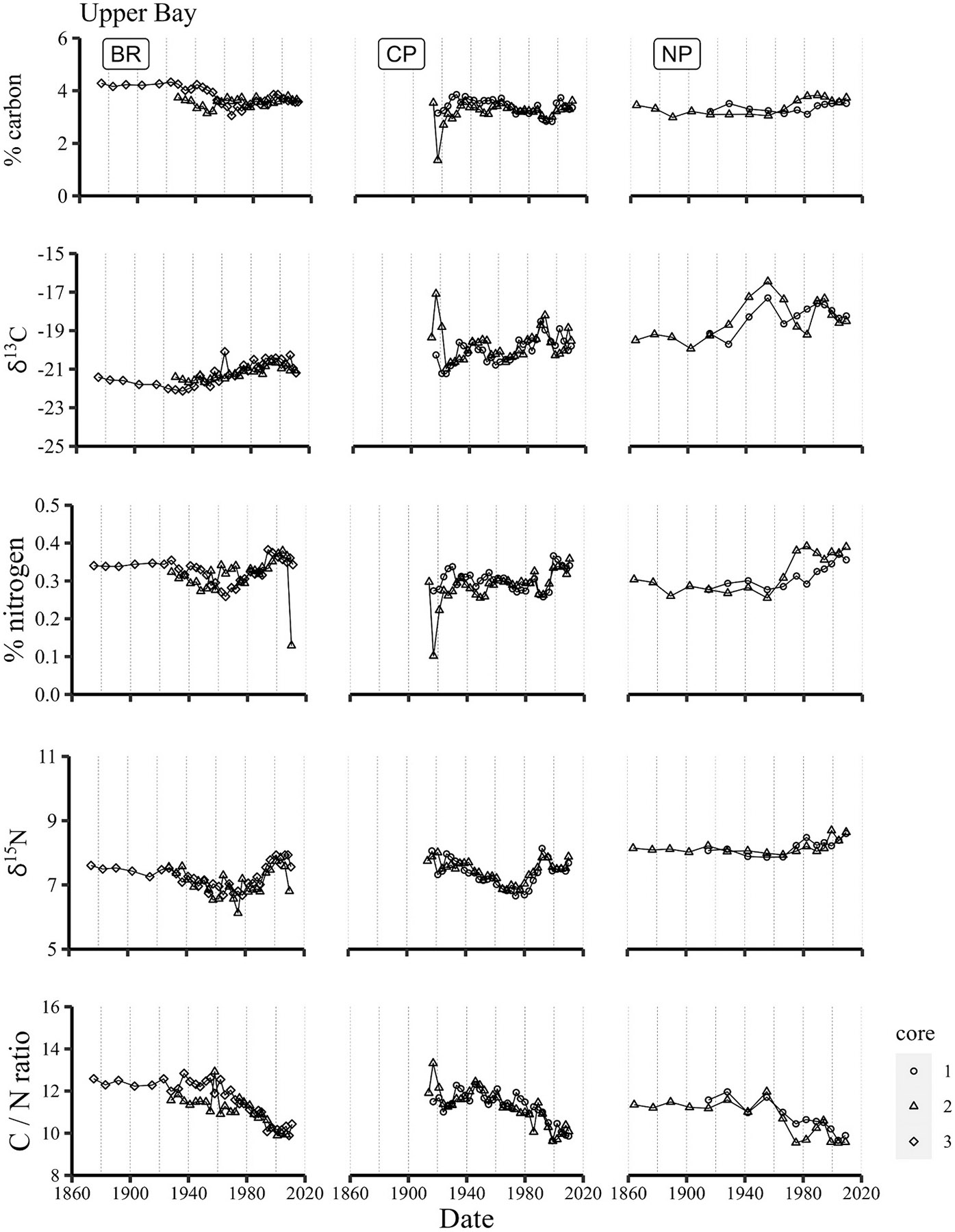
Timelines of carbon and nitrogen concentrations, isotopic ratios and C/N ratios in Upper Narragansett Bay sediments.

Despite substantial variations of sewage inputs to the Providence River, little variation in concentrations and isotope values of carbon and nitrogen is noted in upper Narragansett Bay sediments through the 20th century, and likewise little difference between sites within (Bullock’s Reach) and outside of the Providence River (Conimicut Point and North Prudence) (Fig. 3). Carbon concentrations at Bullock’s Reach were slightly higher (~4.5 %) in the late 19th / early 20th century and decreased to 3–4 % between 1940 and 1960, similar to those at Conimicut Point (CP) and North Prudence (NP). δ^13^C values increased slightly through the second half of the 20th century, but still indicate marine phytoplankton to be the major source of carbon. This is not surprising, as primary production is estimated to be four-fold greater than other sources of organic carbon to Narragansett Bay (Nixon et al., 1995). δ^13^C values at North Prudence increased between ~1930 and 1970, suggesting a period of more intense productivity, but this was not reflected in carbon concentrations from that same time period nor observed at nearby sites. An increase in nitrogen concentrations and shift to isotopically heavier nitrogen from ~1970 through 2000 may reflect increasing influence of sewage-derived nitrogen.

#### 3.2.2. Greenwich Bay (GB, SR)

**Fig. 4.**
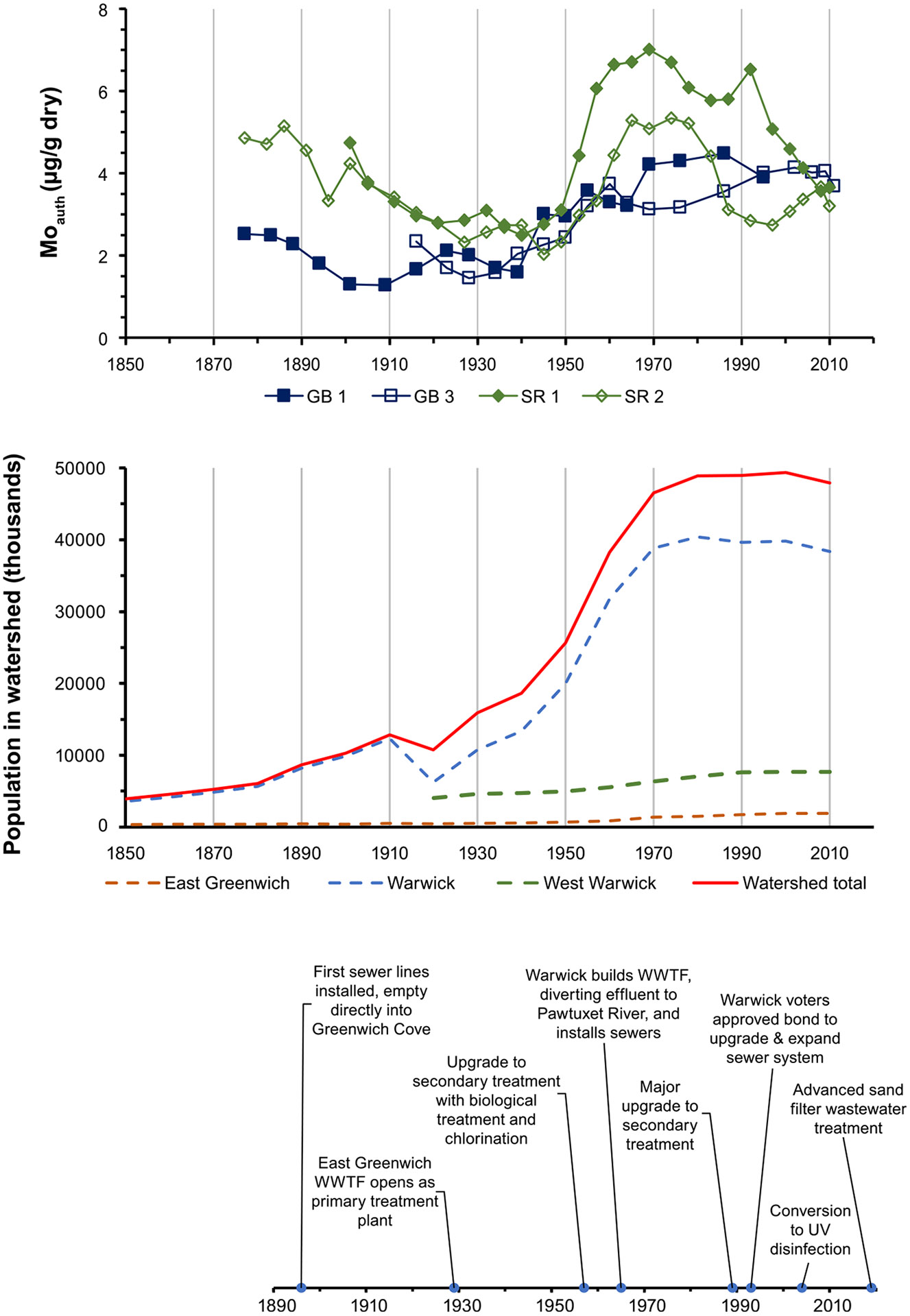
Timelines of authigenic Mo concentrations in sediments and estimated population in the Greenwich Bay watershed. Population estimates from Pesch et al. (2012).

As with other coastal cities and towns in New England, the towns in the Greenwich Bay watershed experienced periods of industrial growth and subsequent decline, tied to first maritime and then textile and other manufacturing industries, particularly during the 19th into the early 20th century, albeit on a smaller scale (Pesch et al., 2012; Voyer et al., 2000). The impact of these industries through the 19th century was relatively small, however; concentrations of contaminant metals such as copper, lead, and chromium in sediments at the base of the GB and SR cores, corresponding to the early 20th century (supplemental data), were similar to those found in background sediments in New Bedford, MA (Latimer et al., 2003) and the Providence River (Corbin, 1989). One lasting effect of the 19th century industrialization around Greenwich Bay was the growth in population, as workers moved into the area. Over the course of the 19th century, the population in the watershed increased almost 5 times (Pesch et al., 2012), contributing to increased amounts of wastewater flowing via groundwater from cesspools or sewers installed in East Greenwich in the late 1890s that emptied directly into Greenwich Cove. This increased input of nitrogen appears to have resulted in substantial periods of hypoxia, as authigenic Mo concentrations in the deeper sediments of the GB and SR cores (~1880–1920) were elevated relative to the concentrations found in the Upper Bay at the same time and approximately double the minimum concentrations that occurred in the early 20th century (Fig. 4). When the first WWTF in East Greenwich was constructed in 1928, it was strictly primary treatment and the effluent still emptied into Greenwich Cove (Pesch et al., 2012), yet even that appeared to have helped reduce the frequency of hypoxia into the next decade.

**Fig. 5.**
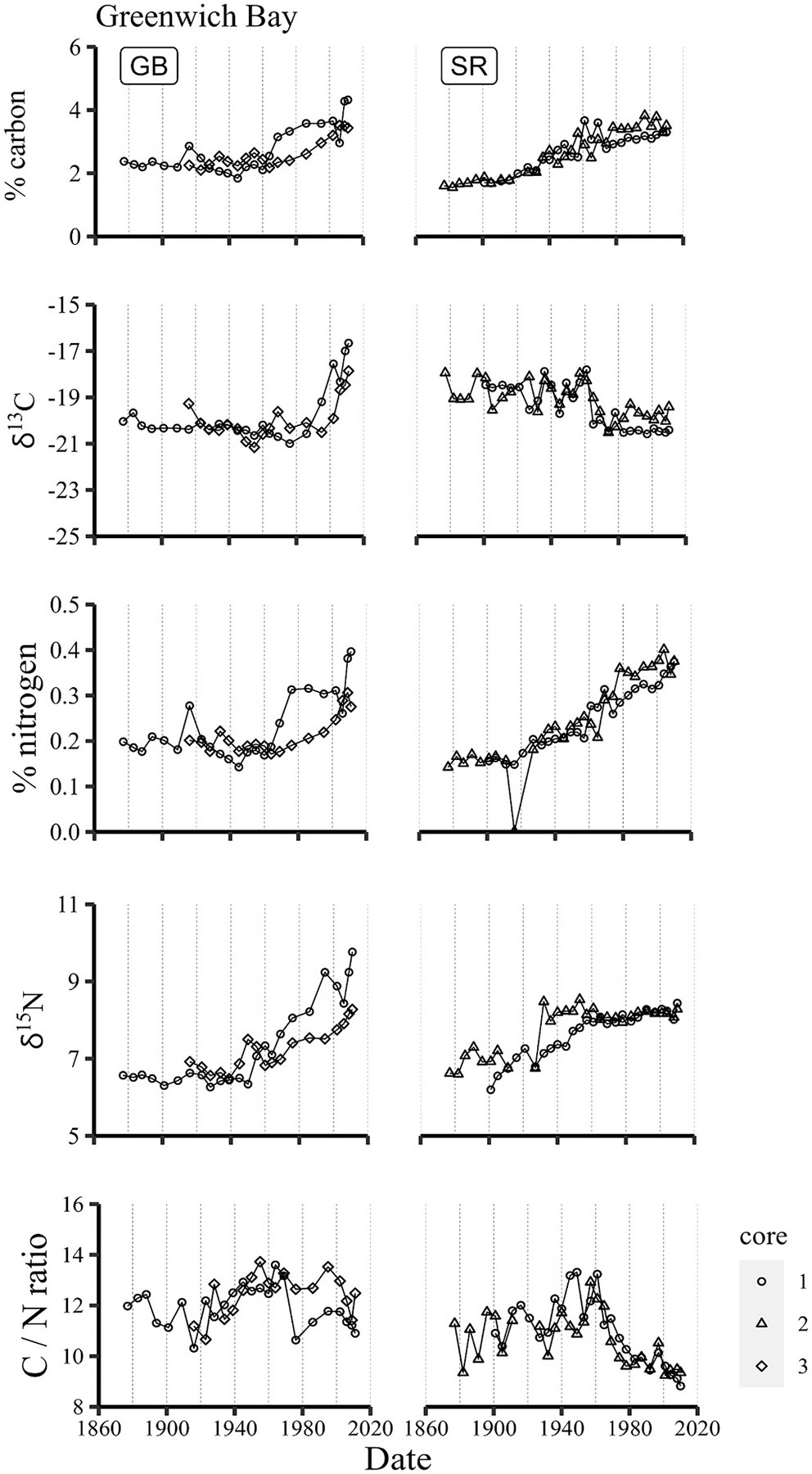
Timelines of carbon and nitrogen concentrations, isotopic ratios and C/N ratios in Greenwich Bay sediments.

Continuing the trend from the beginning of the 20th century, population within the Greenwich Bay watershed increased rapidly from ~1940 through the mid- to late-1970s, before levelling off from the 1980s through 2010, with the estimated population in the watershed almost doubling again between 1950 and 2000 (Pesch et al., 2012). Accompanying this growth in population was substantial conversion of agricultural land to residential properties, altering the delivery and quantity of nitrogen to the groundwater from fertilizers to residential wastewater. This change was evident in sediments in Greenwich Bay, where nitrogen isotope ratios (δ^15^N) increased from 1940 onward, reflecting the impact of sewage-derived nitrogen (Fig. 5). Concentrations of carbon and nitrogen increased ~2–3 fold from early 20th century levels, beginning around 1920 at Sally Rock (SR) and 1960–1970 at Greenwich Bay (GB). Carbon isotope ratios (δ^13^C) through the first half of the 20th century were constant around −20 ‰ at GB and higher and more variable at SR, indicating the carbon to be primarily marine in origin. In the mid- to late-1970s through the present, δ^13^C at the GB site shifted rapidly to ~ −17 ‰, possibly indicative of intense phytoplankton blooms (Oczkowski et al., 2010), although a similar shift was not seen at the SR site, where the ratio shifted slightly lower.

Authigenic Mo concentrations in sediments from the two Greenwich Bay sites during this period of growth indicate a rapid and substantial increase in the duration of hypoxia from 1940 to 1970, reflecting the post-World War II suburbanization of the surrounding watershed (Fig. 4). Mo concentrations continued to increase at GB into the early 21st century before decreasing slightly after ~2000, whereas Mo concentrations increased more rapidly and to higher concentrations after 1940 in cores from SR along the southern shore of Greenwich Bay, peaking in the late 1970s and 80s before decreasing from 1980 through the turn of the century.

This spatial difference may be related to the circulation patterns within Greenwich Bay. Tidal flow and dominant southerly winds in summer months create counterclockwise gyres in the western and eastern basins of Greenwich Bay, which can increase residence time of waters in Greenwich Bay from 9 days to 1 month, particularly in the western basin (Balt, 2014; Codiga et al., 2009; Rogers, 2008). The GB site is located on the western side of the western basin, and thus is subject to these longer residence times, resulting in persistent hypoxia annually (≥ 30 d yr^−1^) (Boothman et al., 2022).

The SR site, on the other hand, is located along the southern shore of Greenwich Bay between the western and eastern basins. In addition to waters from the western gyre with longer residence times, it is exposed to waters from the eastern basin gyre with shorter residence times and higher dissolved oxygen concentrations. The City of Warwick constructed a WWTF with secondary treatment in 1965, but the initial sewer lines were all outside the Greenwich Bay watershed (Pesch et al., 2012) and subsequent connections were slow in coming - even today, approximately 30 % of the developed parcels in Warwick are not sewered (Warwick Sewer Authority, 2022). Because the WWTF effluent empties into the Pawtuxet River, diversion of septic wastewater into sewers would have decreased nitrogen concentrations in groundwater subsequently entering Greenwich Bay. With horizontal groundwater transport rates of 0.1 to 0.5 m d^−1^ (Addy et al., 2005; Nowicki and Gold, 2008), however, plumes of lower nitrogen wastewater may not have reached Greenwich Bay for years to decades, delaying evidence of the decrease. Consequently, sediments at the SR site may have experienced hypoxic conditions related to rapid population growth in the watershed, but also decreased periods of hypoxia resulting from decreased nitrogen loading to Greenwich Bay from the 1970s onward.

#### 3.2.3. Reference sites (PP, QP)

**Fig. 6.**
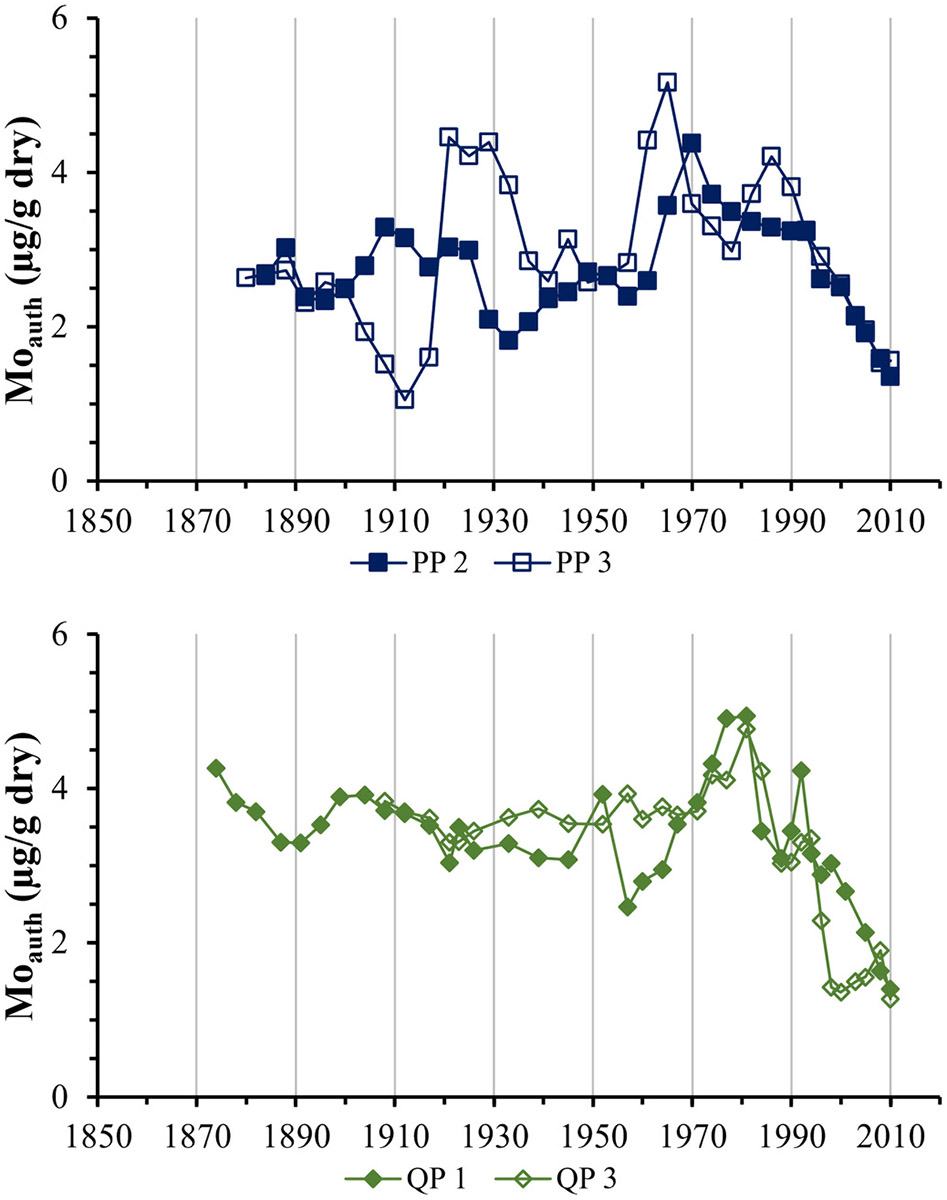
Timelines of authigenic Mo concentrations in sediments at the reference core sites.

The sites designated in this work as reference sites were selected to represent conditions in Bay waters being delivered by estuarine circulation into (Popasquash Point, PP) and out of (Quonset Point, QP) the Upper Bay study areas. Because of their location within the East and West Passages rather than embayments, these sites were thought to be removed from localized sources of nitrogen loading and not subject to many of the forces that typically drive the formation of hypoxic condition, and thus not expected to contain significant concentrations of authigenic Mo. This proved not to be true at either site, as deeper sediments from both sites contain elevated and variable historical concentrations of authigenic Mo (Fig. 6). Concentrations in sediments at both sites deposited prior to ~1960 were about 50 % higher than the apparent background concentration seen in the upper Bay sites CP and NP. Concentrations in these older sediments were consistent in the QP cores, ranging from ~1.5 to 3 μg/g, but much more variable in the Popasquash Point sediments, particularly in one core where concentrations varied from ~1.5 to as low as 0.5 and as high as 3.6 μg/g within in a period of ~40 years.

Profiles of authigenic Mo were more consistent both within and between reference sites in sediments deposited after 1960, rising to maxima between 1970 and 1980 and decreasing rapidly thereafter, much like in the upper bay cores. Maximum concentrations occurred in the PP sediments around 1970, decreasing rapidly thereafter to apparent background concentrations, with one core exhibiting a brief spike in concentration around 1985. The QP cores were similar, albeit slightly more temporally condensed: maximum concentrations occurred ~1980, again with a small spike ~1992 in one core. A similar peak in Mo concentrations in the same time period is evident in several of the upper Bay cores – both BR cores, core 1 from CP and core 2 from NP – as well as one of the SR cores. The similarity seems to suggest a regional process influencing the formation of hypoxia, though a cause for such widespread occurrence of hypoxia is not immediately apparent.

The reason for elevated concentrations of authigenic Mo in the deeper sediments at PP and QP is unclear. Similar trends of increasing Mo concentrations with depth have been seen in marine sediments elsewhere (Chaillou et al., 2002; Malcolm, 1985), but are not seen in multiple sediment core profiles from Chesapeake Bay, an estuary often compared to Narragansett Bay in ecological studies (Adelson et al., 2001; Helz and Adelson, 2013; Scheiderich et al., 2010; Zheng et al., 2003). One possible cause might be slow kinetics of Mo fixation. Sundby et al. (2004) found Mo concentrations increasing with depth below a subsurface minimum through the entire length (40–45 cm) of sediment cores from the Gulf of St. Lawrence and attributed the accumulation at depth to slow rates of Mo fixation compared to diffusive transport, enabling Mo in interstitial waters to diffuse downward and accumulate authigenic phases below the depth where precipitation initiates. The initial form of Mo immobilized in sulfidic sediments (Crusius and Thomson, 2000; Dean et al., 1999; Helz et al., 1996; McManus et al., 2006b; Zheng et al., 2000) may be particle reactive thiomolybdates (${{\text{MoS}}_{x}O}_{\left(4\text{-}x\right)}^{2-}$) produced by reaction of dissolved molybdate (${\text{MoO}}_{4}^{2-}$) with sulfide. Helz et al. (1996) proposed the existence of a sulfide “switch” whereby, equilibrium between molybdate and thiomolybdates shifts abruptly to thiomolybdates with gradually increasing sulfide concentrations: in the presence of reactive iron, co-precipitation of a Mo-Fe-S phase began at an apparent threshold of 0.1 μM S^2−^, with a second threshold of 100 μmol/L S^2−^ for Mo precipitation without Fe. Sedimentation rates at PP and QP (0.35 and 0.43 cm yr^−1^, respectively) were higher compared to the other sites (0.14–0.28 cm yr^−1^) (Boothman et al., 2022), and with relatively low carbon concentrations (≤ 2 %) in deeper sediments at the reference sites compared to more recent sediments, diagenetic sulfide production may also have been lower, resulting in rates of Mo accumulation slow enough to allow dissolved Mo to diffuse to deeper sediments before precipitating. In addition, Morford et al. (2009) found that sediment irrigation could deliver bottom water to deeper sediments where reaction with sulfide could immobilize dissolved Mo, but also could remobilize previously precipitated Mo, enabling it to be physically or diffusively transported into deeper sediments. This same mechanism might apply to elevated Mo concentrations in deeper sediments at the Greenwich Bay stations and perhaps the BR station, but the higher carbon concentrations at BR make this process less likely there.

These sites are not truly reference sites, in the sense of being free from influences of anthropogenic activities. The PP site is outside the mouth of Bristol Harbor, the receiving waters for the Bristol WWTF. The QP site, while remote from the urban cities in Rhode Island, is located offshore from a former Naval Air Station, now home to an industrial park and automobile delivery port, with a WWTF constructed in 1941 as a primary treatment plant and upgraded in 1992 to provide secondary treatment. Both the Bristol and Quonset WWTFs are relatively small compared to the systems affecting upper Narragansett Bay and not expected to contribute to the formation of hypoxic conditions. But the PP site is also not far from the mouth of Mount Hope Bay, which receives effluent from the Fall River WWTF, the second largest source of nitrogen to Narragansett Bay from direct discharge of sewage treatment plants (Nixon et al., 1995). It is possible that estuarine circulation could have entrained water exiting Mount Hope Bay and transported its nutrients to the PP site. And from 1908 to 1949, the city of Providence dumped sewage sludge containing some 137,400 tons of dry solids in an area southeast of the QP site (Nixon, 1995), which potentially contributed organic carbon and nitrogen to West Passage waters.

**Fig. 7.**
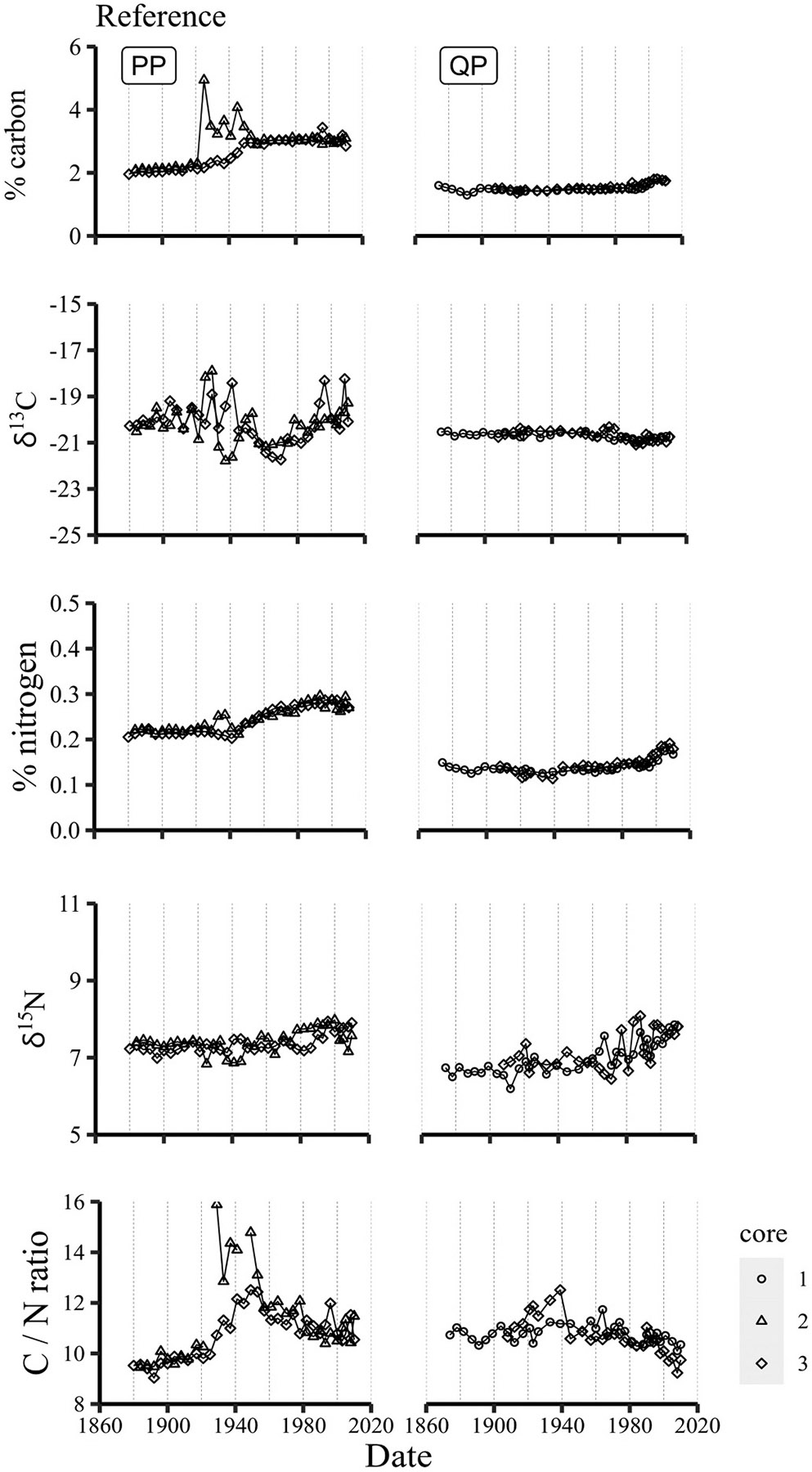
Timelines of carbon and nitrogen concentrations, isotopic ratios and C/ N ratios in reference site sediments.

The carbon and nitrogen concentrations and isotope ratios in sediments from these sites, however, do not indicate of any major changes in sources or productivity over time, particularly in the latter half of the 20th century (Fig. 7). These measurements were practically invariant in the QP cores: %C was constant at ~1.5 % from 1880 through 1990, increasing to 1.75 % by 2010; δ^13^C values varied within a narrow range (−21.1 to −20.3 ‰); and N concentrations and isotopic ratios increased only from 0.14 to 0.19 % and 6.9 to 7.8 ‰, respectively, from the mid- to late-1960s to 2010. Concentrations of carbon shifted from ~2 to 3 % between 1920 and 1955 in PP cores and nitrogen from 0.2 to 0.3 % after 1940. In one of the PP cores, carbon concentrations were higher and more variable (3–5 %) between 1920 and 1950, suggesting possible disturbance in its depositional history, but similar variability of nitrogen concentrations was not observed in that core. Carbon isotopic ratios (δ^13^C) were also more erratic in those sediments, ranging from −18 to −22 ‰ in both PP cores during that same timeframe, with values of ~ −20.0 ‰ in earlier sediments and ranging more gradually from −21 to −20 ‰ after 1955. Nitrogen concentrations and isotope ratios, on the other hand, remained essentially constant at 0.2 % and 7.7 ‰ from 1900 through ~1940, then increasing to 0.3 % and 7.9 ‰ by 2010. A complete listing of the C and N concentrations, isotope ratios, and C/N ratios is given in Table S5 in the supplementary data.

### 3.3. Comparison to Chesapeake Bay

The relationships between nutrient enrichment, hypoxia and Mo in sediments have been extensively studied in Chesapeake Bay, a large, partially stratified estuary in the Mid-Atlantic region of the United States, and particularly the mesohaline portion of the Bay between the city of Baltimore to the north and the Potomac River to the south. Distributions of authigenic Mo in this section of the Bay reveal temporal patterns of hypoxia that vary spatially and reflect the influence of both natural and anthropogenic processes (Adelson et al., 2001; Scheiderich et al., 2010; Zheng et al., 2003; Zimmerman and Canuel, 2002). Authigenic Mo concentrations decrease from north to south, following the observed pattern of oxygen depletion. Slight accumulation as early as the 1930s is found in the northernmost cores, but in most cores, there is little accumulation of Mo prior to 1960 and the highest concentrations of authigenic Mo occur in sediments deposited after ~1960. A slow general increase in concentrations from ~1940 to ~2000 reflects increased intensity and frequency of hypoxia (Zheng et al., 2003). By comparison, excess Cu concentrations, indicative of anthropogenic activity, are widespread, peak in the early 20th century, and do not correlate with authigenic Mo concentrations, suggesting that different processes drive the occurrence of hypoxia and accumulation of anthropogenic Cu (Adelson et al., 2001). In cores from the southern mesohaline part of the Bay, peaks of authigenic Mo correspond with periods of greater wet deposition, indicating freshwater discharge to the Bay as a driving factor for seasonal hypoxia (Cronin and Vann, 2003; Zheng et al., 2003). Taken as a whole, the core profiles suggest that natural factors such as spring runoff and nutrients influence the development of hypoxia throughout the Bay, while “cultural eutrophication” intensifies hypoxia in northern Chesapeake Bay.

The profiles of authigenic Mo in Narragansett Bay bear some similarities to those in Chesapeake Bay, but also several differences, particularly in the timing of increased authigenic Mo concentrations. The highest concentrations of authigenic Mo occur in sediments deposited after ~1960, with peak concentrations occurring in the late 20th century. Unlike the Chesapeake Bay cores, however, elevated concentrations of authigenic Mo were found in late 19th and early 20th century sediments from most of the Narragansett Bay sites. Only in the upper Narragansett Bay cores outside the Providence River (CP and NP) did Mo_auth_ concentrations increase beginning in the mid-1930s as in Chesapeake Bay. Likewise, the slow general increases in concentrations from ~1940 to ~2000 and from south to north in the Chesapeake were not found in Narragansett Bay sediments.

**Fig. 8.**
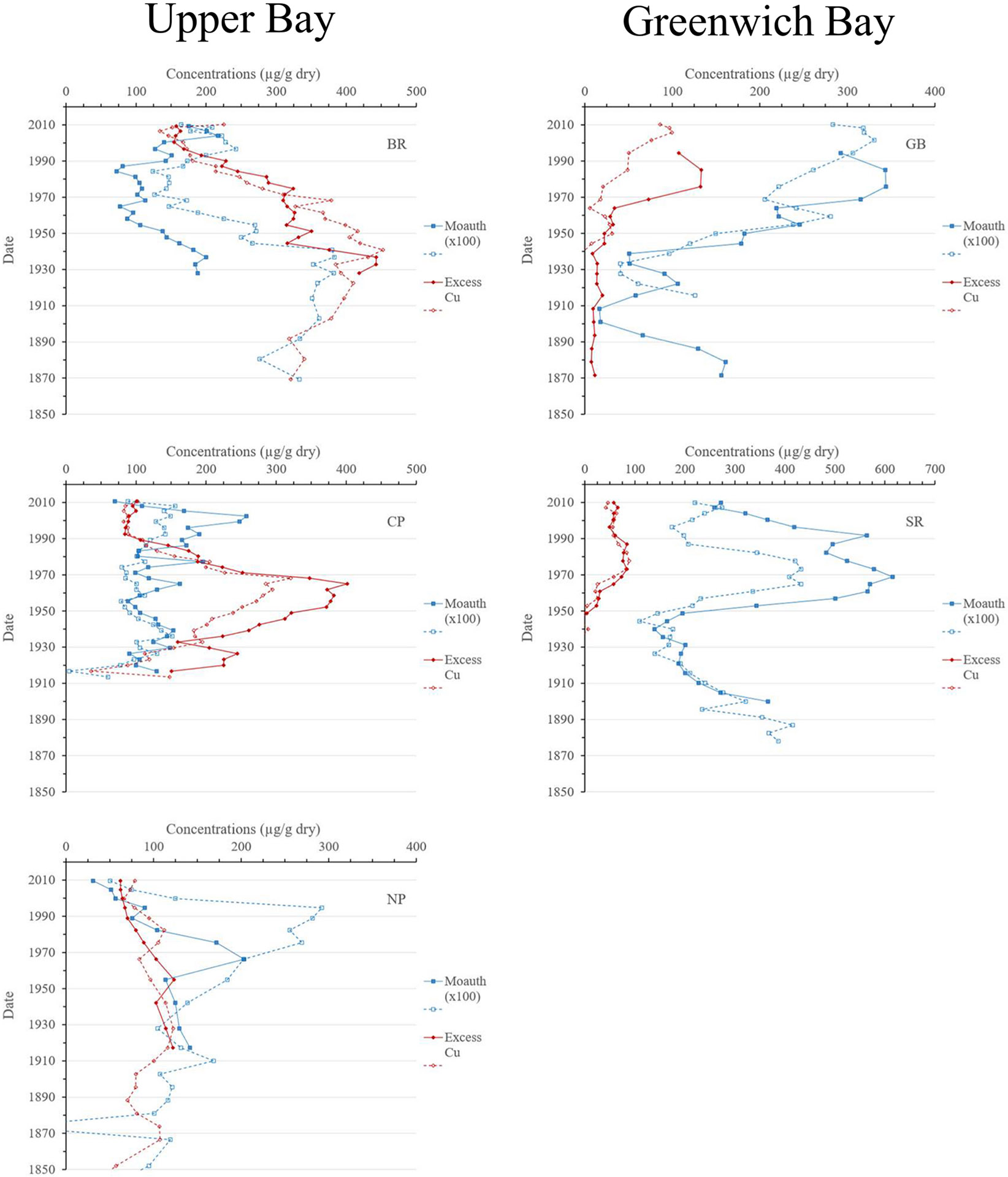
Profiles of authigenic Mo and excess Cu in Upper Narragansett and Greenwich Bay sediments. Note change of concentration axis for site SR.

One significant difference between the Chesapeake and Narragansett Bays is the correlation between concentrations of “excess copper” (Adelson et al., 2001) and authigenic Mo concentrations. While not significant through entire cores, such correlations were evident in portions of some of the Narragansett Bay cores (Fig. 8). In upper Narragansett Bay, sediment concentrations of excess Cu and Mo_auth_ at the BR site decreased consistently from ~1935–40 through ~1965, and a smaller peak in Mo_auth_ concentrations in the early 1960s at the CP site corresponded with peak Cu concentrations. Similarly, peak concentrations of excess Cu and Mo_auth_ coincided in the Greenwich Bay, implying a stronger connection between anthropogenic / industrial activity and the development of hypoxia in Narragansett Bay than in the Chesapeake.

Much of differences between Chesapeake and Narragansett Bays may be attributed to the relative geographic scale of the Bays. Chesapeake Bay is roughly twice as wide and >7× as long as Narragansett Bay, and the samples for the studies of Chesapeake Bay were taken from the central channel of the Bay, several km from shore and anthropogenic point sources of nutrients, whereas the Narragansett Bay sites were all within 1–2 km of shore and adjacent to WWTFs and industrial/population centers. In addition, the rivers supplying freshwater to Narragansett Bay pass through several cities and industrial areas before reaching the head of the Bay, whereas the headwaters of Chesapeake Bay derive from a substantially rural / agricultural watershed. Consequently, the Narragansett Bay samples are more likely to reflect the impacts of localized point sources and shoreline activities rather than more generalized anthropogenic factors (e.g., atmospheric deposition, increased population, changing land use in the Chesapeake watershed) affecting the Chesapeake samples.

## 4. Conclusions

Even as considerable interest and research efforts are examining the ecological effects resulting from substantial reductions in the nitrogen load being delivered to Narragansett Bay, the historical record of hypoxia in the Bay’s waters is incomplete. Detailed spatial records of dissolved oxygen measurements at sites in Narragansett Bay have only been available since 2004 (Narragansett Bay Fixed-Site Monitoring Network, 2004–2011). The redox chemistry of Mo in surficial sediments provides sedimentary records that enable reconstruction of the historical prevalence of hypoxia within the Bay and reflect the influence of both local and regional activities, allowing hind casting of the effects of changing sources of nitrogen in the watershed. In Narragansett Bay, an estuary whose sources of reactive nitrogen are dominated by wastewater from a number of sources, concentrations of Mo in sediments vary in conjunction with changes in the surrounding watershed that affect the amount of nitrogen loading in the Bay’s waters, reflecting decreased hypoxia in response to implementation of and improvements to wastewater treatment systems and increased hypoxia following deteriorating WWTF operations or rapidly increasing populations. The sediment records also reveal differences in the spatial extent of hypoxia: significant periods of hypoxia within the Providence River, but little in upper Narragansett Bay in the early 20th century, and hypoxia rapidly decreasing in southern / outer parts of Greenwich Bay while concurrently increasing in western Greenwich Bay. The records also reveal an apparent regional trend of continually increasing hypoxia from the late 1970s into the late 1990s, and recent sediments suggest decreased hypoxia throughout the Upper Bay following CSO improvements and completion of the Combined Sewer Overflow Tunnel in 2008.

Mo sediment records also illustrate the different historical development in Greenwich Bay, with historical sources of nitrogen there derived more from groundwater containing septic effluents and less from WWTF effluent. Consequently, the Mo records reveal a later increase of hypoxia that followed the population growth in the watershed (“suburbanization”). As that population stabilized and sewers gradually replaced septic systems, wastewater N was diverted from groundwater to the Warwick WWTF on the Pawtuxet River. Likely as a result, hypoxia in the outer portion of Greenwich Bay decreased, while the innermost portion of Greenwich Bay was subject to continued regular hypoxia.

Caution is required, however, when interpreting historic profiles of Mo that indicate increasing concentrations with depth, recognizing the potential for Mo accumulation in deeper sediments at sites where sulfate reducing activity is slight. Such profiles may be interpreted as indicative of extended periods of hypoxia when in fact, the opposite was likely, and any historical study needs to assess whether low rates of sulfate reduction and sedimentation and/or bioirrigation could be the cause. A geochemical marker that indicates historical rates of sulfate reduction could allow differentiation of elevated Mo concentrations due to hypoxia from those due to downward diffusion or bioirrigation, but we are currently unaware of such a marker.

The question of the source of elevated Mo in deeper sediments notwithstanding, the concurrence of Mo profiles and watershed influences on N loading to the waters of Narragansett Bay illustrates the utility of Mo to reconstruct the history of hypoxia in marine settings. Combined with historical records of nitrogen loading from WWTFs and estimates of groundwater loads based on watershed land use, this approach to reconstructing the prevalence of hypoxia presents a potential tool for deriving load-response relationships between nitrogen and hypoxia for estuarine waterbodies.

## Supplementary Material

Supplement1

## Figures and Tables

**Table 1:** Locations and characteristics of Narragansett Bay Water Quality Monitoring Stations sampled in this study.^a^

Label	Site	Latitude (N)	Longitude (W)	Depth (m)	Bottom habitat
BR	Bullock’s Reach	41°43.980’	71°22.130’	7–9	Organic mud, sand, and silt
		Polluted with nutrients and pathogens. Downstream of Fields Point Wastewater Treatment Facility. Influenced by CSO and WWTF discharges. Vulnerable to hypoxia & occasional anoxia.			
CP	Conimicut Point	41°42.828’	71°20.628’	7–9	Sand, silt, mostly organic mud
		Polluted with nutrients and pathogens. Upper Bay area vulnerable to periodic hypoxia in bottom waters. Important area to assess changes due to WWTF upgrades.			
NP	North Prudence Island	41°40.239’	71°21.322’	10–11	Sand, silt, some organic mud
		Upper Bay area vulnerable to periodic hypoxia in bottom waters. Representative of the west side of Upper Bay.			
GB	Greenwich Bay	41°41.090’	71°26.762’	1.5–3.5	Sand, silt
		Polluted with nutrients and pathogens. Vulnerable to hypoxia and occasional anoxia throughout the water column. Location of massive fish kill in August 2003.			
SR	Sally Rock	41°40.517’	71°25.441’	1.5–3.5	Sand, silt
		Polluted with nutrients and pathogens. Vulnerable to hypoxia and occasional anoxia.			
PP	Poppasquash Point	41°38.988’	71°19.054’	7–9	Sand and silt
		Vulnerable to hypoxia. Well-flushed, occasional hypoxia. Representative of east side of Upper Bay			
QP	Quonset Point	41°35.375’	71°22.748’	7–9	Sand and silt
		Well-flushed, occasional hypoxia			

a http://www.dem.ri.gov/programs/emergencyresponse/bart/stations.php

**Table 2:** Sedimentation rates measured in 2012 cores from Narragansett Bay Water Quality Monitoring Stations.

Station	Depths analyzed	Rate (cm yr^−1^)				
		CRS (range)			CIC	r^2^
BR	4 to 27 cm	0.249	–	0.665	0.279	0.88
CP	3 to 30 cm	0.283	–	0.384	0.241	0.92
NP	0 to 17 cm	0.071	–	0.210	0.140	0.94
PP	0 to 25 cm	0.234	–	0.452	0.347	0.97
GB	0 to 21 cm	0.107	–	0.626	0.233	0.95
SR	2 to 25 cm	0.186	–	0.429	0.228	0.98
QP	0 to 30 cm	0.156	–	0.567	0.433	0.90

## Data Availability

Data will be made available on request.

## HIGHLIGHTS

- Sediment core profiles of molybdenum reflect hypoxia through 20th century.
- Trends of hypoxia correlate with changes in watershed wastewater infrastructure.
- Hypoxia follows improvement / deterioration of treatment facilities in urban area.
- Population growth, implementation of sewers more important in suburban watershed.
- Partially enclosed sub-estuaries more susceptible to hypoxia than open bay sites.
