# Supplementary material for: Modern history of hypoxia in Narragansett Bay: The geochemical record
Source: Sci Total Environ. Author manuscript; Available in PMC 2025 Nov 25. (PMC11463733; doi:10.1016/j.scitotenv.2024.176007)
Supplement: Supplement1 [file NIHMS2024585-supplement-Supplement1.docx]

**Modern history of hypoxia in Narragansett Bay: the geochemical record Supplementary data**

Warren S. Boothman^a^^[[1]](#footnote-1)^* and Laura Coiro^a^

^a^U.S. Environmental Protection Agency, Office of Research and Development, Center for Environmental Measurement and Modeling, Atlantic Coastal Environmental Sciences Division, 27 Tarzwell Drive, Narragansett, RI, 02882 USA

# ^210^Pb Gamma Analytical data and Continuous Rate of Supply (CRS) model dating calculations.

The data and calculations for dating the Narragansett Bay sediment cores using gamma radiochemical sample analyses and the Continuous Rate of Supply (CRS) model (Appleby and Oldfield 1978) to estimate the ages of sediment horizons are presented on the next 3 pages (Table S1). The table is presented in 3 parts:

- Table S1(a) provides the sample preparation and counting data from the analysis of gamma ray emissions from the samples;
- Table S1(b) gives the calculated values from the gamma analyses and the CRS model calculations;
- Table S1(c). lists the variables, definitions and formulae used to perform the CRS model calculations for each sediment core.

Table S1(a). ^210^Pb Gamma Analysis sample preparation and counting data

|  |  |  |  |  |  |  |  |  |  | 210-Pb | | 226-Ra | |
| --- | --- | --- | --- | --- | --- | --- | --- | --- | --- | --- | --- | --- | --- |
| Sample | Core ID | top (cm) | - | bottom (cm) | Tube + cap (g) | Tube + dry sediment (g) | Sample vol. (ml) | Dry sediment (g) | Count Time (s) | 46 keV | +/-error | 352 keV | +/-error |
| 1029 | BR3 | 0 | - | 1 | 5.44 | 8.62 | 4.0 | 3.18 | 172161 | 741 | 7.94% | 914 | 5.15% |
| 1030 | BR3 | 1 | - | 2 | 5.35 | 8.61 | 4.0 | 3.26 | 72769 | 374 | 10.25% | 460 | 6.65% |
| 1031 | BR3 | 2 | - | 3 | 5.43 | 9.15 | 4.0 | 3.72 | 84819 | 521 | 8.34% | 499 | 6.95% |
| 1032 | BR3 | 3 | - | 4 | 5.39 | 8.53 | 4.0 | 3.14 | 62672 | 418 | 8.83% | 410 | 6.95% |
| 1033 | BR3 | 4 | - | 5 | 5.38 | 8.66 | 4.0 | 3.28 | 80920 | 581 | 7.20% | 538 | 6.34% |
| 1035 | BR3 | 6 |  | 7 | 5.59 | 8.51 | 4.0 | 2.92 | 74798 | 281 | 12.97% | 295 | 8.80% |
| 1038 | BR3 | 9 | - | 10 | 5.58 | 8.44 | 4.0 | 2.86 | 170369 | 601 | 9.42% | 1172 | 4.53% |
| 1043 | BR3 | 14 | - | 15 | 5.37 | 8.33 | 4.0 | 2.96 | 456451 | 1084 | 7.56% | 2646 | 2.96% |
| 1048 | BR3 | 19 | - | 20 | 5.43 | 8.52 | 4.0 | 3.09 | 346501 | 804 | 9.36% | 2363 | 3.11% |
| 1050 | BR3 | 21 | - | 22 | 5.43 | 8.23 | 4.0 | 2.80 | 233453 | 364 | 18.20% | 1438 | 3.93% |
| 1053 | BR3 | 24 | - | 25 | 5.44 | 8.43 | 4.0 | 2.99 | 260567 | 625 | 10.33% | 1590 | 3.76% |
| 1055 | BR3 | 26 | - | 27 | 5.37 | 8.34 | 4.0 | 2.97 | 242952 | 376 | 17.49% | 1338 | 4.14% |
| 1058 | BR3 | 29 | - | 30 | 5.58 | 8.58 | 4.0 | 3.00 | 196966 | 506 | 11.58% | 1223 | 4.63% |
| 1059 | BR3 | 30 | - | 31 | 5.42 | 8.38 | 4.0 | 2.96 | 179649 | 384 | 14.67% | 1111 | 4.43% |
|  |  |  |  |  |  |  |  |  |  |  |  |  |  |
|  |  |  |  |  |  |  |  |  |  |  |  |  |  |
| 1314 | CP2 | 0 | - | 1 | 5.43 | 8.86 | 4.0 | 3.43 | 83681 | 593 | 7.23% | 554 | 6.11% |
| 1315 | CP2 | 1 | - | 2 | 5.34 | 8.65 | 4.0 | 3.31 | 84403 | 513 | 8.42% | 610 | 5.76% |
| 1316 | CP2 | 2 | - | 3 | 5.38 | 8.62 | 4.0 | 3.24 | 72276 | 473 | 8.47% | 543 | 5.98% |
| 1317 | CP2 | 3 | - | 4 | 5.39 | 8.68 | 4.0 | 3.29 | 99092 | 517 | 8.92% | 591 | 6.18% |
| 1318 | CP2 | 4 | - | 5 | 5.41 | 8.62 | 4.0 | 3.21 | 83722 | 569 | 7.66% | 519 | 6.08% |
| 1320 | CP2 | 6 |  | 7 | 5.40 | 9.05 | 4.0 | 3.65 | 98942 | 454 | 9.90% | 547 | 6.43% |
| 1323 | CP2 | 9 | - | 10 | 5.44 | 8.30 | 4.0 | 2.86 | 112027 | 398 | 11.35% | 633 | 6.18% |
| 1328 | CP2 | 14 | - | 15 | 5.38 | 8.38 | 4.0 | 3.00 | 259546 | 552 | 10.60% | 1526 | 3.90% |
| 1333 | CP2 | 19 | - | 20 | 5.38 | 8.23 | 4.0 | 2.85 | 341895 | 785 | 9.24% | 2061 | 3.40% |
| 1343 | CP2 | 29 | - | 30 | 5.44 | 9.08 | 4.0 | 3.64 | 431027 | 739 | 11.06% | 2671 | 2.94% |
|  |  |  |  |  |  |  |  |  |  |  |  |  |  |
|  |  |  |  |  |  |  |  |  |  |  |  |  |  |
| 1167 | NP2 | 0 | - | 1 | 5.45 | 8.55 | 4.0 | 3.10 | 77880 | 635 | 6.59% | 448 | 7.18% |
| 1168 | NP2 | 1 | - | 2 | 5.56 | 8.66 | 4.0 | 3.10 | 71498 | 466 | 8.56% | 413 | 7.51% |
| 1169 | NP2 | 2 | - | 3 | 5.44 | 8.73 | 4.0 | 3.29 | 73505 | 415 | 8.21% | 514 | 6.32% |
| 1170 | NP2 | 3 | - | 4 | 5.38 | 8.84 | 4.0 | 3.46 | 138527 | 804 | 6.89% | 885 | 5.12% |
| 1171 | NP2 | 4 | - | 5 | 5.38 | 8.64 | 4.0 | 3.26 | 101349 | 529 | 7.99% | 631 | 5.44% |
| 1173 | NP2 | 6 |  | 7 | 5.41 | 8.42 | 4.0 | 3.01 | 80724 | 370 | 10.37% | 358 | 7.91% |
| 1175 | NP2 | 8 | - | 9 | 5.37 | 8.91 | 4.0 | 3.54 | 175136 | 685 | 8.75% | 1146 | 4.75% |
| 1177 | NP2 | 10 | - | 11 | 5.48 | 8.54 | 4.0 | 3.06 | 577158 | 1058 | 8.63% | 4168 | 2.35% |
| 1183 | NP2 | 16 | - | 17 | 5.31 | 8.29 | 4.0 | 2.98 | 262434 | 395 | 13.63% | 1612 | 3.86% |
|  |  |  |  |  |  |  |  |  |  |  |  |  |  |
|  |  |  |  |  |  |  |  |  |  |  |  |  |  |
| 1069 | PP2 | 0 | - | 1 | 5.39 | 8.96 | 4.0 | 3.57 | 101045 | 612 | 7.91% | 724 | 5.48% |
| 1070 | PP2 | 1 | - | 2 | 5.45 | 8.75 | 4.0 | 3.30 | 184149 | 981 | 5.73% | 1293 | 3.84% |
| 1071 | PP2 | 2 | - | 3 | 5.44 | 8.73 | 4.0 | 3.29 | 96534 | 478 | 8.33% | 605 | 5.63% |
| 1072 | PP2 | 3 | - | 4 | 5.43 | 8.79 | 4.0 | 3.36 | 136006 | 785 | 6.92% | 1018 | 4.54% |
| 1073 | PP2 | 4 | - | 5 | 5.39 | 8.55 | 4.0 | 3.16 | 105369 | 496 | 9.41% | 744 | 5.37% |
| 1075 | PP2 | 6 |  | 7 | 5.36 | 8.57 | 4.0 | 3.21 | 178592 | 831 | 7.62% | 922 | 5.15% |
| 1078 | PP2 | 9 | - | 10 | 5.38 | 8.82 | 4.0 | 3.44 | 344767 | 1423 | 5.92% | 2670 | 2.92% |
| 1083 | PP2 | 14 | - | 15 | 5.42 | 8.83 | 4.0 | 3.41 | 259433 | 746 | 8.55% | 1872 | 3.34% |
| 1093 | PP2 | 24 | - | 25 | 5.36 | 8.68 | 4.0 | 3.32 | 241756 | 497 | 13.25% | 1881 | 3.47% |
|  |  |  |  |  |  |  |  |  |  |  |  |  |  |
|  |  |  |  |  |  |  |  |  |  |  |  |  |  |
| 851 | GB1 | 0 | - | 1 | 5.39 | 8.02 | 4.0 | 2.63 | 167980 | 790 | 7.33% | 1225 | 4.19% |
| 852 | GB1 | 1 |  | 2 | 5.58 | 8.60 | 4.0 | 3.02 | 149825 | 683 | 7.27% | 805 | 5.27% |
| 853 | GB1 | 2 |  | 3 | 5.42 | 8.84 | 4.0 | 3.42 | 155507 | 667 | 8.53% | 922 | 4.84% |
| 854 | GB1 | 3 |  | 4 | 5.38 | 8.58 | 4.0 | 3.20 | 174521 | 879 | 6.88% | 1464 | 3.59% |
| 855 | GB1 | 4 | - | 5 | 5.48 | 8.86 | 4.0 | 3.38 | 94758 | 457 | 9.84% | 1015 | 4.00% |
| 857 | GB1 | 6 |  | 7 | 5.43 | 8.76 | 4.0 | 3.33 | 150706 | 518 | 9.73% | 1128 | 4.17% |
| 859 | GB1 | 8 | - | 9 | 5.40 | 8.85 | 4.0 | 3.45 | 98850 | 253 | 14.93% | 500 | 6.56% |
| 861 | GB1 | 10 | - | 11 | 5.36 | 8.78 | 4.0 | 3.42 | 183826 | 469 | 12.87% | 1406 | 3.92% |
| 867 | GB1 | 16 | - | 17 | 5.42 | 8.44 | 4.0 | 3.02 | 324383 | 578 | 13.01% | 1807 | 3.79% |
| 871 | GB1 | 20 | - | 21 | 5.44 | 8.75 | 4.0 | 3.31 | 353075 | 650 | 10.34% | 2364 | 3.07% |
| 874 | GB1 | 23 | - | 24 | 5.62 | 8.82 | 4.0 | 3.20 | 310870 | 652 | 11.40% | 2283 | 3.09% |
|  |  |  |  |  |  |  |  |  |  |  |  |  |  |
|  |  |  |  |  |  |  |  |  |  |  |  |  |  |
| 951 | SR2 | 0 | - | 1 | 5.37 | 8.57 | 4.0 | 3.20 | 127397 | 828 | 6.55% | 845 | 4.90% |
| 952 | SR2 | 1 | - | 2 | 5.41 | 8.31 | 4.0 | 2.90 | 78990 | 550 | 7.14% | 586 | 5.63% |
| 953 | SR2 | 2 | - | 3 | 5.59 | 8.37 | 4.0 | 2.78 | 129936 | 729 | 7.38% | 922 | 4.82% |
| 954 | SR2 | 3 | - | 4 | 5.46 | 8.12 | 4.0 | 2.66 | 91774 | 590 | 7.74% | 509 | 6.81% |
| 955 | SR2 | 4 | - | 5 | 5.32 | 8.02 | 4.0 | 2.70 | 103110 | 593 | 7.30% | 626 | 5.75% |
| 957 | SR2 | 6 |  | 7 | 5.38 | 8.38 | 4.0 | 3.00 | 158728 | 849 | 6.72% | 777 | 5.31% |
| 960 | SR2 | 9 | - | 10 | 5.40 | 8.34 | 4.0 | 2.94 | 145855 | 457 | 10.90% | 753 | 5.34% |
| 965 | SR2 | 14 | - | 15 | 5.57 | 8.68 | 4.0 | 3.11 | 255799 | 604 | 10.87% | 1336 | 4.27% |
| 970 | SR2 | 19 | - | 20 | 5.37 | 8.33 | 4.0 | 2.96 | 571978 | 919 | 10.77% | 3986 | 2.57% |
| 975 | SR2 | 24 | - | 25 | 5.48 | 8.78 | 4.0 | 3.30 | 583351 | 946 | 10.73% | 4346 | 2.48% |
| 980 | SR2 | 29 | - | 30 | 5.40 | 9.20 | 4.0 | 3.80 | 519322 | 895 | 9.90% | 4387 | 2.13% |
|  |  |  |  |  |  |  |  |  |  |  |  |  |  |
|  |  |  |  |  |  |  |  |  |  |  |  |  |  |
| 1201 | QP1 | 0 | - | 1 | 5.36 | 9.01 | 4.0 | 3.65 | 171999 | 722 | 8.41% | 1472 | 3.77% |
| 1202 | QP1 | 1 | - | 2 | 5.40 | 9.21 | 4.0 | 3.81 | 74483 | 337 | 11.48% | 680 | 5.02% |
| 1203 | QP1 | 2 | - | 3 | 5.39 | 9.36 | 4.0 | 3.97 | 164006 | 697 | 8.55% | 1498 | 3.62% |
| 1204 | QP1 | 3 | - | 5 | 5.41 | 9.59 | 4.0 | 4.18 | 112014 | 444 | 9.73% | 1161 | 3.86% |
| 1207 | QP1 | 6 |  | 7 | 5.38 | 9.42 | 4.0 | 4.04 | 238184 | 667 | 8.88% | 2100 | 3.04% |
| 1211 | QP1 | 10 | - | 11 | 5.40 | 9.31 | 4.0 | 3.91 | 259103 | 545 | 12.35% | 2237 | 2.90% |
| 1217 | QP1 | 16 | - | 17 | 5.40 | 9.16 | 4.0 | 3.76 | 193059 | 612 | 9.93% | 1707 | 3.31% |
| 1229 | QP1 | 28 | - | 29 | 5.41 | 9.03 | 4.0 | 3.62 | 427100 | 1181 | 7.81% | 4643 | 2.05% |
| 1235 | QP1 | 34 | - | 35 | 5.30 | 8.93 | 4.0 | 3.63 | 497709 | 1182 | 7.89% | 4743 | 2.02% |
|  |  |  |  |  |  |  |  |  |  |  |  |  |  |
|  |  |  |  |  |  |  |  |  |  |  |  |  |  |
| 1242 | QP3 | 0 | - | 1 | 5.38 | 9.25 | 4.0 | 3.87 | 106605 | 666 | 7.32% | 996 | 4.53% |
| 1243 | QP3 | 1 | - | 2 | 5.38 | 9.36 | 4.0 | 3.98 | 152347 | 852 | 6.85% | 1451 | 3.69% |
| 1244 | QP3 | 2 | - | 3 | 5.65 | 9.53 | 4.0 | 3.88 | 77610 | 428 | 9.37% | 748 | 5.01% |
| 1245 | QP3 | 3 | - | 4 | 5.42 | 9.23 | 4.0 | 3.81 | 153939 | 868 | 6.81% | 1340 | 3.99% |
| 1246 | QP3 | 4 | - | 5 | 5.35 | 9.36 | 4.0 | 4.01 | 85529 | 332 | 10.42% | 825 | 4.60% |
| 1248 | QP3 | 6 | - | 7 | 5.44 | 9.12 | 4.0 | 3.68 | 171631 | 519 | 11.11% | 1541 | 3.55% |
| 1251 | QP3 | 9 | - | 10 | 5.59 | 9.20 | 4.0 | 3.61 | 170336 | 550 | 9.65% | 1743 | 3.23% |
| 1261 | QP3 | 19 | - | 20 | 5.42 | 8.89 | 4.0 | 3.47 | 179212 | 476 | 12.34% | 1952 | 3.14% |
| 1266 | QP3 | 24 | - | 25 | 5.39 | 9.00 | 4.0 | 3.61 | 198232 | 399 | 10.97% | 2116 | 3.00% |
| 1271 | QP3 | 29 | - | 30 | 5.43 | 9.22 | 4.0 | 3.79 | 231860 | 469 | 12.87% | 1406 | 3.92% |

Table S1(b). Pb-210 Gamma Analysis and CRS model calculated values

|  |  |  |  |  | branching ratio: | | | 0.045 | branching ratio: | | | 0.358 | Activity(Pb-210)= | | | 0.0311 | /yr |
| --- | --- | --- | --- | --- | --- | --- | --- | --- | --- | --- | --- | --- | --- | --- | --- | --- | --- |
|  |  |  |  |  | 210-Pb | | | | 226-Ra | | | |  |  |  |  |  |
| Sample | Core ID | top (cm) | - | bottom (cm) | CPM | eff | Dpm/g | ± | CPM | eff | Dpm/g | ± | xs ^210^Pb | ± | Density (g cm^-3^) | Q (dpm cm^-2^) | CSR Age (y) |
| 1029 | BR3 | 0 | - | 1 | 0.258 | 0.350 | 5.16 | 0.41 | 0.319 | 0.197 | 1.42 | 0.07 | 3.49 | 0.45 | 0.4277 | 1.49 | 1.5 |
| 1030 | BR3 | 1 | - | 2 | 0.308 | 0.350 | 6.01 | 0.62 | 0.379 | 0.197 | 1.65 | 0.11 | 4.34 | 0.64 | 0.4453 | 1.71 | 3.2 |
| 1031 | BR3 | 2 | - | 3 | 0.369 | 0.350 | 6.29 | 0.52 | 0.353 | 0.197 | 1.35 | 0.09 | 4.63 | 0.55 | 0.4684 | 2.05 | 5.4 |
| 1032 | BR3 | 3 | - | 4 | 0.400 | 0.350 | 8.09 | 0.71 | 0.393 | 0.197 | 1.77 | 0.12 | 6.43 | 0.74 | 0.4651 | 2.58 | 8.5 |
| 1033 | BR3 | 4 | - | 5 | 0.431 | 0.350 | 8.34 | 0.60 | 0.399 | 0.197 | 1.72 | 0.11 | 6.67 | 0.63 | 0.4653 | 3.05 | 12.5 |
| 1035 | BR3 | 6 |  | 7 | 0.225 | 0.350 | 4.90 | 0.64 | 0.237 | 0.197 | 1.40 | 0.12 | 3.24 | 0.66 | 0.4453 | 4.51 | 19.6 |
| 1038 | BR3 | 9 | - | 10 | 0.212 | 0.350 | 4.70 | 0.44 | 0.413 | 0.197 | 1.73 | 0.08 | 3.03 | 0.48 | 0.4853 | 4.38 | 28.4 |
| 1043 | BR3 | 14 | - | 15 | 0.142 | 0.350 | 3.06 | 0.23 | 0.348 | 0.197 | 1.67 | 0.05 | 1.39 | 0.29 | 0.5239 | 5.58 | 44.8 |
| 1048 | BR3 | 19 | - | 20 | 0.139 | 0.350 | 2.86 | 0.27 | 0.409 | 0.197 | 1.88 | 0.06 | 1.20 | 0.32 | 0.5479 | 3.47 | 62.0 |
| 1050 | BR3 | 21 | - | 22 | 0.094 | 0.350 | 2.12 | 0.39 | 0.370 | 0.197 | 1.87 | 0.29 | 0.46 | 0.42 | 0.5671 | 0.92 | 68.7 |
| 1053 | BR3 | 24 | - | 25 | 0.133 | 0.350 | 2.83 | 0.29 | 0.366 | 0.197 | 1.74 | 0.07 | 1.16 | 0.34 | 0.4583 | 1.24 | 80.8 |
| 1055 | BR3 | 26 | - | 27 | 0.093 | 0.350 | 1.99 | 0.35 | 0.330 | 0.197 | 1.58 | 0.26 | 0.32 | 0.39 | 0.6163 | 0.80 | 91.8 |
| 1058 | BR3 | 29 | - | 30 | 0.127 | 0.350 | 2.68 | 0.31 | 0.373 | 0.197 | 1.76 | 0.08 | 1.02 | 0.36 | 0.6370 | 1.26 | 125.2 |
| 1059 | BR3 | 30 | - | 31 | 0.128 | 0.350 | 2.75 | 0.40 | 0.371 | 0.197 | 1.78 | 0.32 | 1.09 | 0.44 | 0.6723 | 0.69 |  |
|  |  |  |  |  |  |  |  |  | Mean, s.d. = | | 1.67 | 0.17 |  | sum (Q_0_)= | | 33.71 |  |
|  |  |  |  |  |  |  |  |  |  |  |  |  |  |  |  |  |  |
| 1314 | CP2 | 0 | - | 1 | 0.425 | 0.350 | 7.87 | 0.57 | 0.397 | 0.197 | 1.64 | 0.10 | 6.22 | 0.60 | 0.4717 | 2.93 | 2.6 |
| 1315 | CP2 | 1 | - | 2 | 0.365 | 0.350 | 7.00 | 0.59 | 0.434 | 0.197 | 1.86 | 0.11 | 5.34 | 0.62 | 0.4835 | 2.76 | 5.3 |
| 1316 | CP2 | 2 | - | 3 | 0.393 | 0.350 | 7.69 | 0.65 | 0.451 | 0.197 | 1.97 | 0.12 | 6.04 | 0.68 | 0.4696 | 2.71 | 8.1 |
| 1317 | CP2 | 3 | - | 4 | 0.313 | 0.350 | 6.04 | 0.54 | 0.358 | 0.197 | 1.54 | 0.10 | 4.39 | 0.57 | 0.4729 | 2.46 | 10.9 |
| 1318 | CP2 | 4 | - | 5 | 0.408 | 0.350 | 8.07 | 0.62 | 0.372 | 0.197 | 1.64 | 0.10 | 6.41 | 0.65 | 0.4901 | 2.60 | 14.2 |
| 1320 | CP2 | 6 |  | 7 | 0.275 | 0.350 | 4.79 | 0.47 | 0.332 | 0.197 | 1.29 | 0.08 | 3.13 | 0.51 | 0.5132 | 4.79 | 21.3 |
| 1323 | CP2 | 9 | - | 10 | 0.213 | 0.350 | 4.73 | 0.54 | 0.339 | 0.197 | 1.68 | 0.10 | 3.08 | 0.57 | 0.5055 | 4.75 | 30.2 |
| 1328 | CP2 | 14 | - | 15 | 0.128 | 0.350 | 2.70 | 0.29 | 0.353 | 0.197 | 1.67 | 0.07 | 1.05 | 0.35 | 0.5657 | 5.52 | 45.4 |
| 1333 | CP2 | 19 | - | 20 | 0.138 | 0.350 | 3.07 | 0.28 | 0.362 | 0.197 | 1.80 | 0.06 | 1.41 | 0.35 | 0.6128 | 3.63 | 61.5 |
| 1343 | CP2 | 29 | - | 30 | 0.103 | 0.350 | 1.79 | 0.20 | 0.372 | 0.197 | 1.45 | 0.04 | 0.14 | 0.28 | 0.8242 | 5.59 |  |
|  |  |  |  |  |  |  |  |  | Mean, s.d. = | | 1.65 | 0.20 |  | sum (Q_0_)= | | 37.73 |  |
|  |  |  |  |  |  |  |  |  |  |  |  |  |  |  |  |  |  |
| 1167 | NP2 | 0 | - | 1 | 0.489 | 0.350 | 10.02 | 0.66 | 0.345 | 0.197 | 1.58 | 0.11 | 8.34 | 0.67 | 0.4003 | 3.34 | 4.7 |
| 1168 | NP2 | 1 | - | 2 | 0.391 | 0.350 | 8.01 | 0.69 | 0.347 | 0.197 | 1.59 | 0.12 | 6.33 | 0.69 | 0.4182 | 3.00 | 9.7 |
| 1169 | NP2 | 2 | - | 3 | 0.339 | 0.350 | 6.54 | 0.54 | 0.420 | 0.197 | 1.81 | 0.11 | 4.86 | 0.55 | 0.4773 | 2.50 | 14.5 |
| 1170 | NP2 | 3 | - | 4 | 0.348 | 0.350 | 6.39 | 0.44 | 0.383 | 0.197 | 1.57 | 0.08 | 4.71 | 0.45 | 0.5180 | 2.38 | 19.8 |
| 1171 | NP2 | 4 | - | 5 | 0.313 | 0.350 | 6.10 | 0.49 | 0.374 | 0.197 | 1.62 | 0.09 | 4.42 | 0.50 | 0.4782 | 2.27 | 25.9 |
| 1173 | NP2 | 6 |  | 7 | 0.238 | 0.350 | 5.02 | 0.52 | 0.266 | 0.197 | 1.84 | 0.12 | 3.34 | 0.53 | 0.5043 | 3.81 | 39.8 |
| 1175 | NP2 | 8 | - | 9 | 0.235 | 0.350 | 4.21 | 0.37 | 0.393 | 0.197 | 1.57 | 0.07 | 2.53 | 0.39 | 0.7385 | 3.64 | 63.0 |
| 1177 | NP2 | 10 | - | 11 | 0.110 | 0.350 | 2.28 | 0.20 | 0.433 | 0.197 | 1.79 | 0.04 | 0.60 | 0.23 | 0.5488 | 2.02 | 91.4 |
| 1183 | NP2 | 16 | - | 17 | 0.090 | 0.350 | 1.92 | 0.26 | 0.369 | 0.197 | 1.75 | 0.07 | 0.24 | 0.29 | 0.5730 | 1.42 |  |
|  |  |  |  |  |  |  |  |  | Mean, s.d. = | | 1.68 | 0.11 |  | sum (Q_0_)= | | 24.40 |  |
|  |  |  |  |  |  |  |  |  |  |  |  |  |  |  |  |  |  |
| 1069 | PP2 | 0 | - | 1 | 0.363 | 0.350 | 6.46 | 0.51 | 0.430 | 0.197 | 1.71 | 0.09 | 4.68 | 0.55 | 0.5311 | 2.49 | 2.2 |
| 1070 | PP2 | 1 | - | 2 | 0.320 | 0.350 | 6.15 | 0.35 | 0.421 | 0.197 | 1.81 | 0.07 | 4.37 | 0.40 | 0.5997 | 2.56 | 4.7 |
| 1071 | PP2 | 2 | - | 3 | 0.297 | 0.350 | 5.73 | 0.48 | 0.376 | 0.197 | 1.62 | 0.09 | 3.95 | 0.51 | 0.6142 | 2.53 | 7.3 |
| 1072 | PP2 | 3 | - | 4 | 0.346 | 0.350 | 6.54 | 0.45 | 0.449 | 0.197 | 1.90 | 0.09 | 4.76 | 0.49 | 0.6008 | 2.65 | 10.3 |
| 1073 | PP2 | 4 | - | 5 | 0.282 | 0.350 | 5.67 | 0.53 | 0.424 | 0.197 | 1.90 | 0.10 | 3.90 | 0.57 | 0.5999 | 2.60 | 13.5 |
| 1075 | PP2 | 6 |  | 7 | 0.279 | 0.350 | 5.52 | 0.42 | 0.310 | 0.197 | 1.37 | 0.07 | 3.74 | 0.46 | 0.5834 | 4.52 | 20.0 |
| 1078 | PP2 | 9 | - | 10 | 0.248 | 0.350 | 4.57 | 0.27 | 0.465 | 0.197 | 1.92 | 0.06 | 2.79 | 0.33 | 0.6051 | 5.82 | 31.0 |
| 1083 | PP2 | 14 | - | 15 | 0.173 | 0.350 | 3.21 | 0.27 | 0.433 | 0.197 | 1.80 | 0.06 | 1.43 | 0.33 | 0.7075 | 6.93 | 52.4 |
| 1093 | PP2 | 24 | - | 25 | 0.123 | 0.350 | 2.36 | 0.31 | 0.467 | 0.197 | 1.99 | 0.07 | 0.58 | 0.37 | 0.7544 | 7.36 |  |
|  |  |  |  |  |  |  |  |  | Mean, s.d. = | | 1.78 | 0.19 |  | sum (Q_0_)= | | 37.46 |  |
|  |  |  |  |  |  |  |  |  |  |  |  |  |  |  |  |  |  |
| 851 | GB1 | 0 | - | 1 | 0.282 | 0.350 | 6.81 | 0.50 | 0.298 | 0.197 | 1.60 | 0.07 | 5.01 | 0.56 | 0.2765 | 1.39 | 1.6 |
| 852 | GB1 | 1 |  | 2 | 0.274 | 0.350 | 5.75 | 0.42 | 0.322 | 0.197 | 1.51 | 0.08 | 3.95 | 0.48 | 0.4822 | 1.70 | 3.6 |
| 853 | GB1 | 2 |  | 3 | 0.257 | 0.350 | 4.78 | 0.41 | 0.356 | 0.197 | 1.47 | 0.07 | 2.98 | 0.48 | 0.9169 | 2.43 | 6.8 |
| 854 | GB1 | 3 |  | 4 | 0.302 | 0.350 | 6.00 | 0.41 | 0.503 | 0.197 | 2.02 | 0.08 | 4.20 | 0.48 | 0.9591 | 3.37 | 11.8 |
| 855 | GB1 | 4 | - | 5 | 0.289 | 0.350 | 5.44 | 0.53 | 0.503 | 0.197 | 2.11 | 0.08 | 3.64 | 0.59 | 1.5810 | 4.98 | 20.9 |
| 857 | GB1 | 6 |  | 7 | 0.206 | 0.350 | 3.93 | 0.38 | 0.449 | 0.197 | 1.69 | 0.08 | 2.13 | 0.45 | 0.6836 | 6.54 | 39.1 |
| 859 | GB1 | 8 | - | 9 | 0.154 | 0.350 | 2.83 | 0.42 | 0.303 | 0.197 | 2.06 | 0.11 | 1.03 | 0.49 | 0.6923 | 2.18 | 48.5 |
| 861 | GB1 | 10 | - | 11 | 0.153 | 0.350 | 2.84 | 0.37 | 0.459 | 0.197 | 2.06 | 0.08 | 1.04 | 0.44 | 0.7640 | 1.51 | 57.1 |
| 867 | GB1 | 16 | - | 17 | 0.107 | 0.350 | 2.25 | 0.29 | 0.334 | 0.197 | 1.57 | 0.06 | 0.45 | 0.38 | 0.6247 | 3.11 | 89.5 |
| 871 | GB1 | 20 | - | 21 | 0.110 | 0.350 | 2.12 | 0.22 | 0.402 | 0.197 | 1.72 | 0.05 | 0.32 | 0.33 | 0.7617 | 1.07 | 118.7 |
| 874 | GB1 | 23 | - | 24 | 0.108 | 0.350 | 2.14 | 0.24 | 0.441 | 0.197 | 1.95 | 0.06 | 0.34 | 0.34 | 0.6983 | 0.72 |  |
|  |  |  |  |  |  |  |  |  | Mean, s.d. = | | 1.80 | 0.24 |  | sum (Q_0_)= | | 28.99 |  |
|  |  |  |  |  |  |  |  |  |  |  |  |  |  |  |  |  |  |
| 951 | SR2 | 0 | - | 1 | 0.390 | 0.350 | 7.74 | 0.51 | 0.258 | 0.197 | 1.14 | 0.06 | 6.29 | 0.57 | 0.5058 | 3.18 | 2.3 |
| 952 | SR2 | 1 | - | 2 | 0.418 | 0.350 | 9.15 | 0.65 | 0.305 | 0.197 | 1.49 | 0.08 | 7.70 | 0.70 | 0.5757 | 3.78 | 5.3 |
| 953 | SR2 | 2 | - | 3 | 0.337 | 0.350 | 7.69 | 0.57 | 0.286 | 0.197 | 1.46 | 0.07 | 6.24 | 0.62 | 0.5460 | 3.91 | 8.8 |
| 954 | SR2 | 3 | - | 4 | 0.386 | 0.350 | 9.21 | 0.71 | 0.193 | 0.197 | 1.03 | 0.07 | 7.76 | 0.76 | 0.5134 | 3.71 | 12.4 |
| 955 | SR2 | 4 | - | 5 | 0.345 | 0.350 | 8.11 | 0.59 | 0.224 | 0.197 | 1.18 | 0.07 | 6.67 | 0.65 | 0.5797 | 3.94 | 16.8 |
| 957 | SR2 | 6 |  | 7 | 0.321 | 0.350 | 6.79 | 0.46 | 0.294 | 0.197 | 1.39 | 0.07 | 5.35 | 0.52 | 0.5919 | 7.04 | 26.5 |
| 960 | SR2 | 9 | - | 10 | 0.188 | 0.350 | 4.06 | 0.44 | 0.310 | 0.197 | 1.49 | 0.08 | 2.62 | 0.51 | 0.5700 | 6.94 | 40.2 |
| 965 | SR2 | 14 | - | 15 | 0.142 | 0.350 | 2.89 | 0.31 | 0.313 | 0.197 | 1.43 | 0.06 | 1.45 | 0.41 | 0.6179 | 6.03 | 60.1 |
| 970 | SR2 | 19 | - | 20 | 0.096 | 0.350 | 2.07 | 0.22 | 0.418 | 0.197 | 1.85 | 0.04 | 0.62 | 0.34 | 0.7217 | 3.47 | 81.9 |
| 975 | SR2 | 24 | - | 25 | 0.097 | 0.350 | 1.87 | 0.20 | 0.447 | 0.197 | 1.67 | 0.04 | 0.43 | 0.33 | 0.8200 | 2.03 | 108.8 |
| 980 | SR2 | 29 | - | 30 | 0.103 | 0.350 | 1.73 | 0.17 | 0.507 | 0.197 | 1.76 | 0.04 | 0.28 | 0.31 | 0.9202 | 1.55 |  |
|  |  |  |  |  |  |  |  |  | Mean, s.d. = | | 1.44 | 0.26 |  | sum (Q_0_)= | | 45.59 |  |
|  |  |  |  |  |  |  |  |  |  |  |  |  |  |  |  |  |  |
| 1201 | QP1 | 0 | - | 1 | 0.252 | 0.350 | 4.38 | 0.37 | 0.373 | 0.197 | 1.45 | 0.05 | 2.64 | 0.44 | 0.5994 | 1.58 | 1.8 |
| 1202 | QP1 | 1 | - | 2 | 0.271 | 0.350 | 4.52 | 0.52 | 0.408 | 0.197 | 1.52 | 0.08 | 2.78 | 0.57 | 0.6615 | 1.71 | 3.8 |
| 1203 | QP1 | 2 | - | 3 | 0.255 | 0.350 | 4.08 | 0.35 | 0.408 | 0.197 | 1.46 | 0.05 | 2.34 | 0.42 | 0.7450 | 1.80 | 6.2 |
| 1204 | QP1 | 3 | - | 5 | 0.238 | 0.350 | 3.61 | 0.35 | 0.482 | 0.197 | 1.63 | 0.06 | 1.87 | 0.42 | 0.7838 | 3.22 | 10.8 |
| 1207 | QP1 | 6 |  | 7 | 0.168 | 0.350 | 2.64 | 0.23 | 0.529 | 0.197 | 1.86 | 0.06 | 0.90 | 0.24 | 0.7995 | 2.20 | 14.3 |
| 1211 | QP1 | 10 | - | 11 | 0.126 | 0.350 | 2.05 | 0.25 | 0.518 | 0.197 | 1.88 | 0.05 | 0.31 | 0.26 | 0.7768 | 1.91 | 17.8 |
| 1217 | QP1 | 16 | - | 17 | 0.159 | 0.350 | 2.69 | 0.27 | 0.531 | 0.197 | 1.78 | 0.06 | 0.95 | 0.36 | 0.8197 | 3.01 | 24.1 |
| 1229 | QP1 | 28 | - | 29 | 0.154 | 0.350 | 2.70 | 0.21 | 0.652 | 0.197 | 2.01 | 0.04 | 0.96 | 0.32 | 0.9171 | 9.95 | 65.2 |
| 1235 | QP1 | 34 | - | 35 | 0.125 | 0.350 | 2.18 | 0.17 | 0.572 | 0.197 | 2.08 | 0.04 | 0.44 | 0.29 | 0.9084 | 3.85 |  |
|  |  |  |  |  |  |  |  |  | Mean, s.d. = | | 1.74 | 0.24 |  | sum (Q_0_)= | | 29.22 |  |
|  |  |  |  |  |  |  |  |  |  |  |  |  |  |  |  |  |  |
| 1242 | QP3 | 0 | - | 1 | 0.375 | 0.350 | 6.15 | 0.45 | 0.421 | 0.197 | 1.54 | 0.07 | 4.62 | 0.45 | 0.6381 | 2.95 | 2.5 |
| 1243 | QP3 | 1 | - | 2 | 0.336 | 0.350 | 5.35 | 0.37 | 0.431 | 0.197 | 1.54 | 0.06 | 3.82 | 0.37 | 0.6410 | 2.70 | 4.9 |
| 1244 | QP3 | 2 | - | 3 | 0.331 | 0.350 | 5.41 | 0.51 | 0.438 | 0.197 | 1.60 | 0.08 | 3.88 | 0.51 | 0.6965 | 2.58 | 7.4 |
| 1245 | QP3 | 3 | - | 4 | 0.338 | 0.350 | 5.64 | 0.38 | 0.382 | 0.197 | 1.42 | 0.06 | 4.11 | 0.39 | 0.7213 | 2.83 | 10.4 |
| 1246 | QP3 | 4 | - | 5 | 0.233 | 0.350 | 3.69 | 0.38 | 0.439 | 0.197 | 1.55 | 0.07 | 2.16 | 0.39 | 0.7459 | 2.30 | 13.1 |
| 1248 | QP3 | 6 | - | 7 | 0.181 | 0.350 | 3.13 | 0.35 | 0.539 | 0.197 | 8.30 | 0.29 | 1.60 | 0.35 | 0.7228 | 2.76 | 16.6 |
| 1251 | QP3 | 9 | - | 10 | 0.194 | 0.350 | 3.41 | 0.33 | 0.614 | 0.197 | 9.65 | 0.31 | 1.88 | 0.34 | 0.7647 | 3.88 | 22.3 |
| 1261 | QP3 | 19 | - | 20 | 0.159 | 0.350 | 2.92 | 0.36 | 0.654 | 0.197 | 10.68 | 0.34 | 1.39 | 0.37 | 0.8341 | 13.04 | 56.5 |
| 1266 | QP3 | 24 | - | 25 | 0.121 | 0.350 | 2.12 | 0.23 | 0.640 | 0.197 | 10.06 | 0.30 | 0.59 | 0.24 | 0.9289 | 4.36 | 88.6 |
| 1271 | QP3 | 29 | - | 30 | 0.121 | 0.350 | 2.03 | 0.26 | 0.364 | 0.197 | 5.44 | 0.21 | 0.50 | 0.27 | 0.9296 | 2.55 |  |
|  |  |  |  |  |  |  |  |  | Mean, s.d. = | | 1.53 | 0.07 |  | sum (Q_0_)= | | 39.93 |  |

Table S1(c). CRS calculations variable definitions and formulae

| Variable | Definition | Formula | Unit |
| --- | --- | --- | --- |
| top | Depth in the core at the top of the horizon |  | cm |
| bottom | Depth in the core at the bottom of the horizon |  | cm |
| mid point | Depth in the core at the center of the horizon | = [top] - [bottom] | cm |
| Tube + cap | Mass of the empty sample tube and cap |  | g |
| Tube + dry sediment | Mass of the sample tube and cap containing dry sediment |  | g |
| Sample vol. | Volume of dry sediment within the tube |  | ml |
| Dry sediment | mass of dry sediment sampled | = [Tube + dry sediment] - [Tube + cap] | g |
| Start Ct Date | Date/time radiometric counting commenced |  |  |
| End Ct Date | Date/time radiometric counting completed |  |  |
| Ct Time | Total amount of time counting radiometric emissions |  | s |
| 46 keV | Counts at energy 46 keV (Pb-210) |  | Counts |
| +/- error 210-Pb | Uncertainty in counts at 46 keV |  | " |
| 352 keV | Counts at energy 352 keV (Ra-226, used to assess supported lead activity) |  | " |
| '+/- error 226-Ra | Uncertainty in counts at 352 keV |  | " |
| branching ratio | fraction of gamma energy released at a particular energy when the Pb-210 atom decays | = 0.045 for 46 keV,  0.385 for 352 keV |  |
| CPM 210-Pb | Count rate for 46 keV (210-Pb) peak | = ([46 keV] / [Ct Time]) x 60 | Counts/min |
| eff 210-Pb | counting efficiency for sample volume at 46 keV | Table lookup function of measured energy and vial volume |  |
| Dpm/g 210-Pb | specific disintegration rate for 46 keV (210-Pb) | = ([CPM 210-Pb] / [Dry sediment]) / ([eff 210-Pb] x [branching ratio]) | Disintegrations/min/g |
| ± 210-Pb | Uncertainty in specific disintegration rate for 46 keV (210-Pb) | = [Dpm/g 210-Pb] x [+/- error 210-Pb] | " |
| CPM 226-Ra | Count rate for 352 keV (226-Ra) peak | = ([352 keV] / [Ct Time]) x 60 | Counts/min |
| eff 226-Ra | counting efficiency for sample volume at 352 keV | Table lookup function of measured energy and vial volume |  |
| Dpm/g 226-Ra | specific disintegration rate for 352 keV (226-Ra) | = ([CPM 226-Ra] / [Dry sediment]) / ([eff 226-Ra] x [branching ratio]) | Disintegrations/min/g |
| ± 226-Ra | Uncertainty in specific disintegration rate for 352 keV (226-Ra) | = [Dpm/g 226-Ra] x [+/- error 226-Ra] | " |
| xs 210Pb | Excess (unsupported) 210-Pb activity | = [Dpm/g 210-Pb] - [mean(Dpm/g 226-Ra)] | " |
| ± xs 210Pb |  | = SQRT( [± 210-Pb]^2^ + [± 226-Ra]^2^ ) | " |
| Dry wt density |  |  | g / cm^3^ |
| A_Pb-210_ | Activity constant for Pb-210 | 0.0311 | /yr |
| Q | unsupported 210-Pb activity inventory (per horizon) | =([xs 210Pb]_i_+[xs 210Pb]_i-1_)/2 x ([Dry wt density]_i_+[Dry wt density]_i-1_)/2 x ([bottom]_i_-[bottom]_i-1_) for horizon i | dpm / cm^2^ |
| Q_0_ | total unsupported 210-Pb activity inventory | =Sum([unsupported 210-Pb activity inventory]) over all horizons | dpm / cm^2^ |
| Age | age at bottom of horizon i | =LN( [Q_0_] / Σ(Qi)) / Activity(Pb-210) , for all horizons at/below horizon i | y |

# References

Appleby, P. G. and F. Oldfield. 1978. The calculation of lead-210 dates assuming a constant rate of supply of unsupported ^210^Pb to the sediment. *Catena* 5**:** 1-8.

Table S2. Quality control data for analyses of metals

*Instrumental QC*

|  | Replicate analyses RPD (%) | | Spiked blank recovery (%) | | Spiked sample recovery (%) | |
| --- | --- | --- | --- | --- | --- | --- |
| Analyte | Min. | Max. | Min. | Max. | Min. | Max. |
| Al | 6 | 14 | 96 | 102 | 97 | 110 |
| Cr | 5 | 12 | 94 | 120 | 96 | 107 |
| Cu | 13 | 13 | 92 | 112 | 96 | 103 |
| Fe | 5 | 21 | 84 | 112 | 87 | 113 |
| Mn | 5 | 21 | 97 | 113 | 94 | 103 |
| Mo | 2 | 34 | 95 | 105 | 100 | 104 |
| Ni | 8 | 21 | 95 | 115 | 91 | 106 |
| Zn | 3 | 13 | 101 | 118 | 98 | 108 |

*Analytical QC*

|  | Triplicate samples | | | | Standard reference material (MESS-3) | | | | |  |
| --- | --- | --- | --- | --- | --- | --- | --- | --- | --- | --- |
|  | RSD (%) | | | | Certified Concentration (µg/g) | Recovery (%) | | | |  |
| Analyte | Mean | Min. |  | Max. |  | Mean | Min |  | Max | |
| Al | 4.8 | 0.6 | - | 13.1 | 85900 | 91 | 70 | - | 121 | |
| Cr | 2.8 | 0.2 | - | 8.5 | 105.0 | 84 | 63 | - | 102 | |
| Cu | 3.6 | 1.1 | - | 15.1 | 33.9 | 63 | 26 | - | 101 | |
| Fe | 2.9 | 0.7 | - | 7.6 | 43400 | 86 | 75 | - | 98 | |
| Mn | 4.5 | 0.8 | - | 9.9 | 324 | 97 | 84 | - | 107 | |
| Mo | 3.1 | 0.3 | - | 7.3 | 2.78 | 95 | 84 | - | 113 | |
| Ni | 3.7 | 1.0 | - | 11.0 | 46.9 | 80 | 68 | - | 93 | |
| Zn | 2.5 | 0.2 | - | 8.9 | 159 | 76 | 60 | - | 95 | |

Table S3. Quality control data for analyses of carbon and nitrogen concentrations and isotopic ratios

|  |  | | | Replicates - rsd (%) | | |
| --- | --- | --- | --- | --- | --- | --- |
| Analyte | Values | | | Mean | Min | Max |
| % C | 1.41 | - | 4.28 % | 2.9% | 0.9% | 9.7% |
| δ^13^C | -17.00 | - | -21.34 ‰ | 1.5% | 0.2% | 7.6% |
| % N | 0.13 | - | 0.38 % | 2.6% | 0.7% | 6.4% |
| δ^15^N | 6.72 | - | 9.24 ‰ | 1.4% | 0.1% | 3.7% |

|  | Reference sediment (BCSS-1) | | | | |
| --- | --- | --- | --- | --- | --- |
| Analyte | Certified value |  | Mean | Min | Max |
| % C | 2.13 ± 0.06 % | recovery | 101% | 83% | 119% |
| δ^13^C | -23.25 ± 0.31 ‰ | error | 0.08 | 0.01 | 0.19 |
| % N | 0.19 ± 0.01 % | recovery | 103% | 86% | 125% |
| δ^15^N | 4.66 ± 0.41 ‰ | error | 0.16 | 0.00 | 0.65 |

Table S4. Concentrations of metals in Narragansett Bay sediment cores

| Site: | BR |  | Concentrations (µg/g dry sediment) | | | | | | | | | | | | | | | |
| --- | --- | --- | --- | --- | --- | --- | --- | --- | --- | --- | --- | --- | --- | --- | --- | --- | --- | --- |
| Depth (cm) | | analyte | Al | | Fe | | Mn | | Zn | | Cu | | Cr | | Ni | | Mo | |
| Top | bottom | core | 2 | 3 | 2 | 3 | 2 | 3 | 2 | 3 | 2 | 3 | 2 | 3 | 2 | 3 | 2 | 3 |
| 0 | 1 |  |  | 56410 |  | 29553 |  | 380 |  | 212 |  | 225 |  | 157 |  | 23.7 |  | 2.67 |
|  | 2 |  | 65537 |  | 33826 |  | 447 |  | 174 |  | 158 |  | 113 |  | 26.3 |  | 2.95 |  |
| 1 | 2 |  |  | 54736 |  | 29534 |  | 384 |  | 161 |  | 152 |  | 111 |  | 22.4 |  | 3.09 |
| 2 | 3 |  | 64014 | 54485 | 33618 | 29091 | 441 | 379 | 183 | 148 | 163 | 134 | 119 | 97 | 26.6 | 22.3 | 3.18 | 2.77 |
| 3 | 4 |  | 65162 | 57652 | 33437 | 29370 | 425 | 382 | 183 | 163 | 157 | 146 | 121 | 106 | 25.2 | 22.6 | 3.36 | 3.28 |
| 4 | 5 |  | 65197 | 55570 | 33703 | 30778 | 435 | 396 | 172 | 175 | 154 | 167 | 114 | 111 | 25.3 | 25.1 | 2.59 | 3.29 |
| 5 | 6 |  | 59844 | 56063 | 32420 | 30650 | 437 | 409 | 187 | 193 | 168 | 173 | 119 | 123 | 26.3 | 25.9 | 2.36 | 3.45 |
| 6 | 7 |  | 61373 | 57756 | 32711 | 30166 | 452 | 398 | 208 | 192 | 193 | 177 | 125 | 120 | 28.3 | 26.6 | 2.63 | 3.06 |
| 7 | 8 |  | 62255 | 60894 | 34011 | 29522 | 466 | 414 | 222 | 179 | 228 | 181 | 128 | 110 | 31.8 | 26.8 | 2.56 | 2.84 |
| 8 | 9 |  | 61494 | 62668 | 33927 | 30979 | 474 | 420 | 218 | 204 | 222 | 214 | 137 | 126 | 33.4 | 28.9 | 1.93 | 2.82 |
| 9 | 10 |  | 66789 | 60656 | 32033 | 29918 | 428 | 408 | 249 | 212 | 245 | 214 | 143 | 130 | 34.1 | 28.7 | 1.94 | 2.35 |
| 10 | 11 |  | 64717 | 62409 | 31345 | 31091 | 398 | 411 | 261 | 232 | 286 | 248 | 152 | 141 | 33.7 | 31.8 | 2.17 | 2.60 |
| 11 | 12 |  | 66598 | 63781 | 32025 | 31737 | 435 | 412 | 260 | 237 | 289 | 258 | 154 | 141 | 37.6 | 33.6 | 2.26 | 2.64 |
| 12 | 13 |  | 65821 | 61112 | 33137 | 28951 | 431 | 390 | 288 | 245 | 325 | 281 | 172 | 154 | 40.6 | 32.6 | 2.29 | 2.55 |
| 13 | 14 |  | 66552 | 62871 | 32923 | 29936 | 431 | 407 | 289 | 238 | 312 | 310 | 176 | 149 | 41.4 | 32.9 | 2.23 | 2.41 |
| 14 | 15 |  | 61068 | 62271 | 32236 | 30359 | 421 | 406 | 302 | 271 | 310 | 379 | 178 | 190 | 40.8 | 30.4 | 2.24 | 2.86 |
| 15 | 16 |  | 66160 | 60908 | 32198 | 28561 | 413 | 395 | 282 | 250 | 316 | 328 | 179 | 171 | 39.0 | 29.0 | 1.98 | 2.58 |
| 16 | 17 |  | 69321 | 63327 | 33778 | 31000 | 423 | 400 | 298 | 252 | 326 | 367 | 182 | 190 | 40.4 | 29.9 | 2.22 | 3.04 |
| 17 | 18 |  | 69312 | 60013 | 33132 | 30295 | 431 | 403 | 290 | 268 | 325 | 370 | 190 | 197 | 40.9 | 31.9 | 2.14 | 3.35 |
| 18 | 19 |  | 65777 | 58083 | 31197 | 32942 | 394 | 415 | 270 | 284 | 315 | 399 | 171 | 210 | 36.6 | 34.6 | 2.26 | 3.76 |
| 19 | 20 |  | 68389 | 64507 | 33007 | 33844 | 428 | 416 | 312 | 297 | 350 | 416 | 204 | 233 | 40.5 | 33.4 | 2.63 | 3.89 |
| 20 | 21 |  | 68584 | 60250 | 30887 | 34479 | 409 | 421 | 258 | 282 | 332 | 405 | 176 | 230 | 35.0 | 32.1 | 2.69 | 3.60 |
| 21 | 22 |  | 64786 | 65051 | 30379 | 35162 | 401 | 420 | 238 | 310 | 316 | 420 | 172 | 245 | 32.2 | 31.8 | 2.80 | 3.85 |
| 22 | 23 |  | 64107 | 61206 | 30836 | 35452 | 404 | 418 | 256 | 351 | 376 | 453 | 190 | 268 | 31.6 | 34.4 | 2.99 | 4.92 |
| 23 | 24 |  | 62594 | 65155 | 32328 | 32714 | 406 | 393 | 275 | 340 | 443 | 431 | 215 | 255 | 31.1 | 30.9 | 3.14 | 5.02 |
| 24 | 25 |  | 63170 | 58861 | 31850 | 33049 | 399 | 406 | 283 | 329 | 443 | 385 | 219 | 260 | 28.5 | 28.5 | 3.00 | 4.60 |
| 25 | 26 |  | 66694 | 60608 | 32703 | 33733 | 407 | 406 | 290 | 347 | 419 | 392 | 222 | 259 | 28.6 | 28.6 | 3.10 | 4.93 |
| 26 | 27 |  |  | 72875 |  | 36340 |  | 426 |  | 366 |  | 410 |  | 269 |  | 29.0 |  | 4.92 |
| 27 | 28 |  |  | 69635 |  | 33426 |  | 402 |  | 347 |  | 397 |  | 255 |  | 27.7 |  | 4.79 |
| 28 | 29 |  |  | 71946 |  | 34184 |  | 408 |  | 357 |  | 379 |  | 257 |  | 27.1 |  | 4.93 |
| 29 | 30 |  |  | 67484 |  | 32531 |  | 384 |  | 330 |  | 318 |  | 233 |  | 25.2 |  | 4.57 |
| 30 | 31 |  |  | 70094 |  | 34518 |  | 413 |  | 324 |  | 340 |  | 243 |  | 26.7 |  | 4.04 |
| 31 | 32 |  |  | 73285 |  | 34890 |  | 414 |  | 334 |  | 321 |  | 236 |  | 26.1 |  | 4.67 |

| Site: | CP | Concentrations (µg/g dry sediment) | | | | | | | | | | | | | | | | |
| --- | --- | --- | --- | --- | --- | --- | --- | --- | --- | --- | --- | --- | --- | --- | --- | --- | --- | --- |
| Depth (cm) | | analyte | Al | | Fe | | Mn | | Zn | | Cu | | Cr | | Ni | | Mo | |
| Top | bottom | core | 1 | 2 | 1 | 2 | 1 | 2 | 1 | 2 | 1 | 2 | 1 | 2 | 1 | 2 | 1 | 2 |
| **0** | 1 |  | 57195 | 50193 | 30521 | 30584 | 371 | 394 | 157 | 147 | 101 | 102 | 106 | 97 | 19.0 | 18.0 | 1.74 | 1.80 |
| **1** | 2 |  | 55922 | 47756 | 28474 | 27724 | 357 | 353 | 138 | 133 | 95 | 85 | 91 | 87 | 17.2 | 17.2 | 2.11 | 2.43 |
| **2** | 3 |  | 56577 | 52810 | 30133 | 28732 | 372 | 364 | 153 | 128 | 100 | 83 | 99 | 89 | 17.2 | 16.1 | 2.72 | 2.36 |
| **3** | 4 |  | 49235 | 56044 | 26997 | 29898 | 340 | 385 | 143 | 137 | 90 | 89 | 95 | 94 | 17.2 | 18.6 | 3.47 | 2.52 |
| **4** | 5 |  | 52334 | 46062 | 27100 | 26605 | 329 | 348 | 132 | 129 | 89 | 82 | 86 | 87 | 16.0 | 15.5 | 3.43 | 2.12 |
| **5** | 6 |  | 50378 | 52215 | 26525 | 25869 | 381 | 354 | 137 | 135 | 85 | 88 | 86 | 85 | 16.3 | 14.8 | 2.66 | 2.35 |
| **6** | 7 |  | 45320 | 54334 | 25100 | 25712 | 360 | 367 | 124 | 139 | 84 | 89 | 76 | 79 | 14.6 | 15.4 | 2.73 | 2.41 |
| **7** | 8 |  | 49002 | 54830 | 26396 | 26335 | 375 | 370 | 150 | 156 | 106 | 109 | 91 | 89 | 17.5 | 17.4 | 2.55 | 2.20 |
| **8** | 9 |  | 55362 | 52796 | 29090 | 24691 | 395 | 365 | 189 | 141 | 146 | 114 | 111 | 70 | 23.2 | 14.4 | 2.73 | 2.10 |
| **9** | 10 |  | 57544 | 50397 | 28859 | 25632 | 394 | 356 | 205 | 172 | 175 | 130 | 118 | 96 | 24.8 | 19.3 | 2.09 | 1.96 |
| **10** | 11 |  | 59268 | 51521 | 29750 | 26428 | 407 | 378 | 208 | 182 | 189 | 155 | 130 | 108 | 26.4 | 22.8 | 2.10 | 1.95 |
| **11** | 12 |  | 58206 | 57808 | 29597 | 28280 | 407 | 382 | 198 | 197 | 188 | 205 | 123 | 128 | 27.0 | 28.0 | 3.02 | 2.18 |
| **12** | 13 |  | 58440 | 48317 | 30212 | 27132 | 409 | 353 | 209 | 200 | 223 | 200 | 137 | 130 | 26.7 | 26.0 | 2.24 | 1.68 |
| **13** | 14 |  | 58055 | 47662 | 30056 | 26952 | 390 | 355 | 221 | 207 | 252 | 227 | 145 | 137 | 27.1 | 25.2 | 2.05 | 1.73 |
| **14** | 15 |  | 68347 | 55454 | 31414 | 28716 | 409 | 355 | 258 | 224 | 348 | 321 | 174 | 154 | 28.7 | 23.7 | 2.43 | 1.86 |
| **15** | 16 |  | 64464 | 50269 | 35291 | 27017 | 437 | 333 | 296 | 226 | 402 | 285 | 208 | 162 | 28.2 | 20.3 | 2.80 | 1.92 |
| **16** | 17 |  | 63989 | 52251 | 33055 | 28366 | 411 | 372 | 258 | 238 | 373 | 295 | 188 | 164 | 23.9 | 24.2 | 2.47 | 1.96 |
| **17** | 18 |  | 63803 | 42450 | 34254 | 28332 | 411 | 336 | 266 | 238 | 382 | 281 | 196 | 162 | 24.8 | 20.5 | 2.22 | 1.90 |
| **18** | 19 |  | 65413 | 57437 | 33246 | 29191 | 389 | 343 | 269 | 242 | 378 | 272 | 197 | 166 | 24.4 | 20.4 | 2.08 | 1.83 |
| **19** | 20 |  | 64927 | 56251 | 33224 | 28846 | 392 | 347 | 275 | 230 | 372 | 252 | 196 | 153 | 24.7 | 18.5 | 2.17 | 1.87 |
| **20** | 21 |  | 62449 | 52421 | 30745 | 28636 | 375 | 351 | 262 | 232 | 322 | 239 | 186 | 157 | 22.5 | 19.4 | 2.20 | 1.87 |
| **21** | 22 |  | 62862 | 50225 | 31945 | 26858 | 374 | 347 | 262 | 218 | 313 | 209 | 182 | 150 | 23.2 | 17.6 | 2.43 | 1.95 |
| **22** | 23 |  | 59003 | 56234 | 29255 | 27890 | 347 | 348 | 240 | 218 | 276 | 201 | 168 | 157 | 22.3 | 17.8 | 2.39 | 2.27 |
| **23** | 24 |  | 55850 | 58532 | 29805 | 28967 | 355 | 373 | 248 | 215 | 261 | 183 | 169 | 168 | 21.6 | 17.3 | 2.55 | 2.43 |
| **24** | 25 |  | 59128 | 55307 | 30116 | 29451 | 362 | 355 | 225 | 215 | 224 | 184 | 161 | 182 | 20.0 | 17.6 | 2.52 | 2.53 |
| **25** | 26 |  | 53856 | 63580 | 27526 | 32299 | 354 | 396 | 205 | 216 | 160 | 195 | 160 | 181 | 15.9 | 19.0 | 2.23 | 2.17 |
| **26** | 27 |  | 57906 | 55304 | 33180 | 30062 | 384 | 379 | 225 | 191 | 205 | 154 | 194 | 147 | 19.8 | 18.2 | 2.54 | 2.06 |
| **27** | 28 |  | 58421 | 55865 | 33987 | 31029 | 389 | 393 | 255 | 167 | 245 | 113 | 197 | 132 | 21.0 | 18.6 | 1.97 | 2.32 |
| **28** | 29 |  | 53926 | 62603 | 32631 | 32057 | 388 | 401 | 233 | 177 | 225 | 119 | 197 | 138 | 20.2 | 19.6 | 2.03 | 2.11 |
| **29** | 30 |  | 59868 | 50295 | 34405 | 24006 | 421 | 316 | 220 | 158 | 225 | 88 | 182 | 121 | 20.4 | 11.9 | 2.09 | 1.69 |
| **30** | 31 |  | 52860 | 44022 | 33329 | 13556 | 412 | 248 | 193 | 76 | 151 | 36 | 153 | 61 | 19.1 | 4.1 | 2.26 | 0.85 |
| **31** | 32 |  |  | 63653 |  | 28634 |  | 371 |  | 224 |  | 148 |  | 181 |  | 18.8 |  | 1.76 |

| Site: | NP | Concentrations (µg/g dry sediment) | | | | | | | | | | | | | | | | |
| --- | --- | --- | --- | --- | --- | --- | --- | --- | --- | --- | --- | --- | --- | --- | --- | --- | --- | --- |
| Depth (cm) | | analyte | Al | | Fe | | Mn | | Zn | | Cu | | Cr | | Ni | | Mo | |
| top | bottom | core | 1 | 2 | 1 | 2 | 1 | 2 | 1 | 2 | 1 | 2 | 1 | 2 | 1 | 2 | 1 | 2 |
| 0 | 1 |  | 49912 | 52761 | 32678 | 37631 | 451 | 447 | 166 | 198 | 62 | 79 | 104 | 120 | 22.6 | 26.5 | 1.22 | 1.47 |
| 1 | 2 |  | 49792 | 49474 | 32496 | 35739 | 399 | 424 | 169 | 182 | 62 | 73 | 100 | 110 | 21.5 | 24.4 | 1.42 | 1.65 |
| 2 | 3 |  | 48553 | 51311 | 31005 | 33474 | 386 | 413 | 161 | 175 | 64 | 66 | 85 | 104 | 20.9 | 22.7 | 1.45 | 2.19 |
| 3 | 4 |  | 51864 | 49915 | 32099 | 35621 | 420 | 440 | 173 | 190 | 67 | 78 | 99 | 111 | 21.9 | 24.9 | 1.85 | 3.83 |
| 4 | 5 |  | 49434 | 55538 | 33836 | 37097 | 436 | 469 | 185 | 199 | 70 | 95 | 118 | 118 | 19.6 | 27.0 | 1.66 | 3.83 |
| 5 | 6 |  | 45589 | 48113 | 33328 | 38741 | 435 | 493 | 191 | 213 | 80 | 112 | 111 | 133 | 24.3 | 30.8 | 1.87 | 3.43 |
| 6 | 7 |  | 46177 | 49946 | 32631 | 37387 | 435 | 464 | 207 | 208 | 89 | 105 | 114 | 129 | 24.4 | 29.1 | 2.56 | 3.61 |
| 7 | 8 |  | 46526 | 47982 | 32908 | 30275 | 456 | 435 | 234 | 196 | 103 | 84 | 127 | 101 | 28.8 | 24.1 | 2.88 | 2.90 |
| 8 | 9 |  | 46551 | 45105 | 32079 | 27892 | 435 | 454 | 242 | 217 | 123 | 96 | 138 | 101 | 31.3 | 24.7 | 1.99 | 2.67 |
| 9 | 10 |  | 43668 | 48764 | 30814 | 30524 | 427 | 471 | 243 | 232 | 103 | 114 | 145 | 121 | 28.4 | 27.4 | 2.05 | 2.28 |
| 10 | 11 |  | 57048 | 52328 | 37033 | 32657 | 475 | 456 | 265 | 237 | 114 | 122 | 197 | 143 | 24.6 | 26.6 | 2.34 | 2.00 |
| 11 | 12 |  | 61884 | 52931 | 40984 | 33528 | 524 | 422 | 293 | 242 | 122 | 116 | 205 | 152 | 29.2 | 24.7 | 2.55 | 2.28 |
| 12 | 13 |  |  | 46669 |  | 34472 |  | 438 |  | 233 |  | 100 |  | 159 |  | 25.1 |  | 2.54 |
| 13 | 14 |  |  | 44099 |  | 33878 |  | 431 |  | 215 |  | 80 |  | 159 |  | 22.5 |  | 1.88 |
| 14 | 15 |  |  | 40005 |  | 34979 |  | 423 |  | 238 |  | 80 |  | 183 |  | 23.6 |  | 1.94 |
| 15 | 16 |  |  | 42965 |  | 33739 |  | 406 |  | 224 |  | 70 |  | 167 |  | 22.8 |  | 1.95 |
| 16 | 17 |  |  | 45786 |  | 36548 |  | 453 |  | 248 |  | 81 |  | 193 |  | 23.6 |  | 1.84 |
| 17 | 18 |  |  | 46649 |  | 11011 |  | 386 |  | 283 |  | 107 |  | 141 |  | 23.9 |  | 0.21 |
| 18 | 19 |  |  | 47918 |  | 36744 |  | 438 |  | 252 |  | 107 |  | 183 |  | 23.5 |  | 2.07 |
| 20 | 21 |  |  | 46674 |  | 32445 |  | 423 |  | 196 |  | 57 |  | 132 |  | 20.2 |  | 1.80 |
| 21 | 22 |  |  | 43118 |  | 28340 |  | 431 |  | 154 |  | 38 |  | 107 |  | 16.3 |  | 1.44 |
| 22 | 23 |  |  | 44496 |  | 27317 |  | 436 |  | 134 |  | 27 |  | 90 |  | 15.3 |  | 1.76 |
| 23 | 24 |  |  | 43878 |  | 24938 |  | 405 |  | 128 |  | 18 |  | 87 |  | 15.1 |  | 1.61 |
| 24 | 25 |  |  | 46607 |  | 25915 |  | 427 |  | 106 |  | 12 |  | 73 |  | 13.5 |  | 1.80 |
| 25 | 26 |  |  | 46529 |  | 25587 |  | 431 |  | 92 |  | 4 |  | 66 |  | 13.5 |  | 2.14 |
| 26 | 27 |  |  | 49790 |  | 27982 |  | 439 |  | 76 |  | ND |  | 61 |  | 14.8 |  | 1.88 |
| 27 | 28 |  |  | 50409 |  | 25927 |  | 418 |  | 84 |  | 3 |  | 65 |  | 14.4 |  | 1.84 |
| 28 | 29 |  |  | 50082 |  | 26881 |  | 429 |  | 63 |  | ND |  | 55 |  | 14.7 |  | 1.87 |
| 29 | 30 |  |  | 48117 |  | 24410 |  | 405 |  | 53 |  | ND |  | 48 |  | 13.1 |  | 1.76 |

| Site: | GB | Concentrations (µg/g dry sediment) | | | | | | | | | | | | | | | | |
| --- | --- | --- | --- | --- | --- | --- | --- | --- | --- | --- | --- | --- | --- | --- | --- | --- | --- | --- |
| Depth (cm) | | analyte | Al | | Fe | | Mn | | Zn | | Cu | | Cr | | Ni | | Mo | |
| top | bottom | core | 1 | 3 | 1 | 3 | 1 | 3 | 1 | 3 | 1 | 3 | 1 | 3 | 1 | 3 | 1 | 3 |
| 0 | 1 |  |  | 51607 |  | 21353 |  | 375 |  | 218 |  | 86 |  | 171 |  | 11.5 |  | 3.78 |
| 1 | 2 |  |  | 52872 |  | 22096 |  | 372 |  | 227 |  | 97 |  | 207 |  | 13.1 |  | 4.15 |
| 2 | 3 |  |  | 49991 |  | 22638 |  | 365 |  | 213 |  | 99 |  | 207 |  | 13.7 |  | 4.11 |
| 3 | 4 |  |  | 50776 |  | 22378 |  | 350 |  | 246 |  | 76 |  | 219 |  | 12.1 |  | 4.24 |
| 4 | 5 |  | 59124 | 57842 | 23567 | 24270 | 358 | 362 | 161 | 207 | 107 | 50 | 195 | 195 | 13.9 | 12.3 | 4.00 | 4.12 |
| 5 | 6 |  | 63928 | 57429 | 25599 | 26819 | 410 | 388 | 226 | 167 | 133 | 49 | 311 | 132 | 18.4 | 12.1 | 4.60 | 3.66 |
| 6 | 7 |  | 51994 | 58260 | 25638 | 26867 | 390 | 382 | 205 | 154 | 132 | 21 | 269 | 109 | 17.9 | 11.5 | 4.39 | 3.28 |
| 7 | 8 |  | 64236 | 64760 | 26940 | 28136 | 386 | 386 | 192 | 138 | 73 | 18 | 194 | 107 | 13.9 | 12.0 | 4.33 | 3.24 |
| 8 | 9 |  | 62253 | 52079 | 28347 | 26309 | 417 | 378 | 127 | 122 | 34 | 6 | 103 | 108 | 13.4 | 11.7 | 3.33 | 3.37 |
| 9 | 10 |  | 65687 | 56315 | 28181 | 26819 | 405 | 386 | 112 | 209 | 29 | 23 | 92 | 151 | 13.3 | 12.3 | 3.42 | 3.84 |
| 10 | 11 |  | 68392 | 49951 | 29281 | 26005 | 407 | 382 | 111 | 158 | 32 | 28 | 102 | 190 | 14.1 | 11.7 | 3.71 | 3.29 |
| 11 | 12 |  | 68624 | 57373 | 28179 | 25441 | 382 | 366 | 81 | 157 | 22 | 31 | 86 | 205 | 14.0 | 11.1 | 3.08 | 2.54 |
| 12 | 13 |  | 74411 | 64681 | 30146 | 28013 | 420 | 391 | 83 | 100 | 22 | 8 | 91 | 134 | 14.8 | 12.0 | 3.15 | 2.38 |
| 13 | 14 |  | 66182 | 65570 | 27756 | 31374 | 394 | 378 | 41 | 81 | 9 | ND | 83 | 113 | 13.7 | 14.1 | 1.72 | 2.16 |
| 14 | 15 |  | 71705 | 71243 | 30024 | 32582 | 402 | 381 | 50 | 86 | 14 | ND | 94 | 133 | 15.1 | 14.6 | 1.82 | 1.71 |
| 15 | 16 |  | 66924 | 63561 | 29696 | 29480 | 402 | 376 | 47 | 74 | 14 | ND | 98 | 113 | 14.9 | 13.1 | 2.13 | 1.57 |
| 16 | 17 |  | 64281 | 66501 | 30281 | 29558 | 383 | 382 | 53 | 73 | 14 | ND | 98 | 115 | 15.0 | 12.9 | 2.24 | 1.82 |
| 17 | 18 |  | 65680 | 65950 | 32640 | 29941 | 360 | 390 | 61 | 74 | 20 | ND | 127 | 114 | 16.5 | 13.1 | 1.78 | 2.46 |
| 18 | 19 |  | 67671 |  | 29341 |  | 389 |  | 45 |  | 10 |  | 97 |  | 14.2 |  | 1.40 |  |
| 19 | 20 |  | 67872 |  | 28945 |  | 369 |  | 47 |  | 10 |  | 101 |  | 14.2 |  | 1.42 |  |
| 20 | 21 |  | 69205 |  | 31031 |  | 373 |  | 45 |  | 11 |  | 107 |  | 14.3 |  | 1.93 |  |
| 21 | 22 |  | 59583 |  | 26374 |  | 356 |  | 38 |  | 8 |  | 94 |  | 11.8 |  | 2.38 |  |
| 22 | 23 |  | 53962 |  | 26517 |  | 345 |  | 38 |  | 7 |  | 97 |  | 11.6 |  | 2.59 |  |
| 23 | 24 |  | 58580 |  | 28207 |  | 359 |  | 36 |  | 12 |  | 91 |  | 12.4 |  | 2.63 |  |

| Site: | SR | Concentrations (µg/g dry sediment) | | | | | | | | | | | | | | | | |
| --- | --- | --- | --- | --- | --- | --- | --- | --- | --- | --- | --- | --- | --- | --- | --- | --- | --- | --- |
| Depth (cm) | | analyte | Al | | Fe | | Mn | | Zn | | Cu | | Cr | | Ni | | Mo | |
| top | bottom | core | 1 | 2 | 1 | 2 | 1 | 2 | 1 | 2 | 1 | 2 | 1 | 2 | 1 | 2 | 1 | 2 |
| 0 | 1 |  | 56815 | 60582 | 31718 | 27930 | 403 | 375 | 130 | 129 | 58 | 46 | 96 | 80 | 17.9 | 15.3 | 3.76 | 3.31 |
| 1 | 2 |  | 59426 | 55906 | 29143 | 27539 | 365 | 371 | 122 | 132 | 66 | 42 | 92 | 81 | 18.2 | 14.8 | 3.68 | 3.76 |
| 2 | 3 |  | 55944 | 58575 | 28932 | 30375 | 376 | 410 | 109 | 143 | 58 | 63 | 90 | 92 | 15.9 | 17.7 | 4.23 | 3.47 |
| 3 | 4 |  | 56703 | 56065 | 29644 | 28390 | 384 | 382 | 116 | 151 | 57 | 59 | 95 | 92 | 17.0 | 16.9 | 4.69 | 3.17 |
| 4 | 5 |  | 53695 | 60822 | 27413 | 28094 | 359 | 376 | 112 | 140 | 49 | 56 | 90 | 94 | 16.1 | 16.6 | 5.17 | 2.85 |
| 5 | 6 |  | 54070 | 53090 | 28867 | 26366 | 372 | 337 | 126 | 152 | 61 | 58 | 98 | 92 | 18.2 | 15.1 | 6.63 | 2.95 |
| 6 | 7 |  | 51207 | 63906 | 30191 | 28051 | 397 | 384 | 136 | 163 | 84 | 67 | 107 | 97 | 21.2 | 17.7 | 5.89 | 3.24 |
| 7 | 8 |  | 57113 | 59379 | 28995 | 28228 | 387 | 389 | 133 | 155 | 78 | 84 | 110 | 100 | 20.9 | 20.2 | 5.87 | 4.53 |
| 8 | 9 |  | 50971 | 61214 | 26467 | 29430 | 359 | 402 | 128 | 157 | 76 | 89 | 118 | 116 | 19.2 | 22.4 | 6.18 | 5.33 |
| 9 | 10 |  | 55658 | 61648 | 29051 | 28036 | 390 | 393 | 148 | 155 | 84 | 82 | 140 | 127 | 21.2 | 21.2 | 6.80 | 5.45 |
| 10 | 11 |  | 52179 | 60262 | 29536 | 25112 | 390 | 377 | 144 | 137 | 73 | 58 | 139 | 111 | 19.9 | 16.5 | 7.11 | 5.19 |
| 11 | 12 |  | 60900 | 58286 | 26553 | 23471 | 364 | 348 | 133 | 140 | 57 | 26 | 128 | 115 | 16.9 | 11.9 | 6.82 | 5.39 |
| 12 | 13 |  | 59836 | 65470 | 25834 | 25297 | 353 | 371 | 130 | 157 | 30 | 21 | 121 | 131 | 14.8 | 12.6 | 6.75 | 4.56 |
| 13 | 14 |  | 63976 | 61481 | 26416 | 25812 | 376 | 371 | 125 | 160 | 28 | 26 | 109 | 125 | 14.4 | 11.3 | 6.18 | 3.44 |
| 14 | 15 |  | 60587 | 50346 | 24536 | 23813 | 363 | 338 | 139 | 135 | 23 | 5 | 111 | 105 | 12.5 | 10.4 | 4.54 | 3.07 |
| 15 | 16 |  | 69803 | 53209 | 28912 | 24766 | 405 | 338 | 119 | 117 | 4 | ND | 87 | 75 | 12.9 | 11.2 | 3.23 | 2.43 |
| 16 | 17 |  | 67843 | 56944 | 27656 | 25893 | 375 | 349 | 97 | 112 | ND | ND | 68 | 71 | 12.2 | 10.9 | 2.88 | 2.13 |
| 17 | 18 |  | 67219 | 59167 | 28369 | 26515 | 381 | 352 | 93 | 121 | ND | 7 | 65 | 91 | 11.5 | 12.1 | 2.62 | 2.84 |
| 18 | 19 |  | 68747 | 62717 | 28337 | 29302 | 374 | 381 | 106 | 115 | ND | ND | 72 | 80 | 12.1 | 13.1 | 2.81 | 2.85 |
| 19 | 20 |  | 65850 | 54055 | 24416 | 26227 | 336 | 350 | 77 | 93 | ND | ND | 51 | 61 | 9.7 | 10.0 | 3.21 | 2.67 |
| 20 | 21 |  | 56990 | 56028 | 25022 | 25447 | 369 | 359 | 82 | 70 | ND | ND | 60 | 49 | 11.6 | 9.2 | 2.96 | 2.42 |
| 21 | 22 |  | 56130 | 53429 | 26484 | 23504 | 368 | 340 | 76 | 51 | ND | ND | 55 | 46 | 11.7 | 8.8 | 2.90 | 2.89 |
| 22 | 23 |  | 58183 | 56658 | 26207 | 22785 | 369 | 331 | 60 | 38 | ND | ND | 47 | 39 | 10.8 | 8.2 | 3.07 | 3.14 |
| 23 | 24 |  | 62692 | 61400 | 24788 | 24412 | 360 | 361 | 43 | 36 | ND | ND | 38 | 37 | 8.9 | 10.2 | 3.42 | 3.53 |
| 24 | 25 |  | 65222 | 59517 | 25944 | 23870 | 378 | 360 | 35 | 34 | ND | ND | 35 | 36 | 9.6 | 9.2 | 3.90 | 3.85 |
| 25 | 26 |  | 65370 | 61910 | 25083 | 26171 | 376 | 374 | 34 | 44 | ND | ND | 31 | 37 | 8.9 | 9.2 | 4.86 | 4.35 |
| 26 | 27 |  |  | 59880 |  | 22338 |  | 323 |  | 45 |  | ND |  | 39 |  | 8.2 |  | 3.44 |
| 27 | 28 |  |  | 61712 |  | 24676 |  | 346 |  | 33 |  | ND |  | 26 |  | 8.8 |  | 4.67 |
| 28 | 29 |  |  | 60365 |  | 25340 |  | 338 |  | 26 |  | ND |  | 21 |  | 8.9 |  | 5.26 |
| 29 | 30 |  |  | 62931 |  | 23233 |  | 333 |  | 23 |  | ND |  | 20 |  | 8.5 |  | 4.83 |
| 30 | 31 |  |  | 59826 |  | 21927 |  | 332 |  | 20 |  | ND |  | 14 |  | 6.8 |  | 4.97 |

| Site: | PP | Concentrations (µg/g dry sediment) | | | | | | | | | | | | | | | | |
| --- | --- | --- | --- | --- | --- | --- | --- | --- | --- | --- | --- | --- | --- | --- | --- | --- | --- | --- |
|  |  | analyte | Al | | Fe | | Mn | | Zn | | Cu | | Cr | | Ni | | Mo | |
| top | bottom | core | 2 | 3 | 2 | 3 | 2 | 3 | 2 | 3 | 2 | 3 | 2 | 3 | 2 | 3 | 2 | 3 |
| 0 | 1 |  | 50575 | 51581 | 31493 | 30717 | 385 | 370 | 116 | 109 | 60 | 41 | 93 | 80 | 22.1 | 14.6 | 1.44 | 1.66 |
| 1 | 2 |  | 51506 | 49531 | 32250 | 30138 | 374 | 351 | 116 | 100 | 60 | 37 | 93 | 76 | 21.0 | 13.2 | 1.68 | 1.62 |
| 2 | 3 |  | 50249 | 54129 | 31015 | 33090 | 376 | 381 | 121 | 111 | 63 | 43 | 95 | 83 | 21.8 | 15.1 | 2.01 | 2.06 |
| 3 | 4 |  | 50867 | 54925 | 30984 | 34181 | 369 | 396 | 123 | 118 | 62 | 47 | 99 | 87 | 21.5 | 16.4 | 2.23 | 2.24 |
| 4 | 5 |  | 53277 | 54673 | 31575 | 35465 | 378 | 401 | 130 | 119 | 64 | 50 | 100 | 87 | 22.2 | 16.3 | 2.61 | 2.65 |
| 5 | 6 |  | 57047 | 49971 | 33874 | 33445 | 404 | 402 | 133 | 122 | 74 | 51 | 102 | 89 | 24.7 | 16.4 | 2.72 | 2.99 |
| 6 | 7 |  | 56865 | 47744 | 34610 | 32675 | 416 | 397 | 133 | 122 | 79 | 51 | 149 | 86 | 30.6 | 16.4 | 3.35 | 3.32 |
| 7 | 8 |  | 57330 | 47794 | 32767 | 32836 | 396 | 400 | 135 | 132 | 77 | 62 | 106 | 92 | 25.1 | 18.1 | 3.34 | 3.90 |
| 8 | 9 |  | 56770 | 56486 | 33697 | 35017 | 410 | 439 | 148 | 155 | 79 | 81 | 109 | 110 | 24.2 | 22.2 | 3.39 | 4.31 |
| 9 | 10 |  | 55399 | 54755 | 33882 | 35013 | 414 | 454 | 134 | 174 | 107 | 106 | 107 | 120 | 24.2 | 22.7 | 3.46 | 3.82 |
| 10 | 11 |  | 52871 | 60614 | 32876 | 35612 | 415 | 450 | 144 | 161 | 96 | 107 | 117 | 117 | 25.0 | 19.1 | 3.59 | 3.09 |
| 11 | 12 |  | 54118 | 48596 | 34264 | 35571 | 421 | 444 | 160 | 170 | 119 | 117 | 131 | 122 | 24.8 | 19.8 | 3.81 | 3.39 |
| 12 | 13 |  | 57029 | 57750 | 33391 | 34211 | 417 | 425 | 170 | 166 | 130 | 117 | 132 | 121 | 25.6 | 19.4 | 4.48 | 3.69 |
| 13 | 14 |  | 52616 | 60935 | 35468 | 36352 | 429 | 438 | 168 | 178 | 122 | 112 | 121 | 128 | 19.1 | 20.0 | 3.67 | 5.28 |
| 14 | 15 |  | 51920 | 60380 | 35796 | 34000 | 431 | 422 | 165 | 168 | 120 | 101 | 125 | 119 | 18.9 | 17.9 | 2.69 | 4.52 |
| 15 | 16 |  | 53280 | 59943 | 35808 | 33608 | 422 | 426 | 146 | 156 | 103 | 90 | 115 | 117 | 16.0 | 16.7 | 2.49 | 2.94 |
| 16 | 17 |  | 57026 | 62290 | 36669 | 34985 | 449 | 434 | 158 | 153 | 113 | 94 | 130 | 124 | 16.3 | 16.0 | 2.76 | 2.77 |
| 17 | 18 |  | 57585 | 57350 | 35548 | 33557 | 452 | 416 | 142 | 145 | 97 | 87 | 125 | 123 | 16.6 | 15.0 | 2.81 | 2.68 |
| 18 | 19 |  | 50462 | 55870 | 33792 | 30798 | 437 | 386 | 154 | 118 | 116 | 58 | 125 | 100 | 15.1 | 12.3 | 2.54 | 3.24 |
| 19 | 20 |  | 56326 | 55275 | 34223 | 36513 | 444 | 449 | 151 | 137 | 125 | 38 | 138 | 107 | 14.6 | 22.4 | 2.46 | 2.69 |
| 20 | 21 |  | 59556 | 60164 | 34849 | 38956 | 442 | 462 | 162 | 137 | 126 | 22 | 143 | 112 | 15.3 | 25.2 | 2.17 | 2.96 |
| 21 | 22 |  | 52615 | 51681 | 34204 | 35192 | 409 | 415 | 167 | 115 | 111 | 21 | 129 | 97 | 16.5 | 22.3 | 1.91 | 3.93 |
| 22 | 23 |  | 52288 | 54968 | 35207 | 37072 | 414 | 425 | 142 | 96 | 71 | 12 | 115 | 88 | 14.9 | 23.0 | 2.19 | 4.49 |
| 23 | 24 |  | 57431 | 47112 | 36492 | 34405 | 420 | 405 | 104 | 86 | 34 | 5 | 97 | 80 | 15.8 | 23.2 | 3.09 | 4.30 |
| 24 | 25 |  | 55633 | 51144 | 36931 | 37086 | 424 | 419 | 88 | 85 | 18 | 4 | 91 | 81 | 17.3 | 23.3 | 3.13 | 4.55 |
| 25 | 26 |  | 62717 | 45797 | 36800 | 34495 | 415 | 394 | 68 | 82 | 6 | ND | 80 | 77 | 15.5 | 23.0 | 2.88 | 1.68 |
| 26 | 27 |  | 55368 | 35740 | 34606 | 24182 | 402 | 266 | 64 | 57 | 3 | ND | 73 | 52 | 17.0 | 15.8 | 3.25 | 1.12 |
| 27 | 28 |  | 58903 | 46035 | 35357 | 33285 | 407 | 389 | 64 | 80 | ND | ND | 72 | 72 | 16.6 | 22.5 | 3.39 | 1.59 |
| 28 | 29 |  | 57258 | 48610 | 33540 | 36203 | 384 | 408 | 57 | 76 | ND | ND | 65 | 71 | 15.3 | 22.5 | 2.89 | 2.02 |
| 29 | 30 |  | 61066 | 55028 | 33751 | 37138 | 389 | 419 | 56 | 81 | ND | ND | 65 | 75 | 15.3 | 24.1 | 2.60 | 2.60 |
| 30 | 31 |  | 59237 | 56130 | 33396 | 35785 | 388 | 411 | 51 | 76 | ND | ND | 63 | 72 | 14.1 | 23.0 | 2.44 | 2.68 |
| 31 | 32 |  | 64302 | 54527 | 35478 | 34861 | 398 | 397 | 53 | 79 | ND | ND | 63 | 74 | 14.5 | 23.7 | 2.50 | 2.41 |
| 32 | 33 |  | 64459 | 60905 | 34723 | 36336 | 390 | 392 | 54 | 76 | ND | ND | 63 | 73 | 16.7 | 23.5 | 3.14 | 2.84 |
| 33 | 34 |  | 53813 | 55963 | 31089 | 34229 | 358 | 390 | 51 | 76 | ND | ND | 58 | 71 | 15.6 | 24.2 | 2.75 | 2.78 |
| 34 | 35 |  |  | 59988 |  | 32927 |  | 384 |  | 70 |  | ND |  | 67 |  | 21.9 |  | 2.74 |

| Site: | QP | Concentrations (µg/g dry sediment) | | | | | | | | | | | | | | | | |
| --- | --- | --- | --- | --- | --- | --- | --- | --- | --- | --- | --- | --- | --- | --- | --- | --- | --- | --- |
|  |  | analyte | Al | | Fe | | Mn | | Zn | | Cu | | Cr | | Ni | | Mo | |
| top | bottom | core | 1 | 3 | 1 | 3 | 1 | 3 | 1 | 3 | 1 | 3 | 1 | 3 | 1 | 3 | 1 | 3 |
| 0 | 1 |  | 49114 | 55098 | 27414 | 26982 | 371 | 373 | 93 | 80 | 31 | 30 | 68 | 69 | 18.4 | 17.4 | 1.49 | 1.37 |
| 1 | 2 |  | 53740 | 52587 | 27747 | 27067 | 370 | 370 | 92 | 82 | 35 | 31 | 72 | 68 | 19.4 | 17.9 | 1.73 | 1.99 |
| 2 | 3 |  | 51113 | 54838 | 27953 | 27736 | 373 | 370 | 90 | 84 | 37 | 32 | 71 | 74 | 19.5 | 18.4 | 2.22 | 1.65 |
| 3 | 4 |  |  | 48414 |  | 26129 |  | 351 |  | 81 |  | 31 |  | 67 |  | 17.4 |  | 1.58 |
|  | 5 |  | 53172 |  | 29029 |  | 377 |  | 86 |  | 33 |  | 69 |  | 20.4 |  | 2.76 |  |
| 4 | 5 |  |  | 47847 |  | 25004 |  | 349 |  | 78 |  | 31 |  | 63 |  | 17.2 |  | 1.45 |
| 5 | 6 |  | 52434 | 48147 | 29334 | 26623 | 386 | 360 | 84 | 77 | 27 | 30 | 65 | 64 | 21.9 | 17.7 | 3.12 | 1.51 |
| 6 | 7 |  | 52146 | 49528 | 29420 | 28396 | 388 | 388 | 84 | 98 | 28 | 28 | 63 | 59 | 21.9 | 21.5 | 2.97 | 2.38 |
| 7 | 8 |  | 54003 | 53073 | 30034 | 30148 | 367 | 384 | 88 | 80 | 28 | 19 | 63 | 61 | 20.4 | 21.4 | 3.25 | 3.45 |
| 8 | 9 |  | 56237 | 54037 | 29162 | 29811 | 389 | 385 | 90 | 80 | 24 | 21 | 69 | 63 | 25.2 | 21.2 | 4.33 | 3.40 |
| 9 | 10 |  | 54013 | 51203 | 28954 | 29073 | 386 | 379 | 90 | 80 | 29 | 24 | 67 | 68 | 22.4 | 20.8 | 3.55 | 3.13 |
| 10 | 11 |  | 52004 | 52695 | 30027 | 30312 | 399 | 408 | 94 | 71 | 27 | 18 | 64 | 65 | 21.9 | 21.3 | 3.19 | 3.12 |
| 11 | 12 |  | 56373 | 54697 | 30563 | 31402 | 411 | 407 | 96 | 78 | 28 | 18 | 66 | 62 | 24.1 | 20.7 | 3.55 | 4.32 |
| 12 | 13 |  | 58821 | 57583 | 31496 | 32109 | 406 | 410 | 84 | 64 | 25 | 17 | 67 | 64 | 22.9 | 21.2 | 5.04 | 4.87 |
| 13 | 14 |  | 58578 | 59568 | 31794 | 32714 | 419 | 421 | 78 | 56 | 23 | 10 | 68 | 61 | 21.2 | 20.9 | 5.01 | 4.21 |
| 14 | 15 |  | 53922 | 63929 | 29837 | 34754 | 396 | 446 | 71 | 59 | 22 | 9 | 65 | 69 | 19.6 | 22.3 | 4.42 | 4.29 |
| 15 | 16 |  | 56941 | 63267 | 31295 | 35128 | 408 | 448 | 63 | 59 | 17 | 10 | 64 | 65 | 19.7 | 22.1 | 3.92 | 3.82 |
| 16 | 17 |  | 58271 | 63049 | 30590 | 34805 | 402 | 436 | 63 | 57 | 16 | 9 | 59 | 66 | 19.3 | 21.4 | 3.64 | 3.77 |
| 17 | 18 |  | 58430 | 61608 | 30637 | 33955 | 410 | 420 | 63 | 57 | 19 | 8 | 63 | 62 | 19.6 | 21.1 | 3.05 | 3.87 |
| 18 | 19 |  | 55597 | 63823 | 29797 | 34953 | 399 | 433 | 64 | 56 | 18 | 8 | 61 | 64 | 19.0 | 22.2 | 2.90 | 3.72 |
| 19 | 20 |  | 56598 | 64107 | 30558 | 35676 | 404 | 446 | 65 | 56 | 17 | 9 | 63 | 70 | 18.9 | 25.9 | 2.56 | 4.04 |
| 20 | 21 |  | 73463 | 59486 | 38326 | 33323 | 489 | 429 | 72 | 59 | 15 | 8 | 74 | 59 | 24.0 | 21.7 | 4.06 | 3.65 |
| 21 | 22 |  | 58403 | 61908 | 31209 | 32487 | 407 | 419 | 58 | 57 | 14 | 9 | 59 | 61 | 19.5 | 20.6 | 3.18 | 3.65 |
| 22 | 23 |  | 57584 | 70591 | 32004 | 34077 | 419 | 443 | 64 | 60 | 15 | 12 | 61 | 65 | 21.6 | 23.3 | 3.20 | 3.86 |
| 23 | 24 |  | 58760 | 52332 | 33247 | 30959 | 427 | 413 | 58 | 59 | 11 | 12 | 61 | 56 | 19.2 | 20.3 | 3.39 | 3.72 |
| 24 | 25 |  | 57733 | 55618 | 32733 | 31689 | 425 | 415 | 57 | 63 | 11 | 13 | 61 | 62 | 20.8 | 20.4 | 3.30 | 3.55 |
| 25 | 26 |  | 59087 | 54939 | 31848 | 31614 | 408 | 413 | 57 | 57 | 9 | 12 | 63 | 61 | 19.4 | 20.3 | 3.60 | 3.41 |
| 26 | 27 |  | 53915 | 48651 | 30171 | 29583 | 398 | 392 | 53 | 48 | 11 | 8 | 57 | 54 | 18.7 | 19.3 | 3.13 | 3.39 |
| 27 | 28 |  | 55212 | 54161 | 30722 | 31269 | 393 | 410 | 55 | 59 | 11 | 8 | 60 | 62 | 19.1 | 20.2 | 3.62 | 3.72 |
| 28 | 29 |  | 59886 | 55690 | 31690 | 33351 | 407 | 419 | 54 | 58 | 8 | 9 | 63 | 64 | 20.1 | 21.4 | 3.78 | 3.80 |
| 29 | 30 |  | 54796 | 54060 | 30655 | 34305 | 396 | 433 | 50 | 57 | 10 | 8 | 57 | 67 | 18.9 | 22.1 | 3.81 | 3.93 |
| 30 | 31 |  | 52582 |  | 30754 |  | 403 |  | 51 |  | 8 |  | 60 |  | 19.5 |  | 4.01 |  |
| 31 | 32 |  | 58826 |  | 31768 |  | 407 |  | 58 |  | 9 |  | 63 |  | 19.6 |  | 4.00 |  |
| 32 | 33 |  | 54992 |  | 30355 |  | 397 |  | 51 |  | 8 |  | 58 |  | 18.3 |  | 3.63 |  |
| 33 | 34 |  | 64298 |  | 32184 |  | 419 |  | 52 |  | 9 |  | 61 |  | 19.2 |  | 3.41 |  |
| 34 | 35 |  | 61637 |  | 34691 |  | 439 |  | 59 |  | 8 |  | 66 |  | 20.5 |  | 3.41 |  |
| 35 | 36 |  | 61875 |  | 35833 |  | 448 |  | 63 |  | 10 |  | 66 |  | 23.2 |  | 3.81 |  |
| 36 | 37 |  | 64470 |  | 36169 |  | 452 |  | 62 |  | 9 |  | 67 |  | 24.0 |  | 3.94 |  |
| 37 | 38 |  | 62314 |  | 36060 |  | 456 |  | 63 |  | 9 |  | 72 |  | 23.1 |  | 4.37 |  |

Table S5. Carbon and nitrogen concentrations, isotopic ratios and C/N in Narragansett Bay sediments

| Site | BR |  |  |  |  |  |  |  |  |  |  |  |
| --- | --- | --- | --- | --- | --- | --- | --- | --- | --- | --- | --- | --- |
| Depth (cm) | |  | % C | | δ^13^C | | % N | | δ^15^N | | C/N | |
| Top | bottom | Core | 2 | 3 | 2 | 3 | 2 | 3 | 2 | 3 | 2 | 3 |
| 0 | 1 |  |  | 3.58 |  | -21.2 |  | 0.34 |  | 7.6 |  | 10.4 |
|  | 2 |  | 3.66 |  | -21.1 |  | 0.13 |  | 6.8 |  | 28.3 |  |
| 1 | 2 |  |  | 3.57 |  | -21.0 |  | 0.36 |  | 7.9 |  | 9.9 |
| 2 | 3 |  | 3.64 | 3.61 | -21.1 | -20.3 | 0.37 | 0.35 | 7.6 | 7.9 | 9.9 | 10.3 |
| 3 | 4 |  | 3.79 | 3.62 | -20.7 | -20.8 | 0.38 | 0.36 | 7.8 | 7.7 | 10.0 | 10.2 |
| 4 | 5 |  | 3.68 | 3.69 | -21.0 | -20.5 | 0.37 | 0.36 | 7.7 | 7.9 | 9.9 | 10.2 |
| 5 | 6 |  | 3.59 | 3.85 | -20.7 | -20.4 | 0.35 | 0.37 | 7.5 | 7.8 | 10.2 | 10.3 |
| 6 | 7 |  | 3.53 | 3.85 | -20.6 | -20.5 | 0.33 | 0.38 | 7.4 | 7.5 | 10.6 | 10.1 |
| 7 | 8 |  | 3.66 | 3.47 | -20.9 | -20.4 | 0.34 | 0.32 | 6.8 | 7.0 | 10.9 | 11.0 |
| 8 | 9 |  | 3.41 | 3.60 | -21.3 | -20.8 | 0.32 | 0.33 | 6.9 | 7.2 | 10.7 | 11.1 |
| 9 | 10 |  | 3.57 | 3.48 | -21.0 | -20.9 | 0.33 | 0.32 | 6.8 | 7.0 | 10.9 | 10.9 |
| 10 | 11 |  | 3.75 | 3.61 | -21.1 | -20.5 | 0.33 | 0.32 | 6.8 | 7.1 | 11.3 | 11.1 |
| 11 | 12 |  | 3.37 | 3.48 | -20.9 | -21.1 | 0.29 | 0.31 | 7.2 | 6.7 | 11.5 | 11.4 |
| 12 | 13 |  | 3.45 | 3.44 | -21.1 | -20.8 | 0.30 | 0.30 | 6.1 | 6.8 | 11.7 | 11.4 |
| 13 | 14 |  | 3.74 | 3.22 | -21.4 | -21.1 | 0.34 | 0.28 | 6.6 | 6.7 | 11.0 | 11.6 |
| 14 | 15 |  | 3.65 | 3.40 | -21.3 | -21.3 | 0.33 | 0.28 | 6.9 | 7.0 | 11.0 | 12.0 |
| 15 | 16 |  | 3.60 | 3.06 | -21.3 | -21.3 | 0.32 | 0.26 | 7.3 | 6.7 | 11.3 | 11.8 |
| 16 | 17 |  | 3.72 | 3.40 | -21.5 | -20.1 | 0.34 | 0.27 | 6.6 | 7.0 | 10.9 | 12.6 |
| 17 | 18 |  | 3.57 | 3.51 | -21.3 | -21.6 | 0.28 | 0.30 | 6.5 | 7.0 | 12.9 | 11.9 |
| 18 | 19 |  | 3.60 | 3.64 | -21.4 | -21.1 | 0.33 | 0.29 | 6.8 | 6.7 | 11.0 | 12.6 |
| 19 | 20 |  | 3.22 | 3.94 | -21.5 | -21.9 | 0.28 | 0.32 | 7.1 | 7.2 | 11.5 | 12.5 |
| 20 | 21 |  | 3.14 | 4.03 | -21.7 | -21.7 | 0.27 | 0.33 | 7.1 | 7.0 | 11.5 | 12.2 |
| 21 | 22 |  | 3.41 | 4.13 | -21.3 | -21.3 | 0.30 | 0.34 | 6.9 | 7.2 | 11.5 | 12.3 |
| 22 | 23 |  | 3.33 | 4.23 | -21.6 | -21.9 | 0.29 | 0.34 | 7.2 | 7.3 | 11.3 | 12.5 |
| 23 | 24 |  | 3.61 | 4.06 | -21.7 | -22.0 | 0.31 | 0.32 | 7.6 | 7.1 | 11.5 | 12.8 |
| 24 | 25 |  | 3.63 | 4.01 | -21.5 | -22.1 | 0.31 | 0.33 | 7.4 | 7.3 | 11.8 | 12.1 |
| 25 | 26 |  | 3.74 | 4.25 | -21.4 | -22.1 | 0.32 | 0.35 | 7.6 | 7.5 | 11.6 | 12.0 |
| 26 | 27 |  |  | 4.33 |  | -22.0 |  | 0.34 |  | 7.5 |  | 12.6 |
| 27 | 28 |  |  | 4.26 |  | -21.8 |  | 0.35 |  | 7.3 |  | 12.3 |
| 28 | 29 |  |  | 4.21 |  | -21.8 |  | 0.34 |  | 7.4 |  | 12.2 |
| 29 | 30 |  |  | 4.23 |  | -21.6 |  | 0.34 |  | 7.5 |  | 12.5 |
| 30 | 31 |  |  | 4.16 |  | -21.6 |  | 0.34 |  | 7.5 |  | 12.3 |
| 31 | 32 |  |  | 4.28 |  | -21.4 |  | 0.34 |  | 7.6 |  | 12.6 |

| Site | CP |  |  |  |  |  |  |  |  |  |  |  |
| --- | --- | --- | --- | --- | --- | --- | --- | --- | --- | --- | --- | --- |
| Depth (cm) | |  | % C | | δ^13^C | | % N | | δ^15^N | | C/N | |
| Top | Bottom | Core | 1 | 2 | 1 | 2 | 1 | 2 | 1 | 2 | 1 | 2 |
| 0 | 1 |  | 3.36 | 3.61 | -19.8 | -19.5 | 0.34 | 0.36 | 7.7 | 7.9 | 9.9 | 10.1 |
| 1 | 2 |  | 3.29 | 3.30 | -20.0 | -18.9 | 0.33 | 0.32 | 7.4 | 7.5 | 9.9 | 10.4 |
| 2 | 3 |  | 3.43 | 3.36 | -19.5 | -20.0 | 0.34 | 0.34 | 7.5 | 7.5 | 10.1 | 10.0 |
| 3 | 4 |  | 3.73 | 3.29 | -18.9 | -20.2 | 0.36 | 0.34 | 7.5 | 7.5 | 10.5 | 9.7 |
| 4 | 5 |  | 3.53 | 3.22 | -19.8 | -20.3 | 0.37 | 0.33 | 7.4 | 7.6 | 9.6 | 9.6 |
| 5 | 6 |  | 2.83 | 3.01 | -19.6 | -19.6 | 0.27 | 0.29 | 7.9 | 7.9 | 10.5 | 10.3 |
| 6 | 7 |  | 2.84 | 2.87 | -19.0 | -18.2 | 0.26 | 0.26 | 8.1 | 7.9 | 11.0 | 10.9 |
| 7 | 8 |  | 2.93 | 3.03 | -18.5 | -18.7 | 0.26 | 0.26 | 7.4 | 7.5 | 11.1 | 11.5 |
| 8 | 9 |  | 3.44 | 3.27 | -19.5 | -19.4 | 0.31 | 0.33 | 7.1 | 7.4 | 11.3 | 10.1 |
| 9 | 10 |  | 3.22 | 3.20 | -20.1 | -19.4 | 0.29 | 0.29 | 6.8 | 7.3 | 10.9 | 10.9 |
| 10 | 11 |  | 3.14 | 3.23 | -19.6 | -19.5 | 0.27 | 0.29 | 6.7 | 7.0 | 11.5 | 11.0 |
| 11 | 12 |  | 3.21 | 3.26 | -19.7 | -20.2 | 0.28 | 0.30 | 6.8 | 6.8 | 11.6 | 11.0 |
| 12 | 13 |  | 3.23 | 3.20 | -19.5 | -20.0 | 0.27 | 0.29 | 6.7 | 6.9 | 11.9 | 11.1 |
| 13 | 14 |  | 3.12 | 3.25 | -20.3 | -20.3 | 0.28 | 0.29 | 6.8 | 7.0 | 11.2 | 11.2 |
| 14 | 15 |  | 3.39 | 3.38 | -20.5 | -20.4 | 0.30 | 0.30 | 6.8 | 6.8 | 11.4 | 11.3 |
| 15 | 16 |  | 3.49 | 3.34 | -20.5 | -20.6 | 0.31 | 0.30 | 6.9 | 6.9 | 11.3 | 11.2 |
| 16 | 17 |  | 3.72 | 3.52 | -20.6 | -20.1 | 0.31 | 0.30 | 7.0 | 7.2 | 12.1 | 11.7 |
| 17 | 18 |  | 3.51 | 3.41 | -20.8 | -20.2 | 0.30 | 0.29 | 7.2 | 7.3 | 11.6 | 11.8 |
| 18 | 19 |  | 3.66 | 3.39 | -20.4 | -20.3 | 0.32 | 0.29 | 7.2 | 7.3 | 11.4 | 11.6 |
| 19 | 20 |  | 3.62 | 3.11 | -20.6 | -19.5 | 0.31 | 0.26 | 7.1 | 7.1 | 11.7 | 12.0 |
| 20 | 21 |  | 3.63 | 3.14 | -20.0 | -19.5 | 0.30 | 0.26 | 7.2 | 7.4 | 12.1 | 12.3 |
| 21 | 22 |  | 3.51 | 3.28 | -20.0 | -19.6 | 0.29 | 0.26 | 7.4 | 7.4 | 12.3 | 12.4 |
| 22 | 23 |  | 3.64 | 3.36 | -19.6 | -19.6 | 0.32 | 0.28 | 7.4 | 7.7 | 11.5 | 12.0 |
| 23 | 24 |  | 3.66 | 3.36 | -20.2 | -20.0 | 0.31 | 0.29 | 7.5 | 7.7 | 11.8 | 11.6 |
| 24 | 25 |  | 3.78 | 3.57 | -19.8 | -20.5 | 0.31 | 0.31 | 7.7 | 7.7 | 12.1 | 11.6 |
| 25 | 26 |  | 3.56 | 3.43 | -19.6 | -20.5 | 0.29 | 0.30 | 7.7 | 7.5 | 12.3 | 11.6 |
| 26 | 27 |  | 3.85 | 3.09 | -20.7 | -20.6 | 0.34 | 0.27 | 7.9 | 7.6 | 11.4 | 11.3 |
| 27 | 28 |  | 3.76 | 2.94 | -20.8 | -20.7 | 0.33 | 0.26 | 8.0 | 7.6 | 11.3 | 11.3 |
| 28 | 29 |  | 3.42 | 3.12 | -21.2 | -21.0 | 0.31 | 0.27 | 7.4 | 7.5 | 11.0 | 11.4 |
| 29 | 30 |  | 3.25 | 2.71 | -21.2 | -18.8 | 0.28 | 0.22 | 7.3 | 8.0 | 11.7 | 12.2 |
| 30 | 31 |  | 3.15 | 1.36 | -20.3 | -17.1 | 0.27 | 0.10 | 8.1 | 7.9 | 11.5 | 13.3 |
| 31 | 32 |  |  | 3.54 |  | -19.4 |  | 0.30 |  | 7.7 |  | 11.9 |

| Site | NP |  |  |  |  |  |  |  |  |  |  |  |
| --- | --- | --- | --- | --- | --- | --- | --- | --- | --- | --- | --- | --- |
| Depth (cm) | |  | % C | | δ^13^C | | % N | | δ^15^N | | C/N | |
| Top | bottom | Core | 1 | 2 | 1 | 2 | 1 | 2 | 1 | 2 | 1 | 2 |
| 0 | 1 |  | 3.52 | 3.73 | -18.2 | -18.5 | 0.36 | 0.39 | 8.6 | 8.6 | 9.9 | 9.6 |
| 1 | 2 |  | 3.58 | 3.56 | -18.4 | -18.6 | 0.37 | 0.37 | 8.4 | 8.4 | 9.7 | 9.5 |
| 2 | 3 |  | 3.52 | 3.60 | -18.0 | -18.2 | 0.35 | 0.38 | 8.2 | 8.7 | 10.2 | 9.6 |
| 3 | 4 |  | 3.48 | 3.77 | -17.7 | -17.3 | 0.33 | 0.36 | 8.3 | 8.1 | 10.5 | 10.6 |
| 4 | 5 |  | 3.43 | 3.83 | -17.6 | -17.5 | 0.32 | 0.37 | 8.2 | 8.0 | 10.6 | 10.2 |
| 5 | 6 |  | 3.11 | 3.79 | -17.9 | -19.2 | 0.29 | 0.39 | 8.5 | 8.2 | 10.6 | 9.7 |
| 6 | 7 |  | 3.27 | 3.63 | -18.2 | -18.8 | 0.31 | 0.38 | 8.2 | 8.0 | 10.4 | 9.6 |
| 7 | 8 |  | 3.13 | 3.29 | -18.6 | -17.4 | 0.29 | 0.31 | 7.9 | 7.9 | 11.0 | 10.7 |
| 8 | 9 |  | 3.24 | 3.05 | -17.3 | -16.4 | 0.28 | 0.25 | 7.9 | 8.0 | 11.7 | 12.0 |
| 9 | 10 |  | 3.30 | 3.11 | -18.3 | -17.3 | 0.30 | 0.28 | 7.9 | 8.1 | 11.0 | 11.0 |
| 10 | 11 |  | 3.51 | 3.10 | -19.7 | -18.7 | 0.29 | 0.27 | 8.1 | 8.0 | 12.0 | 11.6 |
| 11 | 12 |  | 3.21 | 3.10 | -19.1 | -19.3 | 0.28 | 0.28 | 8.1 | 8.2 | 11.6 | 11.2 |
| 12 | 13 |  |  | 3.22 |  | -19.9 |  | 0.29 |  | 8.0 |  | 11.2 |
| 13 | 14 |  |  | 2.99 |  | -19.3 |  | 0.26 |  | 8.1 |  | 11.5 |
| 14 | 15 |  |  | 3.32 |  | -19.2 |  | 0.30 |  | 8.1 |  | 11.2 |
| 15 | 16 |  |  | 3.45 |  | -19.5 |  | 0.30 |  | 8.1 |  | 11.4 |
| 16 | 17 |  |  | 3.31 |  | -19.1 |  | 0.29 |  | 8.2 |  | 11.3 |
| 17 | 18 |  |  | 3.79 |  | -18.2 |  | 0.33 |  | 8.5 |  | 11.7 |
| 18 | 19 |  |  | 3.47 |  | -19.3 |  | 0.31 |  | 8.3 |  | 11.3 |
| 20 | 21 |  |  | 3.07 |  | -18.1 |  | 0.26 |  | 8.2 |  | 11.8 |
| 21 | 22 |  |  | 2.30 |  | -17.9 |  | 0.19 |  | 7.9 |  | 12.1 |
| 22 | 23 |  |  | 1.92 |  | -18.6 |  | 0.16 |  | 8.0 |  | 11.7 |
| 23 | 24 |  |  | 1.88 |  | -17.3 |  | 0.15 |  | 7.7 |  | 12.5 |
| 24 | 25 |  |  | 1.75 |  | -15.6 |  | 0.13 |  | 7.5 |  | 13.0 |
| 25 | 26 |  |  | 1.83 |  | -16.5 |  | 0.15 |  | 7.3 |  | 12.6 |
| 26 | 27 |  |  | 1.89 |  | -16.0 |  | 0.14 |  | 7.4 |  | 13.2 |
| 27 | 28 |  |  | 1.88 |  | -15.5 |  | 0.14 |  | 7.5 |  | 13.7 |
| 28 | 29 |  |  | 1.76 |  | -16.0 |  | 0.13 |  | 7.5 |  | 13.3 |
| 29 | 30 |  |  | 1.72 |  | -15.7 |  | 0.13 |  | 7.2 |  | 13.2 |

| Site | GB |  |  |  |  |  |  |  |  |  |  |  |
| --- | --- | --- | --- | --- | --- | --- | --- | --- | --- | --- | --- | --- |
| Depth (cm) | |  | % C | | δ^13^C | | % N | | δ ^15^N | | C/N | |
| Top | bottom | Core | 1 | 3 | 1 | 3 | 1 | 3 | 1 | 3 | 1 | 3 |
| 0 | 1 |  | 4.32 | 3.44 | -16.7 | -17.9 | 0.40 | 0.28 | 9.8 | 8.3 | 10.9 | 12.5 |
| 1 | 2 |  | 4.28 | 3.50 | -17.0 | -18.5 | 0.38 | 0.31 | 9.2 | 8.2 | 11.2 | 11.4 |
| 2 | 3 |  | 2.96 | 3.52 | -18.3 | -18.7 | 0.26 | 0.29 | 8.4 | 7.9 | 11.4 | 12.2 |
| 3 | 4 |  | 3.66 | 3.20 | -17.6 | -19.9 | 0.31 | 0.25 | 8.9 | 7.7 | 11.8 | 13.0 |
| 4 | 5 |  | 3.57 | 2.96 | -19.2 | -20.5 | 0.30 | 0.22 | 9.2 | 7.5 | 11.8 | 13.5 |
| 5 | 6 |  | 3.58 | 2.61 | -20.6 | -20.1 | 0.32 | 0.21 | 8.2 | 7.5 | 11.3 | 12.7 |
| 6 | 7 |  | 3.33 | 2.41 | -21.0 | -20.3 | 0.31 | 0.19 | 8.1 | 7.4 | 10.6 | 12.6 |
| 7 | 8 |  | 3.15 | 2.35 | -20.7 | -19.6 | 0.24 | 0.18 | 7.6 | 7.0 | 13.2 | 13.3 |
| 8 | 9 |  | 2.55 | 2.18 | -20.6 | -20.3 | 0.19 | 0.17 | 7.1 | 6.9 | 13.6 | 12.7 |
| 9 | 10 |  | 2.11 | 2.44 | -20.2 | -20.6 | 0.17 | 0.19 | 7.3 | 6.8 | 12.5 | 12.9 |
| 10 | 11 |  | 2.28 | 2.64 | -20.6 | -21.2 | 0.18 | 0.19 | 7.1 | 7.3 | 12.7 | 13.7 |
| 11 | 12 |  | 2.20 | 2.47 | -20.4 | -20.9 | 0.18 | 0.19 | 6.3 | 7.5 | 12.6 | 13.1 |
| 12 | 13 |  | 1.84 | 2.24 | -20.4 | -20.4 | 0.14 | 0.18 | 6.5 | 6.9 | 12.9 | 12.6 |
| 13 | 14 |  | 2.00 | 2.37 | -20.2 | -20.2 | 0.16 | 0.20 | 6.4 | 6.5 | 12.5 | 11.8 |
| 14 | 15 |  | 2.06 | 2.54 | -20.2 | -20.4 | 0.17 | 0.22 | 6.4 | 6.6 | 12.0 | 11.5 |
| 15 | 16 |  | 2.16 | 2.28 | -20.4 | -20.4 | 0.19 | 0.18 | 6.3 | 6.6 | 11.5 | 12.8 |
| 16 | 17 |  | 2.48 | 2.10 | -20.1 | -20.1 | 0.20 | 0.20 | 6.6 | 6.8 | 12.2 | 10.7 |
| 17 | 18 |  | 2.86 | 2.25 | -20.4 | -19.3 | 0.28 | 0.20 | 6.6 | 6.9 | 10.3 | 11.2 |
| 18 | 19 |  | 2.19 |  | -20.3 |  | 0.18 |  | 6.4 |  | 12.1 |  |
| 19 | 20 |  | 2.24 |  | -20.3 |  | 0.20 |  | 6.3 |  | 11.1 |  |
| 20 | 21 |  | 2.37 |  | -20.4 |  | 0.21 |  | 6.5 |  | 11.3 |  |
| 21 | 22 |  | 2.20 |  | -20.2 |  | 0.18 |  | 6.6 |  | 12.4 |  |
| 22 | 23 |  | 2.28 |  | -19.7 |  | 0.19 |  | 6.5 |  | 12.3 |  |
| 23 | 24 |  | 2.38 |  | -20.0 |  | 0.20 |  | 6.6 |  | 12.0 |  |

| Site: | SR |  |  |  |  |  |  |  |  |  |  |  |
| --- | --- | --- | --- | --- | --- | --- | --- | --- | --- | --- | --- | --- |
| Depth (cm) | |  | % C | | δ^13^ C | | % N | | δ^15^N | | C/N | |
| Top | bottom | Core | 1 | 2 | 1 | 2 | 1 | 2 | 1 | 2 | 1 | 2 |
| 0 | 1 |  | 3.30 | 3.51 | -20.4 | -19.4 | 0.37 | 0.38 | 8.4 | 8.3 | 8.8 | 9.4 |
| 1 | 2 |  | 3.32 | 3.29 | -20.5 | -20.0 | 0.36 | 0.35 | 8.0 | 8.1 | 9.1 | 9.5 |
| 2 | 3 |  | 3.22 | 3.78 | -20.5 | -19.6 | 0.35 | 0.40 | 8.2 | 8.2 | 9.3 | 9.4 |
| 3 | 4 |  | 3.10 | 3.48 | -20.3 | -20.0 | 0.32 | 0.38 | 8.3 | 8.2 | 9.6 | 9.2 |
| 4 | 5 |  | 3.19 | 3.83 | -20.6 | -19.8 | 0.31 | 0.36 | 8.2 | 8.2 | 10.1 | 10.5 |
| 5 | 6 |  | 3.07 | 3.44 | -20.4 | -19.7 | 0.33 | 0.36 | 8.3 | 8.2 | 9.4 | 9.5 |
| 6 | 7 |  | 3.13 | 3.40 | -20.5 | -19.3 | 0.32 | 0.34 | 8.1 | 8.2 | 9.9 | 10.0 |
| 7 | 8 |  | 2.97 | 3.39 | -20.5 | -19.9 | 0.30 | 0.35 | 8.0 | 8.1 | 9.9 | 9.7 |
| 8 | 9 |  | 2.92 | 3.45 | -19.7 | -20.3 | 0.28 | 0.36 | 8.1 | 7.9 | 10.3 | 9.6 |
| 9 | 10 |  | 2.78 | 2.96 | -20.4 | -20.5 | 0.26 | 0.30 | 7.9 | 8.1 | 10.7 | 9.9 |
| 10 | 11 |  | 3.60 | 3.07 | -20.0 | -19.6 | 0.31 | 0.29 | 7.9 | 8.1 | 11.5 | 10.6 |
| 11 | 12 |  | 3.08 | 2.49 | -20.2 | -19.0 | 0.27 | 0.21 | 8.1 | 8.0 | 11.2 | 12.0 |
| 12 | 13 |  | 3.67 | 2.90 | -17.8 | -18.3 | 0.28 | 0.24 | 8.0 | 8.3 | 13.2 | 12.3 |
| 13 | 14 |  | 2.51 | 3.27 | -18.4 | -18.0 | 0.21 | 0.25 | 8.0 | 8.1 | 12.2 | 12.9 |
| 14 | 15 |  | 2.53 | 2.71 | -19.0 | -18.9 | 0.22 | 0.24 | 7.8 | 8.5 | 11.5 | 11.3 |
| 15 | 16 |  | 2.92 | 2.53 | -18.4 | -18.8 | 0.22 | 0.23 | 7.7 | 8.2 | 13.3 | 10.9 |
| 16 | 17 |  | 2.74 | 2.29 | -19.7 | -19.3 | 0.21 | 0.21 | 7.3 | 8.2 | 13.2 | 11.2 |
| 17 | 18 |  | 2.43 | 2.71 | -18.5 | -18.6 | 0.20 | 0.23 | 7.4 | 8.2 | 11.9 | 11.7 |
| 18 | 19 |  | 2.44 | 2.50 | -17.9 | -18.3 | 0.20 | 0.23 | 7.3 | 8.0 | 12.3 | 11.1 |
| 19 | 20 |  | 2.09 | 2.03 | -19.2 | -19.6 | 0.19 | 0.20 | 7.1 | 8.5 | 10.9 | 10.0 |
| 20 | 21 |  | 2.19 | 2.02 | -19.5 | -18.1 | 0.20 | 0.18 | 6.8 | 6.8 | 10.7 | 11.2 |
| 21 | 22 |  | 1.99 |  | -18.6 |  | 0.17 |  | 7.3 |  | 11.5 |  |
| 22 | 23 |  | 1.78 | 1.78 | -18.6 | -18.8 | 0.15 | 0.00 | 7.0 |  | 12.0 | 1211.0 |
| 23 | 24 |  | 1.75 | 1.78 | -18.5 | -19.0 | 0.15 | 0.16 | 6.7 | 6.8 | 11.8 | 11.4 |
| 24 | 25 |  | 1.69 | 1.68 | -18.6 | -19.5 | 0.16 | 0.17 | 6.6 | 7.2 | 10.4 | 10.1 |
| 25 | 26 |  | 1.70 | 1.87 | -18.5 | -18.2 | 0.16 | 0.16 | 6.2 | 6.9 | 10.9 | 11.6 |
| 26 | 27 |  |  | 1.79 |  | -18.0 |  | 0.15 |  | 6.9 |  | 11.7 |
| 27 | 28 |  |  | 1.68 |  | -19.1 |  | 0.17 |  | 7.3 |  | 9.9 |
| 28 | 29 |  |  | 1.67 |  | -19.1 |  | 0.15 |  | 7.1 |  | 11.1 |
| 29 | 30 |  |  | 1.55 |  | -19.1 |  | 0.17 |  | 6.6 |  | 9.3 |
| 30 | 31 |  |  | 1.60 |  | -17.9 |  | 0.14 |  | 6.6 |  | 11.3 |

| Site | PP |  |  |  |  |  |  |  |  |  |  |  |
| --- | --- | --- | --- | --- | --- | --- | --- | --- | --- | --- | --- | --- |
| Depth (cm) | |  | % C | | δ^13^C | | % N | | δ^15^N | | C/N | |
| Top | bottom | Core | 2 | 3 | 2 | 3 | 2 | 3 | 2 | 3 | 2 | 3 |
| 0 | 1 |  | 3.10 | 2.85 | -19.3 | -20.1 | 0.27 | 0.27 | 7.6 | 7.9 | 11.5 | 10.5 |
| 1 | 2 |  | 3.07 | 3.19 | -19.7 | -18.2 | 0.29 | 0.28 | 7.2 | 7.8 | 10.4 | 11.5 |
| 2 | 3 |  | 2.97 | 2.99 | -19.7 | -20.4 | 0.26 | 0.28 | 7.5 | 7.8 | 11.3 | 10.8 |
| 3 | 4 |  | 2.93 | 3.01 | -20.2 | -20.1 | 0.27 | 0.29 | 7.4 | 7.8 | 11.0 | 10.5 |
| 4 | 5 |  | 3.01 | 3.09 | -20.0 | -20.0 | 0.29 | 0.29 | 8.0 | 7.7 | 10.5 | 10.8 |
| 5 | 6 |  | 2.90 | 3.44 | -20.0 | -18.3 | 0.27 | 0.29 | 7.8 | 7.9 | 10.8 | 12.0 |
| 6 | 7 |  | 3.08 | 3.11 | -20.3 | -19.3 | 0.30 | 0.28 | 7.8 | 7.5 | 10.4 | 11.1 |
| 7 | 8 |  | 3.11 | 3.01 | -20.0 | -20.3 | 0.29 | 0.28 | 7.9 | 7.6 | 10.9 | 10.8 |
| 8 | 9 |  | 3.05 | 3.05 | -20.6 | -20.8 | 0.29 | 0.27 | 7.8 | 7.2 | 10.7 | 11.1 |
| 9 | 10 |  | 3.02 | 3.07 | -20.3 | -21.0 | 0.28 | 0.27 | 7.7 | 7.2 | 10.8 | 11.3 |
| 10 | 11 |  | 3.11 | 2.99 | -20.0 | -20.9 | 0.26 | 0.28 | 7.7 | 7.2 | 12.1 | 10.8 |
| 11 | 12 |  | 3.02 | 3.04 | -21.0 | -20.8 | 0.26 | 0.26 | 7.4 | 7.4 | 11.7 | 11.5 |
| 12 | 13 |  | 3.03 | 3.04 | -21.0 | -21.7 | 0.26 | 0.27 | 7.5 | 7.4 | 11.6 | 11.1 |
| 13 | 14 |  | 3.02 | 3.03 | -21.1 | -21.6 | 0.25 | 0.27 | 7.1 | 7.3 | 12.1 | 11.4 |
| 14 | 15 |  | 3.05 | 2.91 | -21.2 | -21.4 | 0.26 | 0.26 | 7.5 | 7.2 | 11.8 | 11.3 |
| 15 | 16 |  | 2.89 | 2.94 | -21.0 | -21.0 | 0.24 | 0.25 | 7.6 | 7.3 | 11.8 | 11.7 |
| 16 | 17 |  | 3.17 | 2.95 | -19.7 | -20.6 | 0.24 | 0.24 | 7.3 | 7.2 | 13.1 | 12.4 |
| 17 | 18 |  | 3.46 | 2.95 | -20.0 | -20.4 | 0.23 | 0.24 | 7.4 | 7.3 | 14.8 | 12.5 |
| 18 | 19 |  | 4.07 | 2.64 | -20.8 | -20.5 | 0.21 | 0.22 | 6.9 | 7.5 | 19.2 | 12.0 |
| 19 | 20 |  | 3.16 | 2.46 | -21.6 | -18.4 | 0.22 | 0.20 | 6.9 | 7.5 | 14.1 | 12.2 |
| 20 | 21 |  | 3.65 | 2.29 | -21.8 | -19.4 | 0.25 | 0.21 | 6.9 | 7.1 | 14.4 | 11.0 |
| 21 | 22 |  | 3.23 | 2.39 | -21.2 | -20.4 | 0.25 | 0.21 | 7.4 | 7.2 | 12.9 | 11.3 |
| 22 | 23 |  | 3.48 | 2.32 | -17.9 | -18.9 | 0.22 | 0.22 | 7.3 | 7.2 | 15.9 | 10.7 |
| 23 | 24 |  | 4.94 | 2.16 | -18.2 | -20.2 | 0.23 | 0.22 | 6.8 | 7.4 | 21.4 | 10.0 |
| 24 | 25 |  | 2.30 | 2.13 | -20.9 | -19.8 | 0.22 | 0.22 | 7.4 | 7.2 | 10.3 | 9.8 |
| 25 | 26 |  | 2.26 | 2.21 | -19.6 | -19.5 | 0.22 | 0.22 | 7.4 | 7.4 | 10.3 | 10.0 |
| 26 | 27 |  | 2.11 | 2.06 | -20.4 | -20.4 | 0.22 | 0.21 | 7.4 | 7.3 | 9.8 | 9.7 |
| 27 | 28 |  | 2.19 | 2.09 | -19.7 | -19.6 | 0.22 | 0.21 | 7.4 | 7.2 | 9.9 | 9.8 |
| 28 | 29 |  | 2.13 | 2.10 | -20.3 | -19.2 | 0.22 | 0.21 | 7.4 | 7.1 | 9.6 | 9.9 |
| 29 | 30 |  | 2.13 | 2.04 | -20.4 | -20.0 | 0.22 | 0.21 | 7.3 | 7.2 | 9.8 | 9.6 |
| 30 | 31 |  | 2.14 | 2.03 | -19.5 | -19.9 | 0.21 | 0.21 | 7.3 | 7.0 | 10.1 | 9.6 |
| 31 | 32 |  | 2.08 | 2.02 | -20.3 | -20.2 | 0.22 | 0.22 | 7.4 | 7.2 | 9.5 | 9.0 |
| 32 | 33 |  | 2.12 | 2.05 | -20.3 | -20.0 | 0.22 | 0.22 | 7.5 | 7.2 | 9.5 | 9.4 |
| 33 | 34 |  | 2.09 | 2.04 | -20.5 | -20.2 | 0.22 | 0.21 | 7.4 | 7.3 | 9.5 | 9.6 |
| 34 | 35 |  |  | 1.95 |  | -20.3 |  | 0.21 |  | 7.2 |  | 9.5 |

| Site | QP |  |  |  |  |  |  |  |  |  |  |  |
| --- | --- | --- | --- | --- | --- | --- | --- | --- | --- | --- | --- | --- |
| Depth (cm) | |  | % C | | δ^13^C | | % N | | δ ^15^N | | C/N | |
| Top | bottom | Core | 1 | 3 | 1 | 3 | 1 | 3 | 1 | 3 | 1 | 3 |
| 0 | 1 |  | 1.73 | 1.75 | -20.8 | -20.7 | 0.17 | 0.18 | 7.8 | 7.8 | 10.4 | 9.7 |
| 1 | 2 |  | 1.79 | 1.77 | -20.7 | -21.0 | 0.18 | 0.19 | 7.9 | 7.6 | 10.1 | 9.2 |
| 2 | 3 |  | 1.83 | 1.80 | -20.7 | -20.8 | 0.17 | 0.18 | 7.8 | 7.6 | 10.5 | 9.8 |
| 3 | 4 |  |  | 1.80 |  | -20.9 |  | 0.19 |  | 7.6 |  | 9.7 |
|  | 5 |  | 1.65 |  | -20.8 |  | 0.15 |  | 7.4 |  | 10.7 |  |
| 4 | 5 |  |  | 1.71 |  | -20.9 |  | 0.17 |  | 7.8 |  | 10.1 |
| 5 | 6 |  | 1.57 | 1.66 | -20.9 | -20.7 | 0.15 | 0.17 | 7.4 | 7.8 | 10.5 | 10.0 |
| 6 | 7 |  | 1.50 | 1.64 | -20.9 | -20.6 | 0.14 | 0.16 | 7.3 | 7.8 | 10.8 | 10.5 |
| 7 | 8 |  | 1.53 | 1.55 | -21.0 | -21.0 | 0.15 | 0.15 | 7.0 | 6.9 | 10.6 | 10.4 |
| 8 | 9 |  | 1.47 | 1.59 | -20.9 | -21.0 | 0.14 | 0.15 | 7.5 | 7.1 | 10.4 | 10.8 |
| 9 | 10 |  | 1.48 | 1.68 | -21.0 | -21.1 | 0.14 | 0.15 | 7.3 | 7.1 | 10.7 | 11.0 |
| 10 | 11 |  | 1.49 | 1.52 | -20.8 | -20.9 | 0.14 | 0.15 | 7.7 | 8.1 | 10.4 | 10.3 |
| 11 | 12 |  | 1.53 | 1.51 | -20.8 | -20.9 | 0.15 | 0.15 | 7.1 | 7.9 | 10.3 | 10.3 |
| 12 | 13 |  | 1.52 | 1.52 | -20.7 | -20.8 | 0.14 | 0.15 | 7.0 | 6.7 | 10.5 | 10.4 |
| 13 | 14 |  | 1.48 | 1.56 | -20.9 | -20.4 | 0.14 | 0.15 | 7.1 | 7.7 | 10.9 | 10.5 |
| 14 | 15 |  | 1.49 | 1.49 | -20.8 | -20.3 | 0.13 | 0.14 | 7.1 | 6.9 | 11.2 | 10.8 |
| 15 | 16 |  | 1.46 | 1.51 | -20.6 | -20.4 | 0.13 | 0.14 | 6.8 | 6.4 | 11.0 | 10.8 |
| 16 | 17 |  | 1.48 | 1.46 | -20.8 | -20.7 | 0.14 | 0.14 | 7.6 | 6.6 | 10.7 | 10.8 |
| 17 | 18 |  | 1.49 | 1.48 | -20.7 | -20.7 | 0.13 | 0.14 | 7.2 | 6.7 | 11.7 | 10.6 |
| 18 | 19 |  | 1.47 | 1.50 | -20.5 | -20.6 | 0.13 | 0.14 | 7.0 | 6.9 | 11.0 | 10.7 |
| 19 | 20 |  | 1.48 | 1.51 | -20.5 | -20.5 | 0.13 | 0.14 | 6.9 | 6.9 | 11.3 | 10.5 |
| 20 | 21 |  | 1.45 | 1.49 | -20.6 | -20.6 | 0.13 | 0.14 | 6.7 | 6.9 | 10.9 | 10.9 |
| 21 | 22 |  | 1.44 | 1.48 | -20.6 | -20.5 | 0.13 | 0.14 | 6.6 | 7.1 | 11.2 | 10.6 |
| 22 | 23 |  | 1.44 | 1.42 | -20.7 | -20.5 | 0.13 | 0.11 | 6.8 | 6.8 | 11.2 | 12.5 |
| 23 | 24 |  | 1.41 | 1.43 | -20.8 | -20.5 | 0.13 | 0.12 | 6.6 | 6.8 | 11.2 | 12.1 |
| 24 | 25 |  | 1.41 | 1.46 | -20.5 | -20.5 | 0.13 | 0.13 | 7.0 | 6.9 | 10.9 | 11.5 |
| 25 | 26 |  | 1.40 | 1.43 | -20.5 | -20.7 | 0.14 | 0.12 | 6.7 | 6.6 | 10.4 | 11.9 |
| 26 | 27 |  | 1.44 | 1.36 | -20.8 | -20.4 | 0.13 | 0.12 | 6.9 | 7.4 | 11.0 | 11.7 |
| 27 | 28 |  | 1.41 | 1.45 | -20.7 | -20.6 | 0.13 | 0.13 | 6.7 | 7.0 | 10.8 | 11.2 |
| 28 | 29 |  | 1.46 | 1.51 | -20.5 | -20.6 | 0.14 | 0.14 | 6.2 | 6.9 | 10.4 | 11.0 |
| 29 | 30 |  | 1.46 | 1.50 | -20.6 | -20.8 | 0.13 | 0.14 | 6.5 | 6.8 | 10.8 | 10.7 |
| 30 | 31 |  | 1.50 |  | -20.6 |  | 0.14 |  | 6.6 |  | 11.1 |  |
| 31 | 32 |  | 1.51 |  | -20.5 |  | 0.14 |  | 6.8 |  | 10.8 |  |
| 32 | 33 |  | 1.38 |  | -20.7 |  | 0.13 |  | 6.6 |  | 10.5 |  |
| 33 | 34 |  | 1.29 |  | -20.7 |  | 0.13 |  | 6.6 |  | 10.3 |  |
| 34 | 35 |  | 1.40 |  | -20.6 |  | 0.13 |  | 6.6 |  | 10.6 |  |
| 35 | 36 |  | 1.48 |  | -20.7 |  | 0.14 |  | 6.7 |  | 10.9 |  |
| 36 | 37 |  | 1.54 |  | -20.5 |  | 0.14 |  | 6.5 |  | 11.0 |  |
| 37 | 38 |  | 1.60 |  | -20.5 |  | 0.15 |  | 6.7 |  | 10.7 |  |

Figure S1. Dated profiles of Mo in Narragansett Bay sediment cores
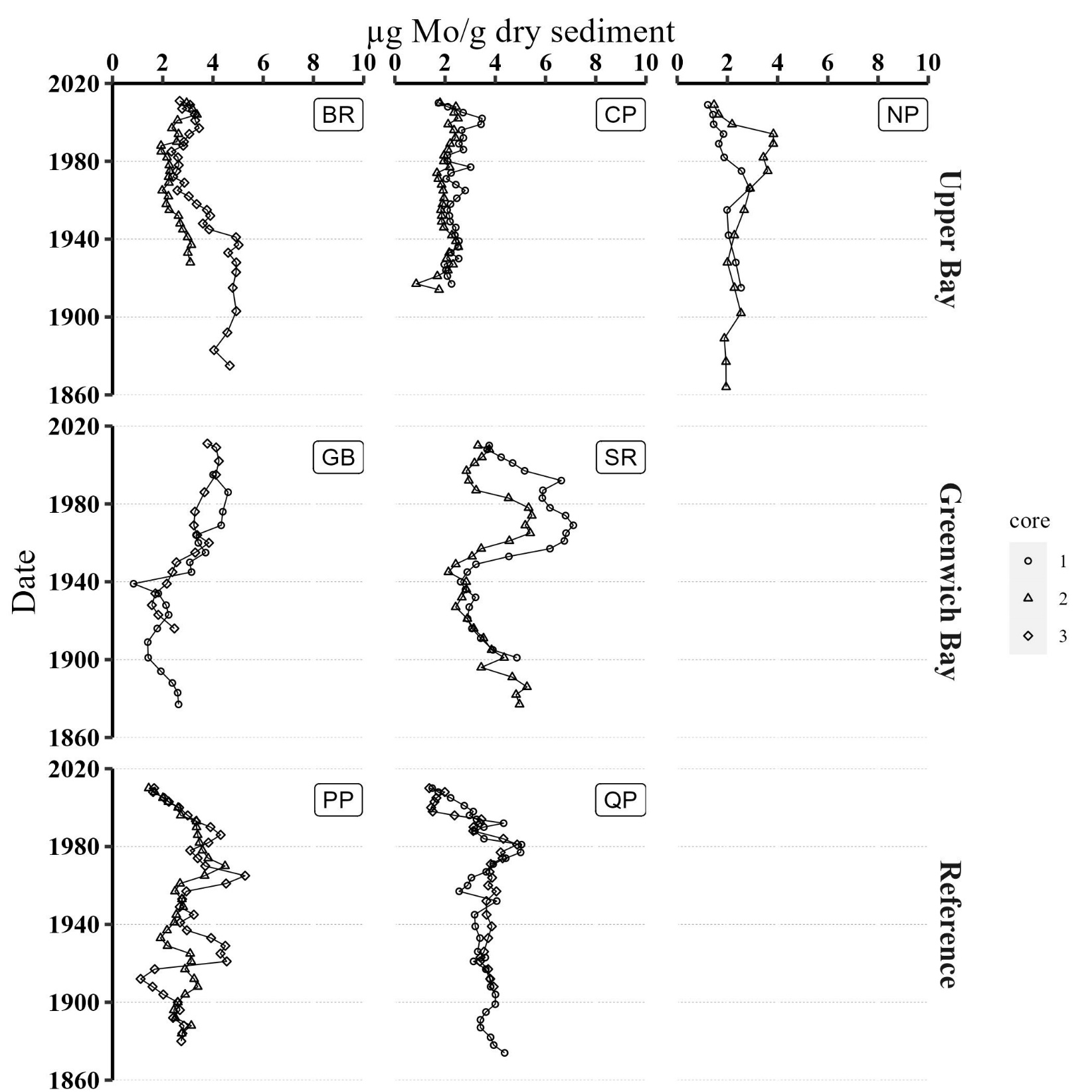


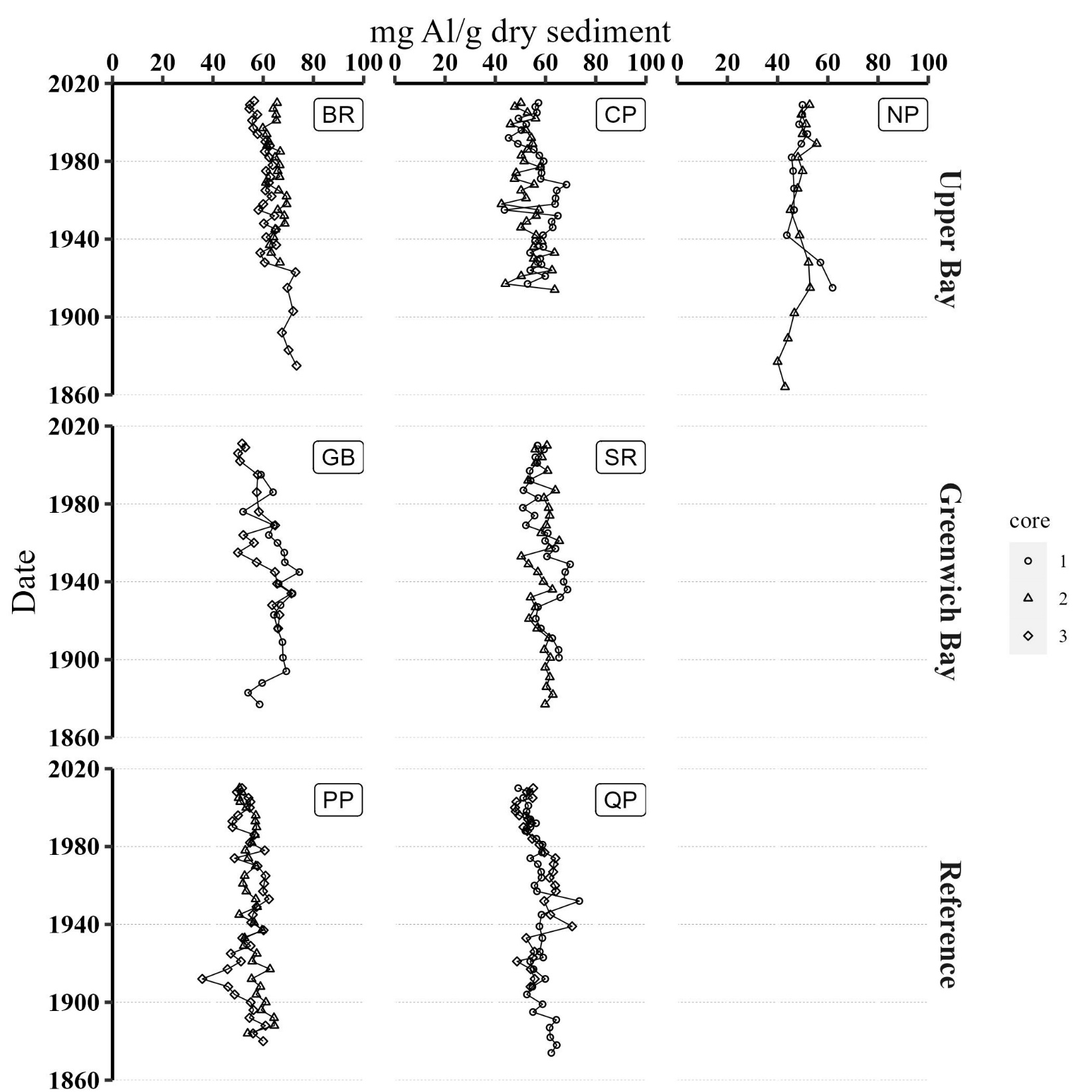
Figure S2. Dated profiles of Al in Narragansett Bay sediment cores


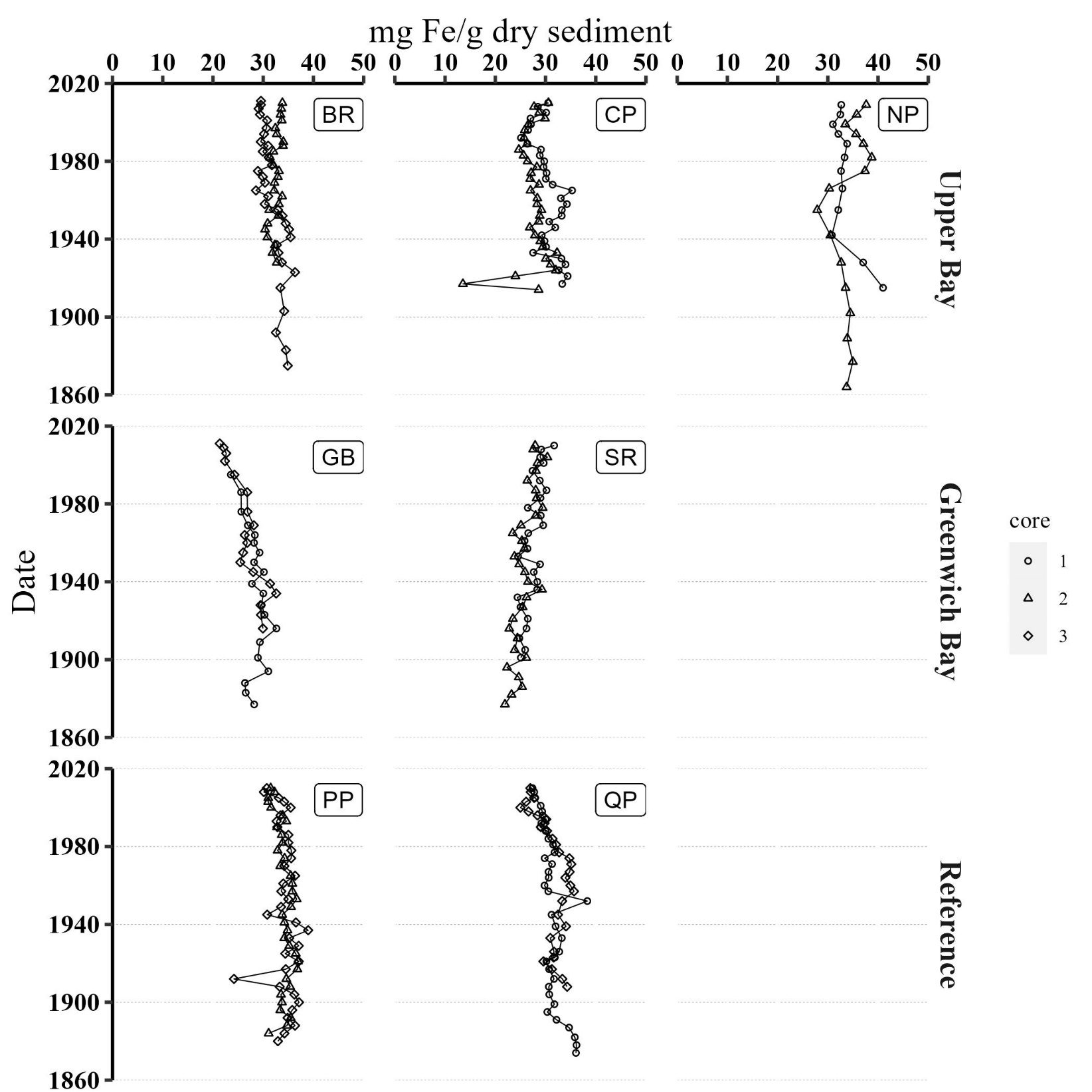
Figure S3. Dated profiles of Fe in Narragansett Bay sediment cores


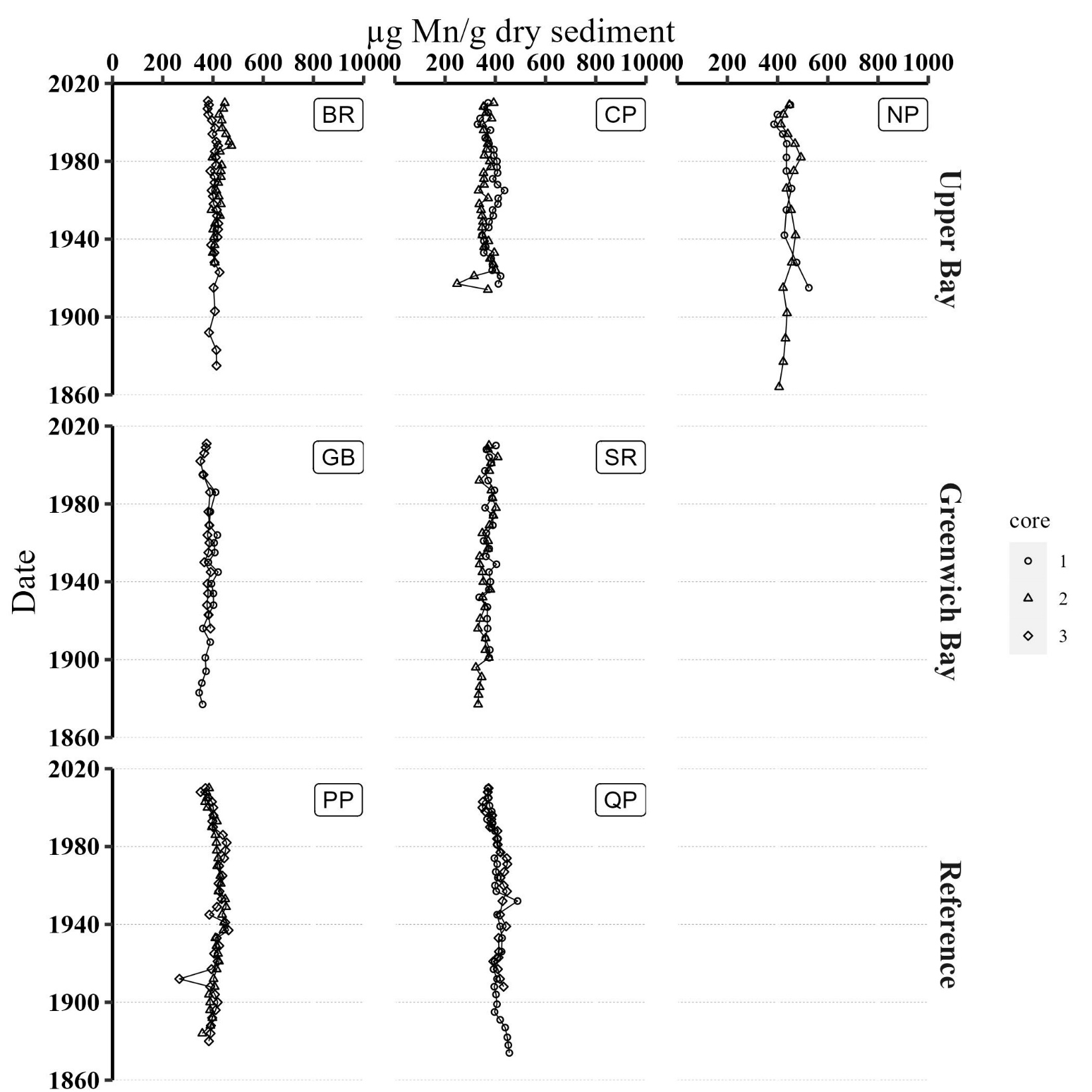
Figure S4. Dated profiles of Mn in Narragansett Bay sediment cores


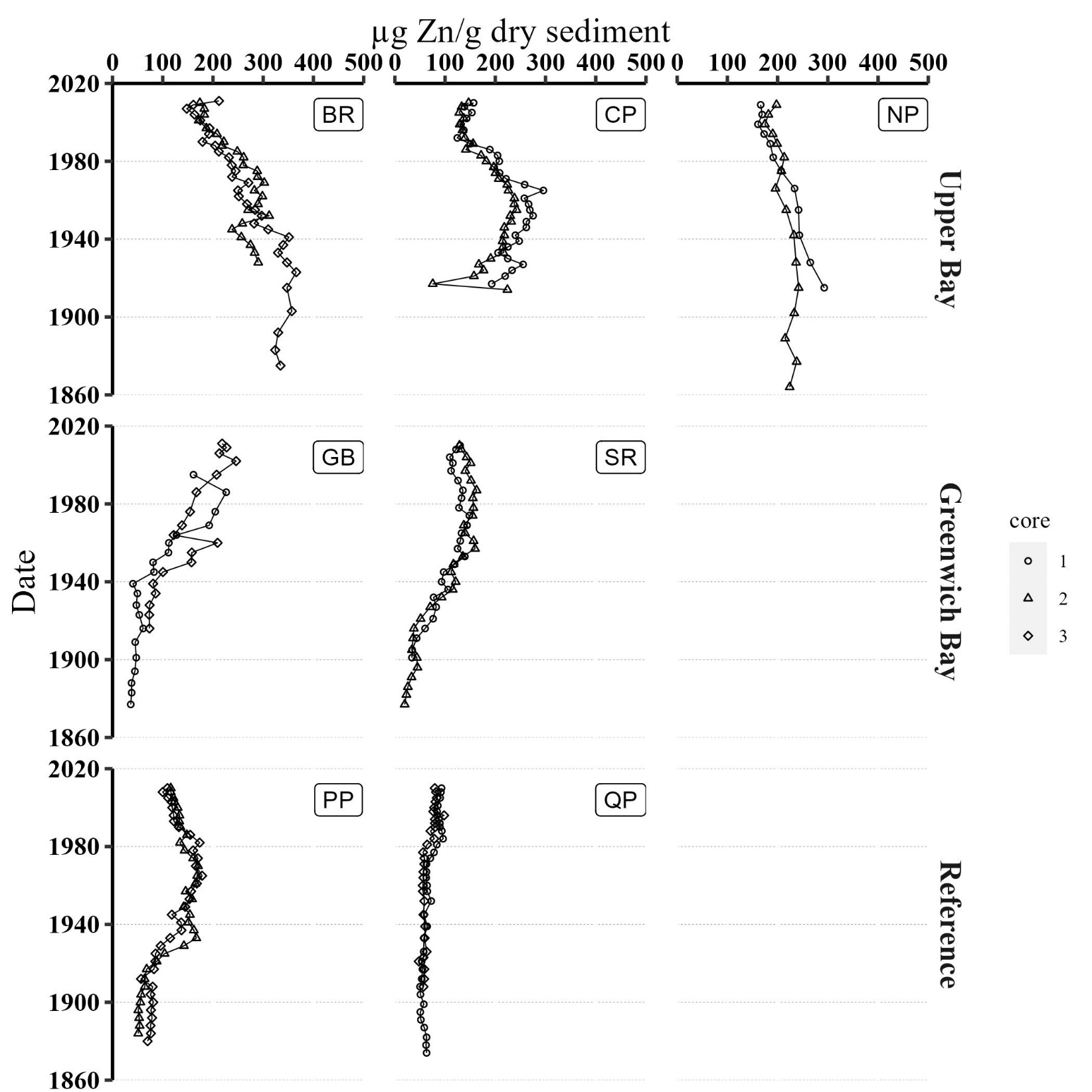
Figure S5. Dated profiles of Zn in Narragansett Bay sediment cores

Figure S6. Dated profiles of Cu
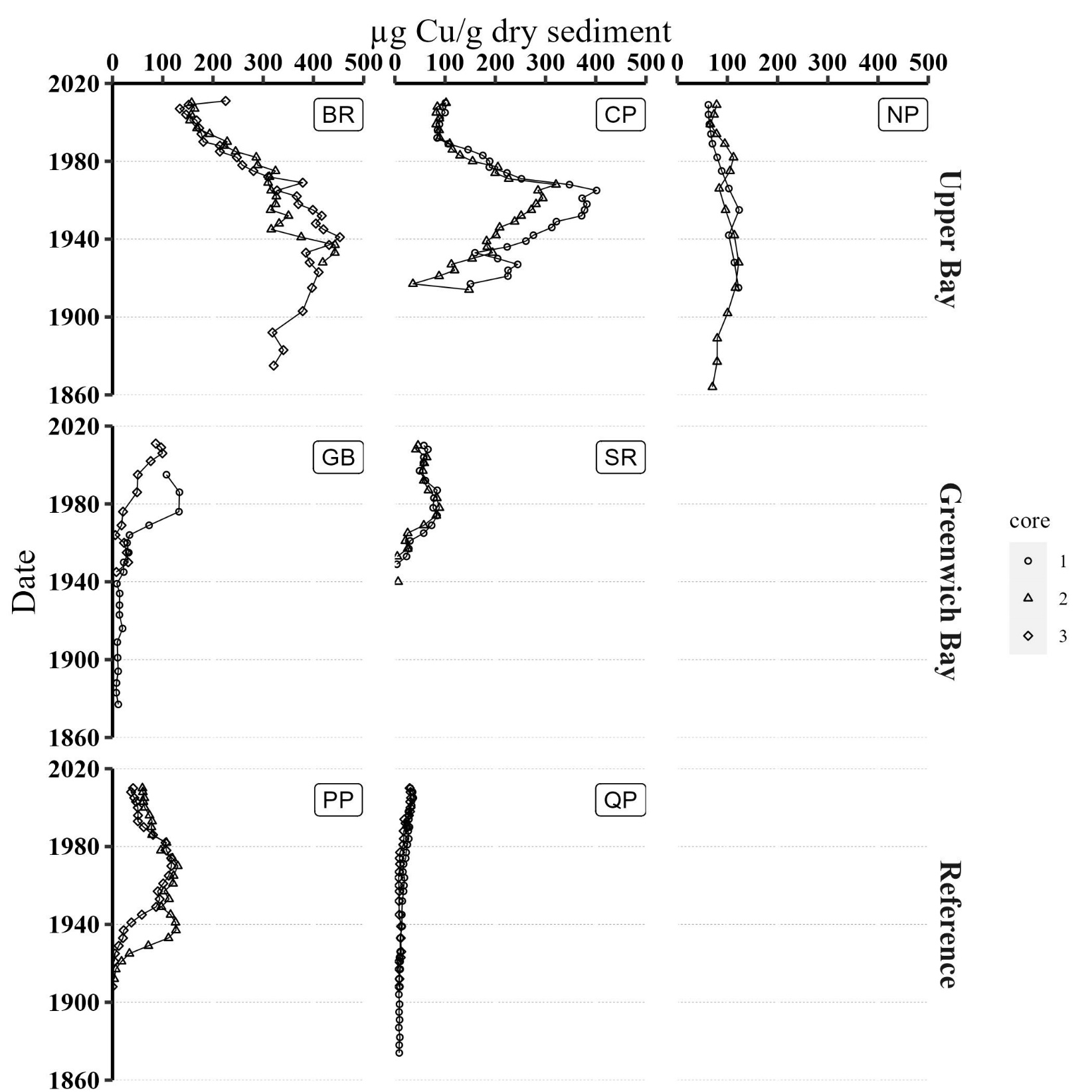
 in Narragansett Bay sediment cores


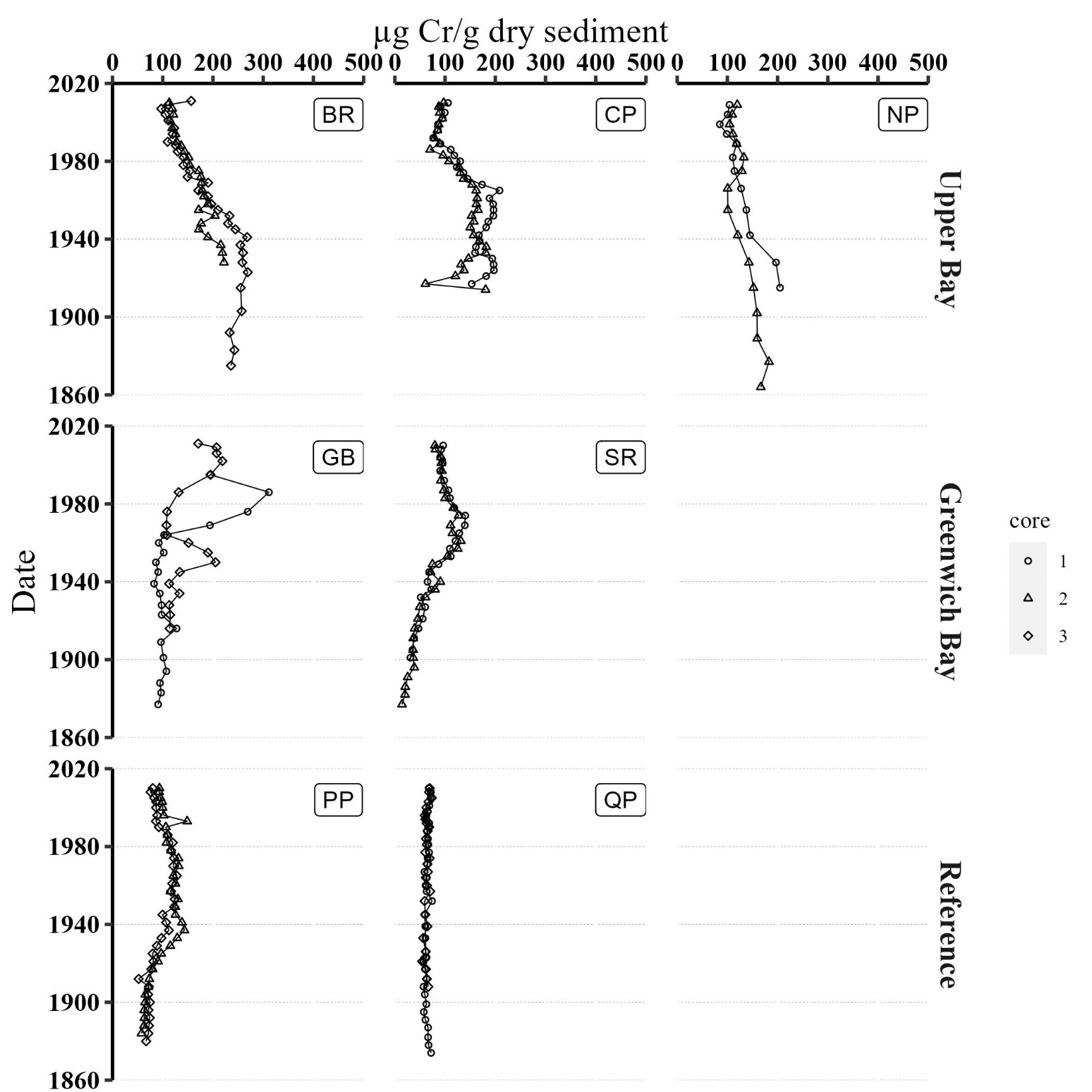
Figure S7. Dated profiles of Cr in Narragansett Bay sediment cores


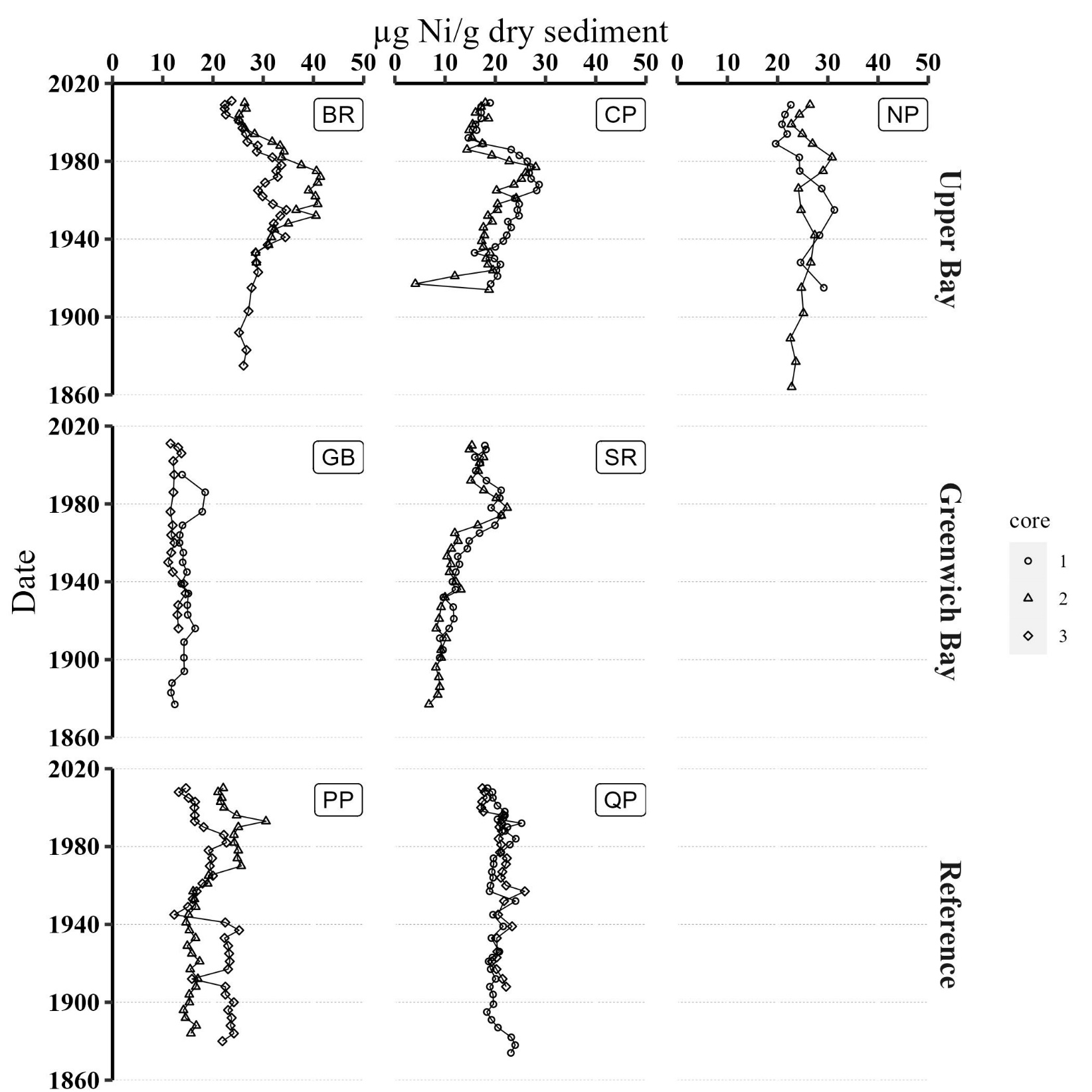
Figure S8. Dated profiles of Ni in Narragansett Bay sediment cores

1. * Corresponding author. E-mail address: [boothman.warren@epa.gov](mailto:boothman.warren@epa.gov). Postal address: U.S. EPA Office of Research and Development, Center for Environmental Measurement and Modeling, Atlantic Coastal Environmental Sciences Division, 27 Tarzwell Drive, Narragansett, RI, USA 02882 [↑](#footnote-ref-1)
